# Supplementary material for: Metabolic Roles of Uncultivated Bacterioplankton Lineages in the Northern Gulf of Mexico “Dead Zone”
Source: mBio. 2017 Sep 12;8(5):e01017-17. doi: 10.1128/mBio.01017-17 (PMC5596340; doi:10.1128/mBio.01017-17)
Supplement: TEXT S1 [file mbo004173471s1.docx]

**Supplemental Information**

**Metabolic roles of uncultivated bacterioplankton lineages in the northern Gulf of Mexico “Dead Zone”**

J. Cameron Thrash^*^, Kiley W. Seitz, Brett J. Baker^*^, Ben Temperton, Lauren E. Gillies, Nancy N. Rabalais, Bernard Henrissat, and Olivia U. Mason

*Correspondence:

JCT [thrashc@lsu.edu](mailto:thrashc@lsu.edu)

BJB [acidophile@gmail.com](mailto:acidophile@gmail.com)

**Supplemental Text**

***Taxonomic assignments and estimated genome sizes***

The six MGII genomes spanned a range of GC content from 41-62%, which may indicate the presence of sublineages, although their clustering within the ribosomal protein tree did not correspond with GC content (Fig. S1). Average amino acid identity (AAI) supported a close relationship between Bins 14, 17-1, and 18, congruent with the ribosomal phylogeny (Table S1). Expected complete genome sizes, based on estimated completion percentages, were between 1.7 and 3.2 Mbp (Table 1). Within SAR406, GC content groupings did correspond to phylogenetic inference. A low GC group (Group A) made up of Bins 51-1, and 51 with 39% and 41% GC, respectively, and a higher GC group (Group B) with Bins 45-2, 45, and 45-1 having 46%, 47%, and 49% GC, respectively. This pattern matched the ribosomal protein tree phylogeny where Bins 45-1 and 45-2 were sister and separated by large branch lengths from Bin 51-1 (Fig. S1). Bins 45 and 51 could not be included in the tree because of missing ribosomal proteins, but they were grouped with SAR406 through 16S rRNA genes, markers on other contigs like the RNA polymerase in Bin 45, similar patterns of protein family expansions (see below), based on their bin of origin, and via AAI calculations (Table S1). During quality control, Bin 51-1 was separated out of Bin 51, and Bins 45-1 and 45-2 out of Bin 45. The pairwise identities of the 16S rRNA genes between bins also support the 45/45-1 and 51/51-1 groupings, as does AAI, where Group A were more similar to each other than Group B, and vice versa (Table S1). The aggregate data therefore supported Bins 45 and 51 as SAR406. Based on 16S rRNA gene phylogeny (Fig. S4), Group A are sister to the Arctic96B-7 clade, and Group B form a monophyletic group with the early diverging SHBH1141 clade identified by Wright and colleagues (1). Expected genome sizes for SAR406 Group A were 2.6-2.7 Mbp and for Group B, 2.8-3.5 Mbp (Table 1). The three SAR202 genomes had very similar GC content (52-53%), were tightly grouped on the ribosomal protein tree on their own branch as early diverging members of the Chloroflexi (Fig. S1), and closely related- possibly at the level of Genus- based on AAI (2) (Table S1). We classified these genomes as subclade I based on 16S rRNA gene phylogeny (Fig. S5), which distinguishes them from the subclade III and V genomes recently reported (3). Estimated complete genome sizes were between 2.2-3.0 Mbp (Table 1). These genomes also had the lowest coding density of all the groups: 88-91% (Table S1).

Candidate Phylum lineages had fewer representative genomes. A single genome from Parcubacteria had a GC content of 52% and the two Perigrinibacteria genomes had 39 and 45% GC content. Both of these groups had well-supported positions in the ribosomal protein tree (Fig. S1) that were corroborated by BLASTP-derived annotation of encoded proteins against the NCBI nr database (Fig. S3). We estimated complete genome sizes for Parcubacteria and Perigrinibacteria at near 1.5 Mbp (Table 1). Bins 50 and 48 grouped with WS3 bacteria in the ribosomal protein tree, although their relative positions inside the clade were poorly supported at the internal nodes. AAI generally supports the designation of these two bins in the same phylum (4) (Table S1), as does their relationship in the ribosomal protein tree. However, the nearly complete Bin 50 16S rRNA gene sequence had best blast hits to the PAUC34f CP in the GreenGenes database. 16S rRNA gene phylogenetic inference grouped Bin 50 with other PAUC34f sequences sister to the entire WS3 clade (Fig. S6), conflicting with the ribosomal protein phylogeny. The lack of PAUC34f genomes makes it possible that the ribosomal protein tree grouping of Bins 50 and 48 occurred simply due to inadequate taxon selection, as these are nearby clades in the 16S rRNA gene tree. Although a rare group, PAUC34f was more abundant than WS3 in the amplicon dataset and we detected 18 PAUC34f operational taxonomic units (OTUs) compared to three assigned to WS3 (5), so reconstruction of PAUC34f genomes was more likely than WS3. Bin 13 grouped with genomes designated as ACD39, a group previously identified in an aquifer (6). However, Bin 13 was on a long branch and the relationship was poorly supported (Fig. S1). For the Bin 13 and PAUC34f genomes, blast-based identification via best hit annotation did not return clear results- hit annotations included a wide variety of taxa (Fig. S3). These results are consistent with what we would expect from novel genomes that have few or no members in the database, but this analysis did not directly corroborate either the ribosomal protein tree phylogeny for all three bins, or the 16S rRNA gene phylogeny for Bin 50. We therefore consider the designation of Bins 50/48 as PAUC34f, and Bin 13 as ACD39, putative. Bins 50 and 48 had 58 and 55% GC content, and larger estimated complete genomes than the others at 6.3 and 4.9 Mbp, respectively. Bin 13 also had a large expected genome size at 4.8 Mbp, and GC content of 47%.

**Supplemental Tables and Figures**

**Table S1.** Spreadsheet (Table_S1.xlsx) containing information on taxonomy, CheckM results, IMG statistics, partial metabolic reconstruction, transporter classifications, CAZy predictions, sample chemical data, RPKM values and gene neighborhoods for WS3 cytochrome c oxidases and *nrfA* genes from SAR406 and WS3. Due to its size, this is hosted at http://thethrashlab.com/publications.

**Figure S1.** Maximum likelihood tree of concatenated ribosomal protein coding genes. Values at internal nodes indicate bootstrap support (n=100). Scale bar indicates changes per position.

**Figure S2.** Phylogenetic placement of bins based on CheckM.

**Figure S3.** Annotations of protein-coding gene sequence best blastp hits in the nr database, divided into quartiles by bit score, for CP bacteria.

**Figure S4.** SAR406 16S rRNA gene phylogeny. Genes recovered from assembled bins have 45* or 51* designations. Values at nodes indicate Shimodaira-Hasegawa “like” values (7). Scale bar indicates changes per position.

**Figure S5.** SAR202 16S rRNA gene phylogeny. Genes recovered from assembled bins are indicated as 43-*. Subclades are designated according to Morris et al. 2004, and the tree is rooted according to Figure 1 in that publication. Values at nodes indicate Shimodaira-Hasegawa “like” values (7). Scale bar indicates changes per position.

**Figure S6.** 16S rRNA gene phylogeny of the WS3 clade (8) with added PAUC34f sequences from the GreenGenes database and the Bin 50 sequence. Tree is rooted on the Archaea according to Fig. S1 in Farag et al. 2017. Values at nodes indicate Shimodaira-Hasegawa “like” values (7). Scale bar indicates changes per position.

**Figure S7.** Metagenomic RPKM values for each group, comprised of aggregated values for each bin within the group. Values are plotted according to sample and colored according to the dissolved oxygen (DO) concentration from where the sample was taken.

**Figure S8.** Evaluation of predicted nrfA genes in SAR406 and Bins 50/48. A) Phylogenetic tree of predicted *nrfA* genes. Additional taxa are from Figure 3 in Welch et al., 2014. The tree was rooted at the midpoint. Values at nodes indicate Shimodaira-Hasegawa “like” values (7). Scale bar indicates changes per position. B&C) Conserved catalytic motifs within the *nrfA* gene. B) Black square surrounds the first heme-binding CXXCK/CXXCH motif. C) Black square surrounds the catalytic KXQH/KXRH motif. The alignment follows highlighting found in Welsh *et al.*, 2014 (9). All genes numbers from this study are indicated as 26536*, corresponding to rows 64 and 66-68.

**Additional Supplemental Information** such as Table S1, scripts, workflows, and key files, including fasta files for each tree, are provided as a link hosted at the Thrash Lab website: http://thethrashlab.com/publications.

**References Cited**

1. Wright JJ, Mewis K, Hanson NW, Konwar KM, Maas KR, Hallam SJ. 2014. Genomic properties of Marine Group A bacteria indicate a role in the marine sulfur cycle. The ISME Journal 8:455-468.

2. Konstantinidis KT, Tiedje JM. 2007. Prokaryotic taxonomy and phylogeny in the genomic era: advancements and challenges ahead. Current Opinion in Microbiology 10:504-509.

3. Landry Z, Swan BK, Herndl GJ, Stepanauskas R, Giovannoni SJ. 2017. SAR202 Genomes from the Dark Ocean Predict Pathways for the Oxidation of Recalcitrant Dissolved Organic Matter. mBio 8:17.

4. Konstantinidis KT, Tiedje JM. 2005. Towards a genome-based taxonomy for prokaryotes. Journal of bacteriology 187:6258-6264.

5. Gillies LE, Thrash JC, deRada S, Rabalais NN, Mason OU. 2015. Archaeal enrichment in the hypoxic zone in the northern Gulf of Mexico. Environmental Microbiology 17:3847.

6. Wrighton KC, Castelle CJ, Wilkins MJ, Hug LA, Sharon I, Thomas BC, Handley KM, Mullin SW, Nicora CD, Singh A, Lipton MS, Long PE, Williams KH, Banfield JF. 2014. Metabolic interdependencies between phylogenetically novel fermenters and respiratory organisms in an unconfined aquifer. The ISME Journal 8:1452-1463.

7. Price MN, Dehal PS, Arkin AP. 2010. FastTree 2--approximately maximum-likelihood trees for large alignments. PLOS ONE 5:e9490.

8. Farag IF, Youssef NH, Elshahed MS. 2017. Global Distribution Patterns and Pangenomic Diversity of the Candidate Phylum “Latescibacteria” (WS3). Applied and Environmental Microbiology 83: e00521-17.

9. Welsh A, Chee-Sanford JC, Connor LM, Löffler FE, Sanford RA. 2014. Refined NrfA Phylogeny Improves PCR-Based nrfA Gene Detection. Applied and Environmental Microbiology 80:2110-2119.
