# Supplementary figures and images for: Metabolic Roles of Uncultivated Bacterioplankton Lineages in the Northern Gulf of Mexico “Dead Zone”
Source: mBio. 2017 Sep 12;8(5):e01017-17. doi: 10.1128/mBio.01017-17 (PMC5596340; doi:10.1128/mBio.01017-17)

Annotation count

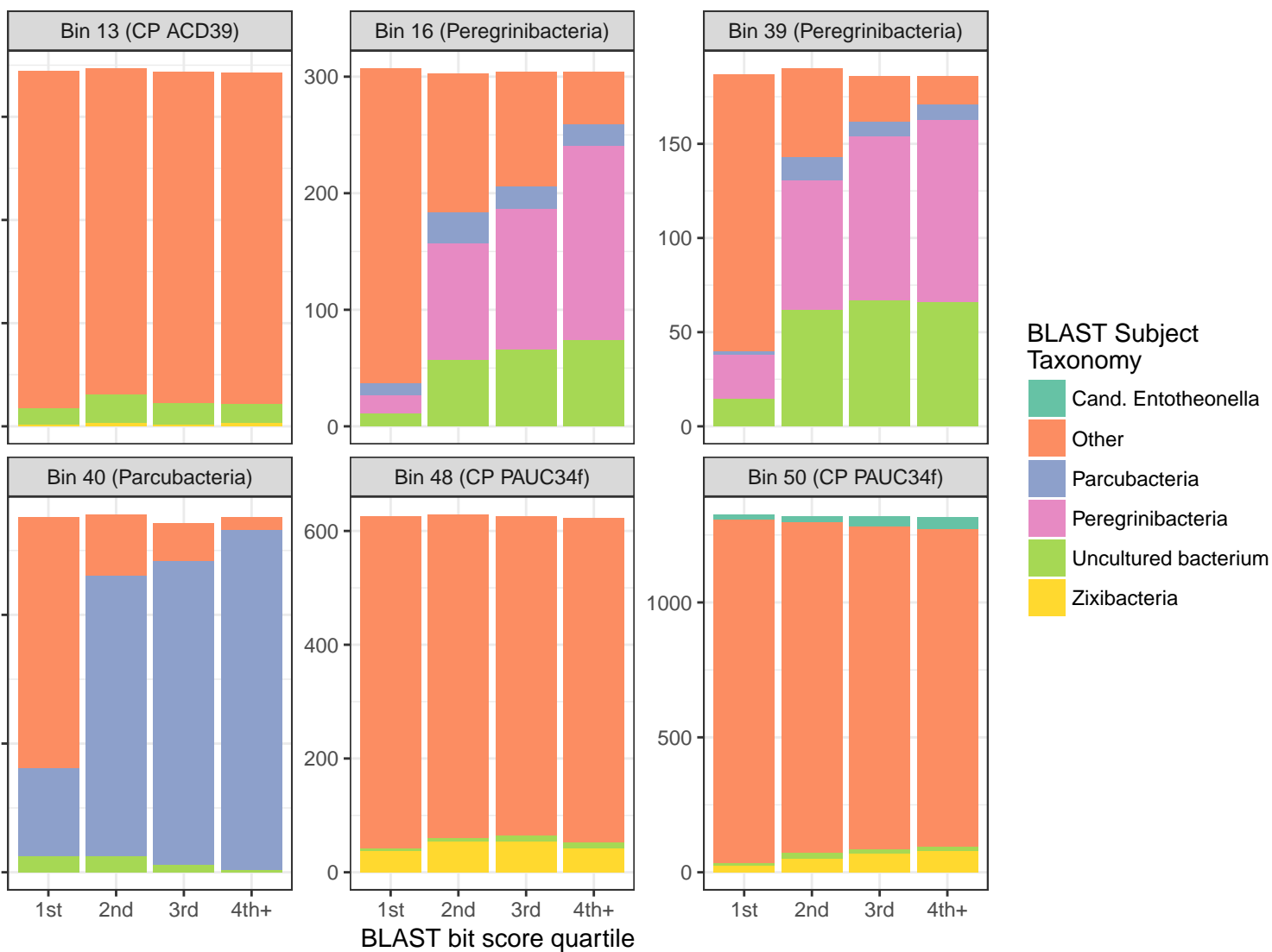

Supplement: FIG S3 [file mbo004173471sf3.pdf]

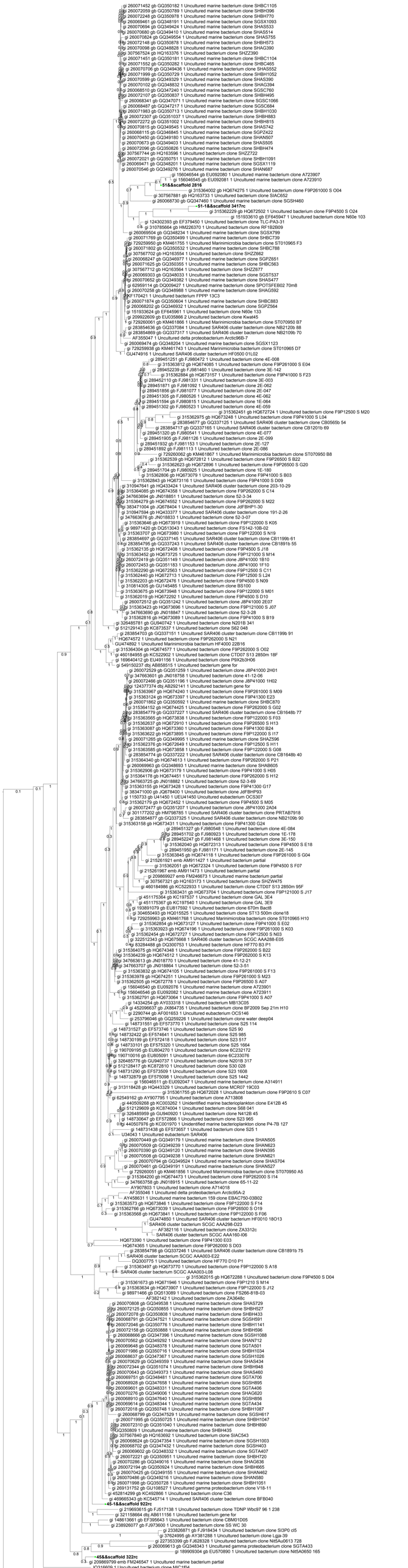

Supplement: FIG S4 [file mbo004173471sf4.pdf]

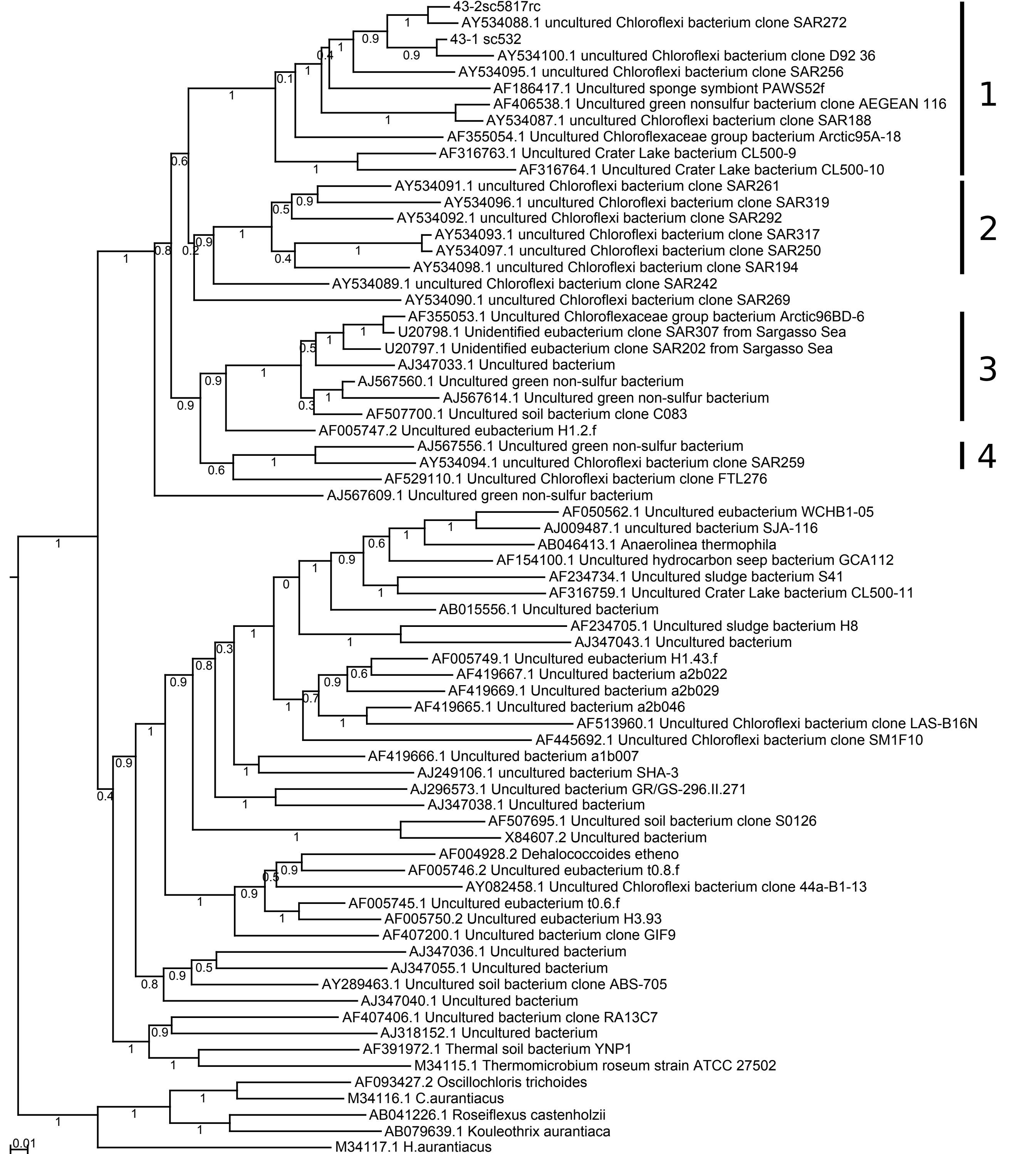

Supplement: FIG S5 [file mbo004173471sf5.pdf]

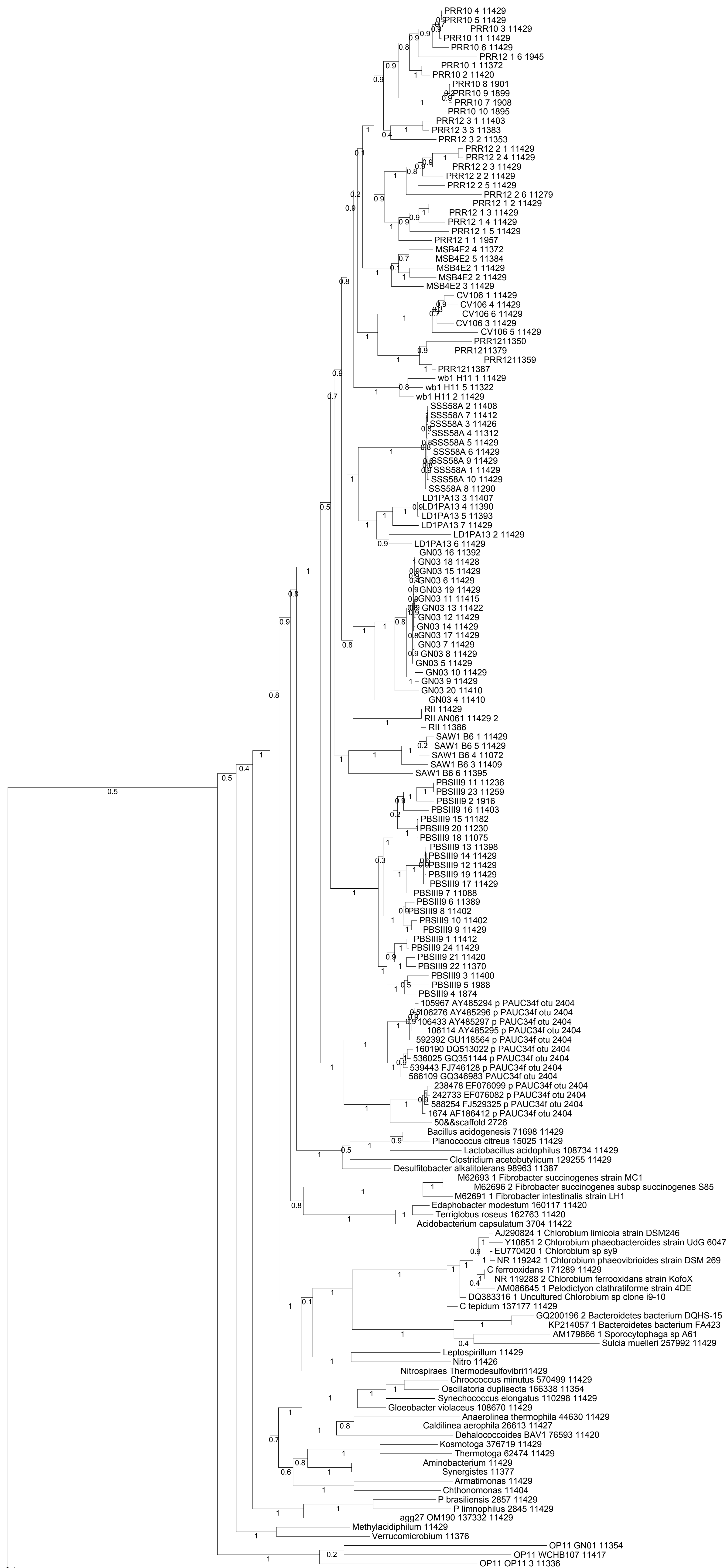

Supplement: FIG S6 [file mbo004173471sf6.pdf]

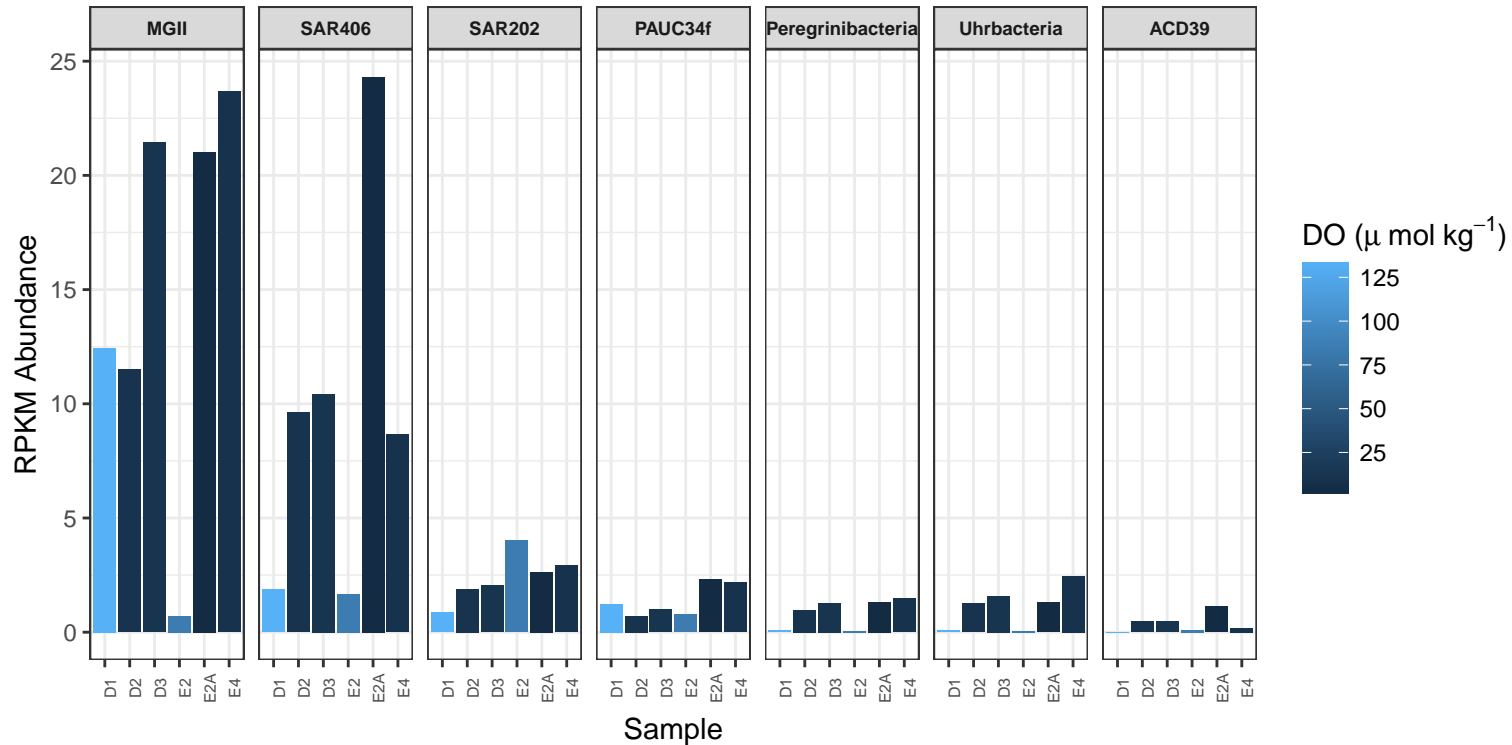

Supplement: FIG S7 [file mbo004173471sf7.pdf]

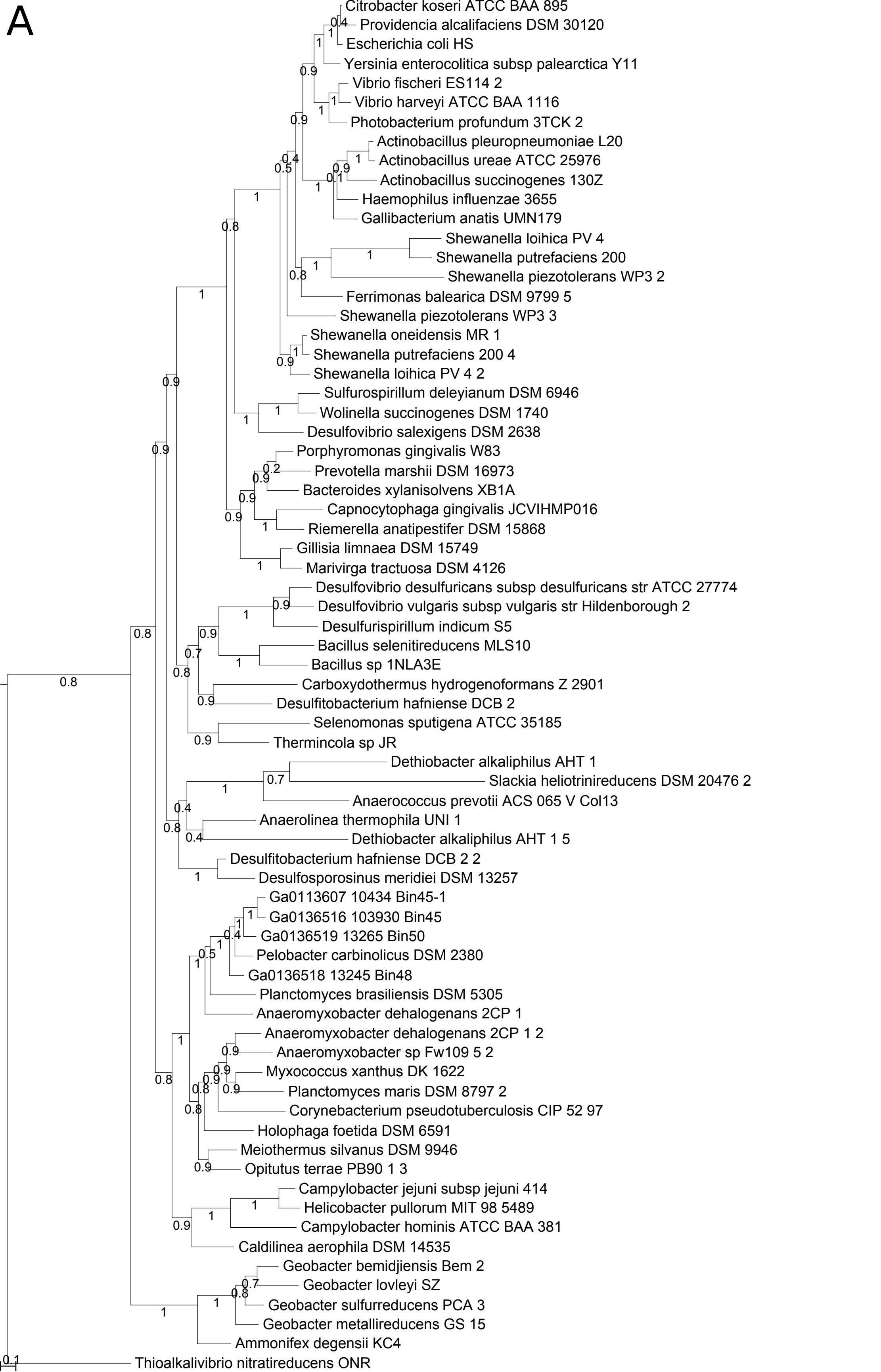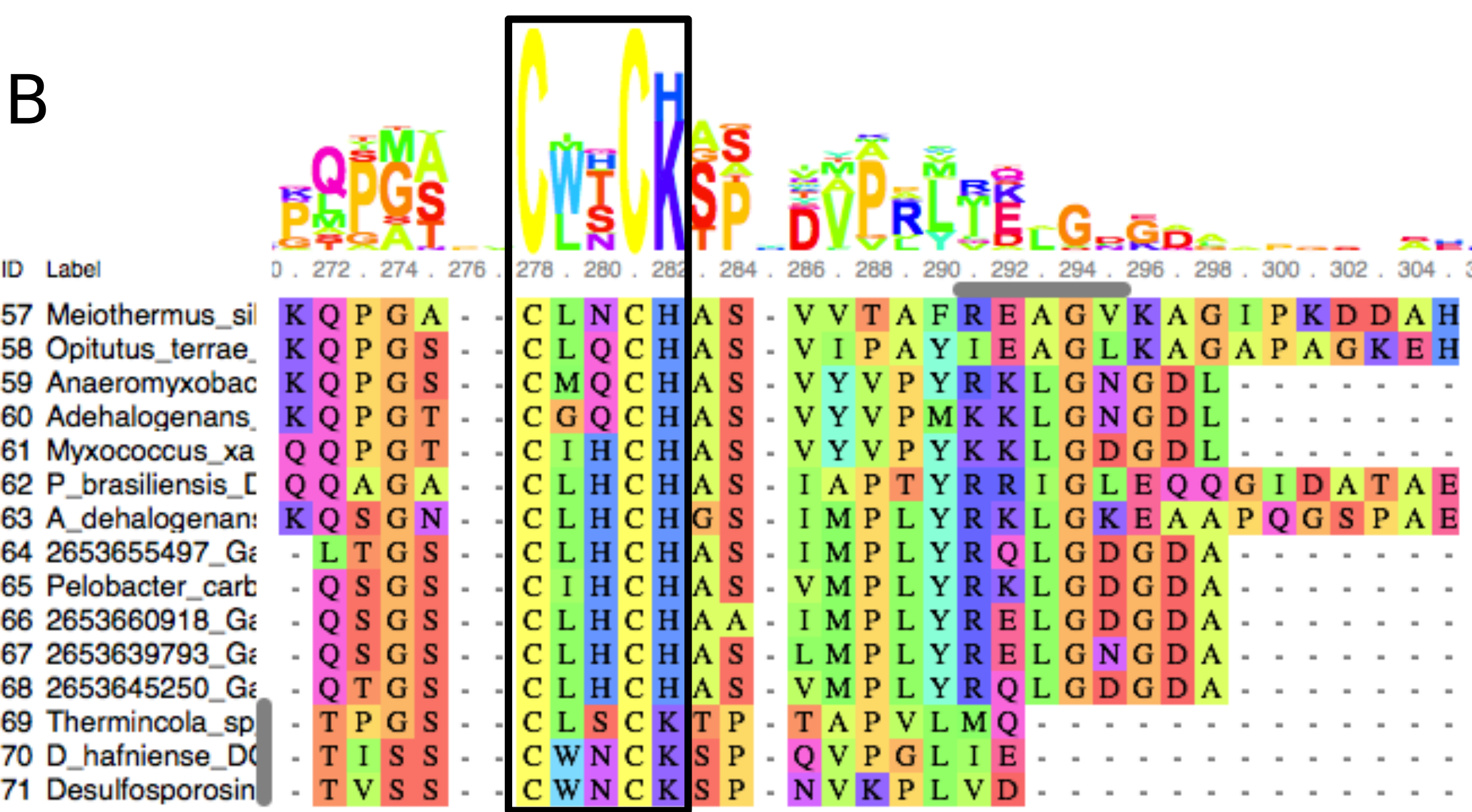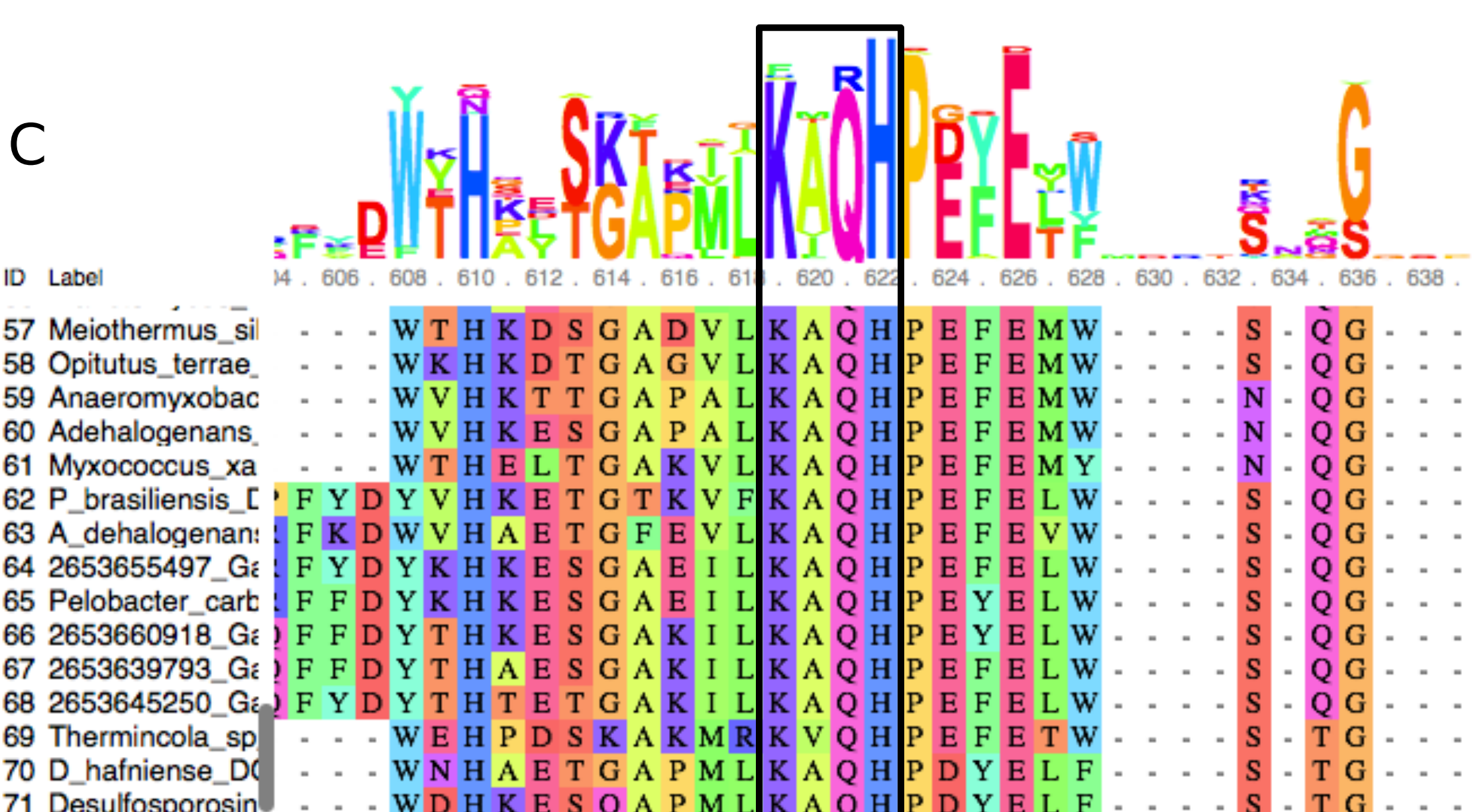

Supplement: FIG S8 [file mbo004173471sf8.pdf]
