# Supplementary material for: Metabolic Roles of Uncultivated Bacterioplankton Lineages in the Northern Gulf of Mexico “Dead Zone”
Source: mBio. 2017 Sep 12;8(5):e01017-17. doi: 10.1128/mBio.01017-17 (PMC5596340; doi:10.1128/mBio.01017-17)

Figure S1

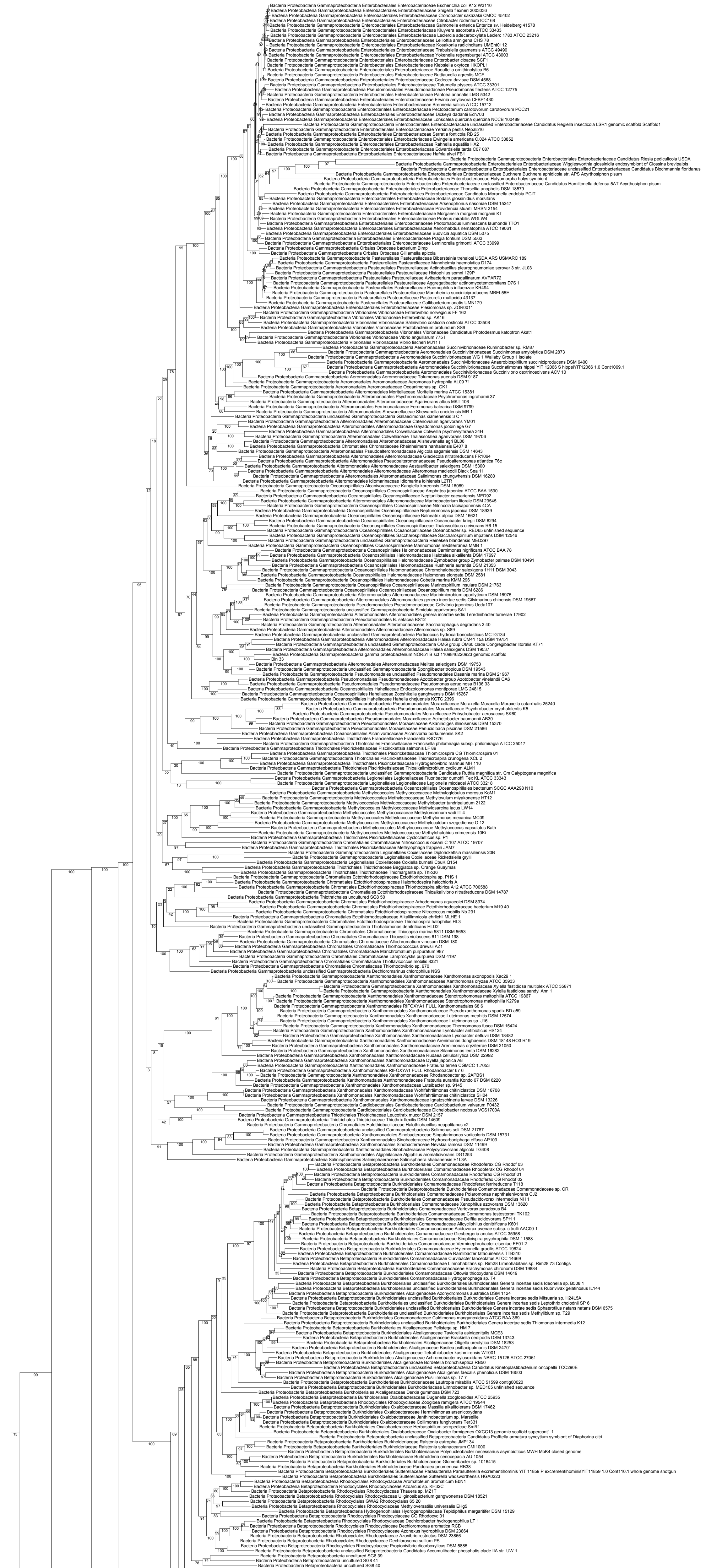

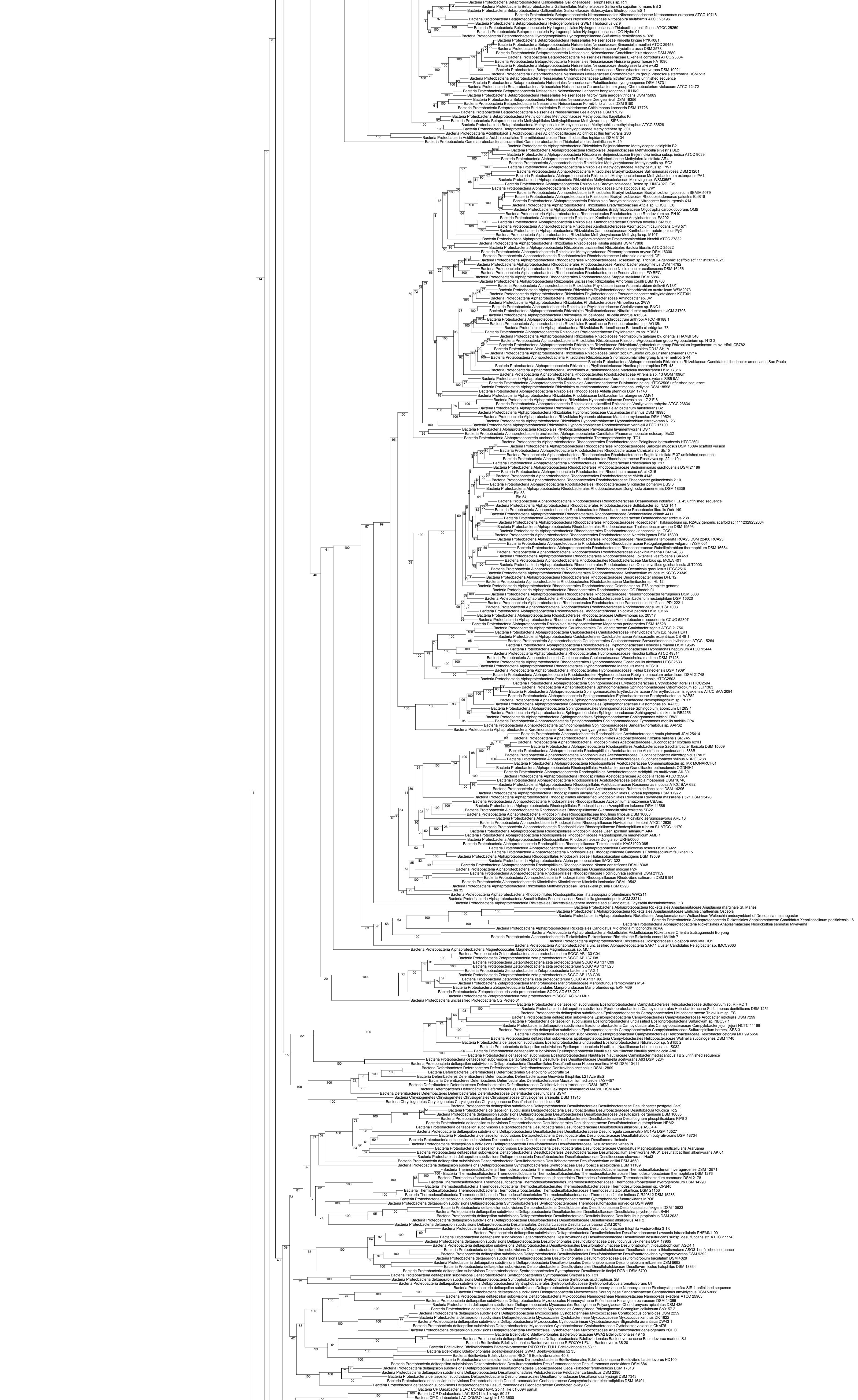

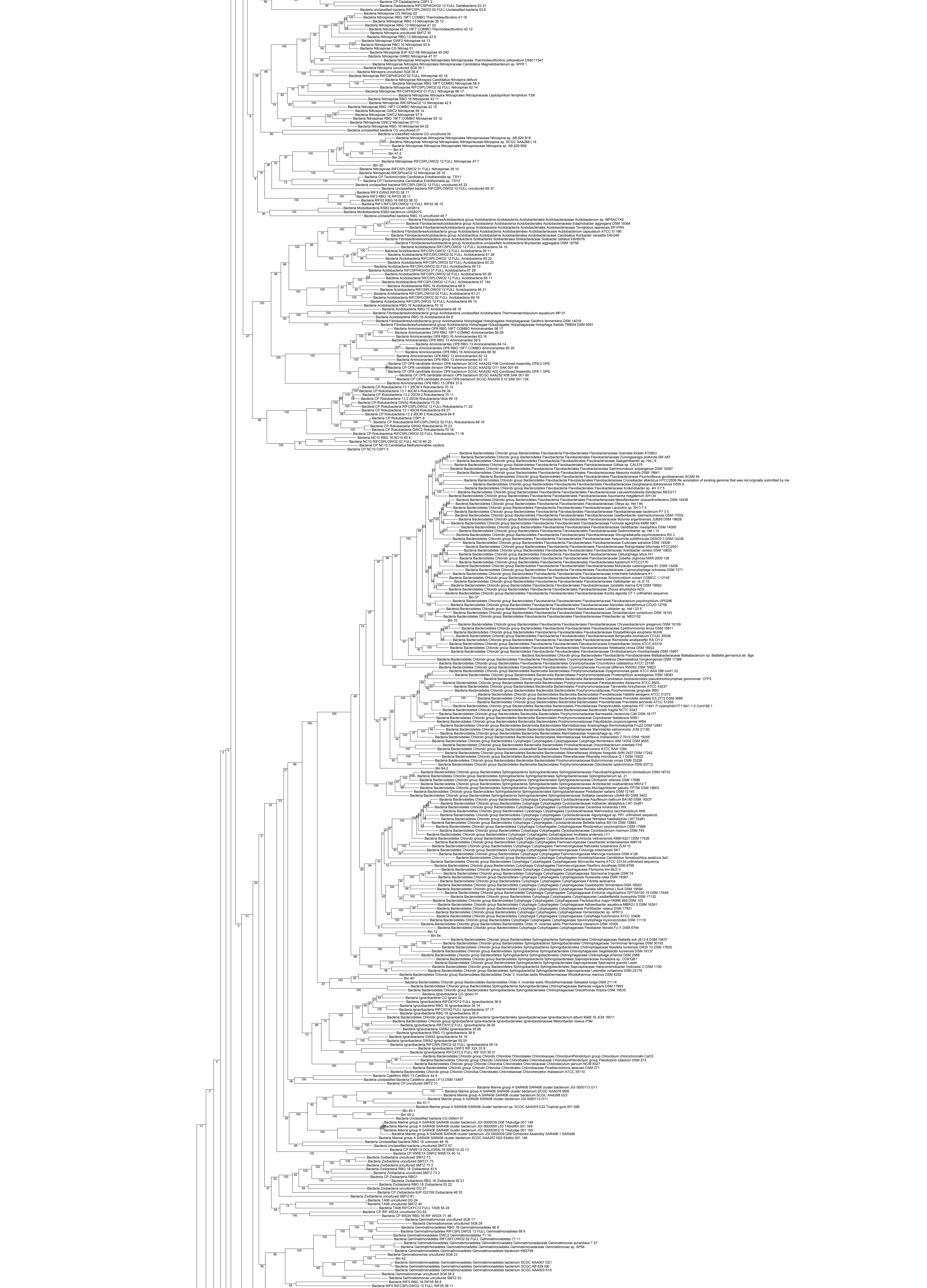

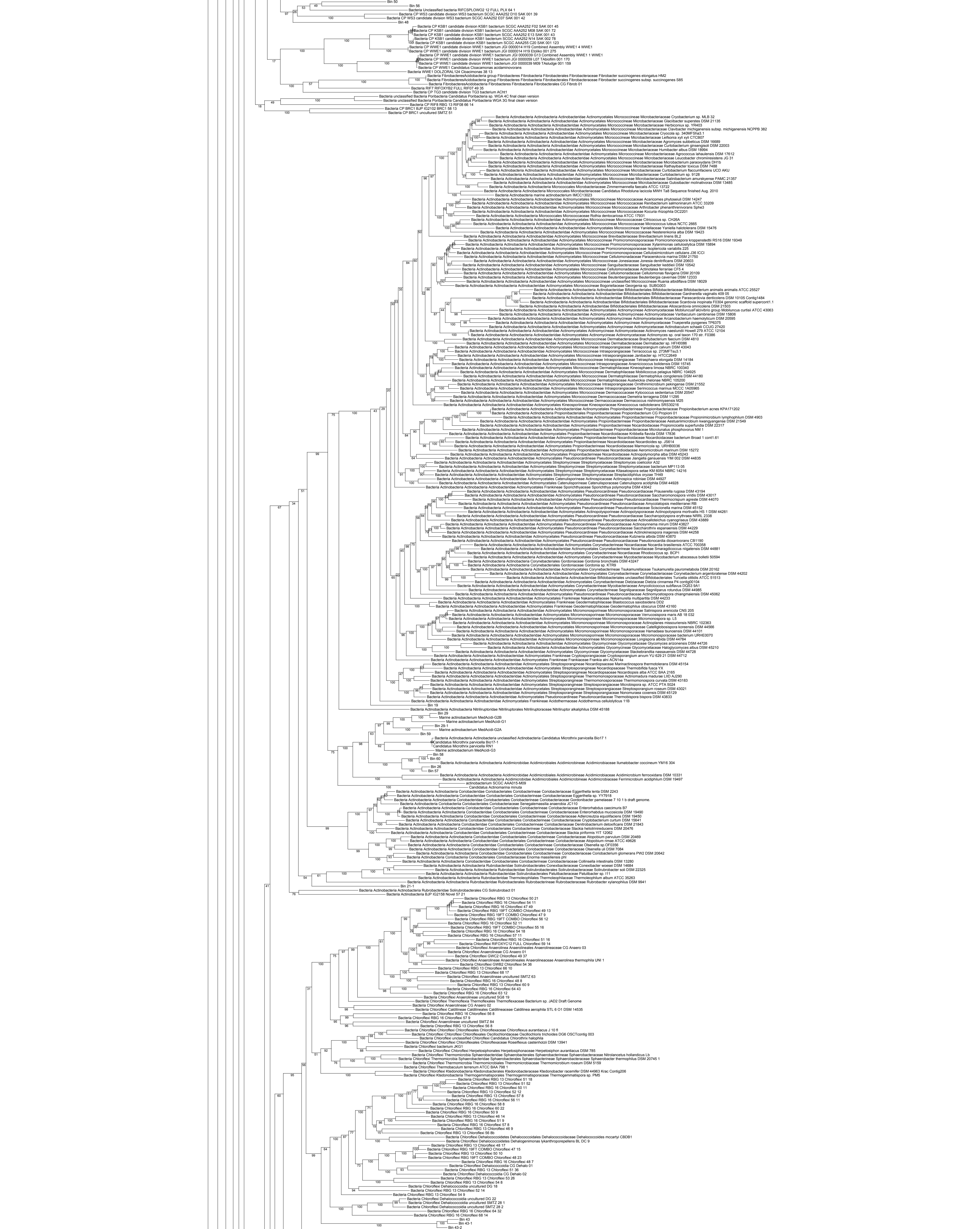

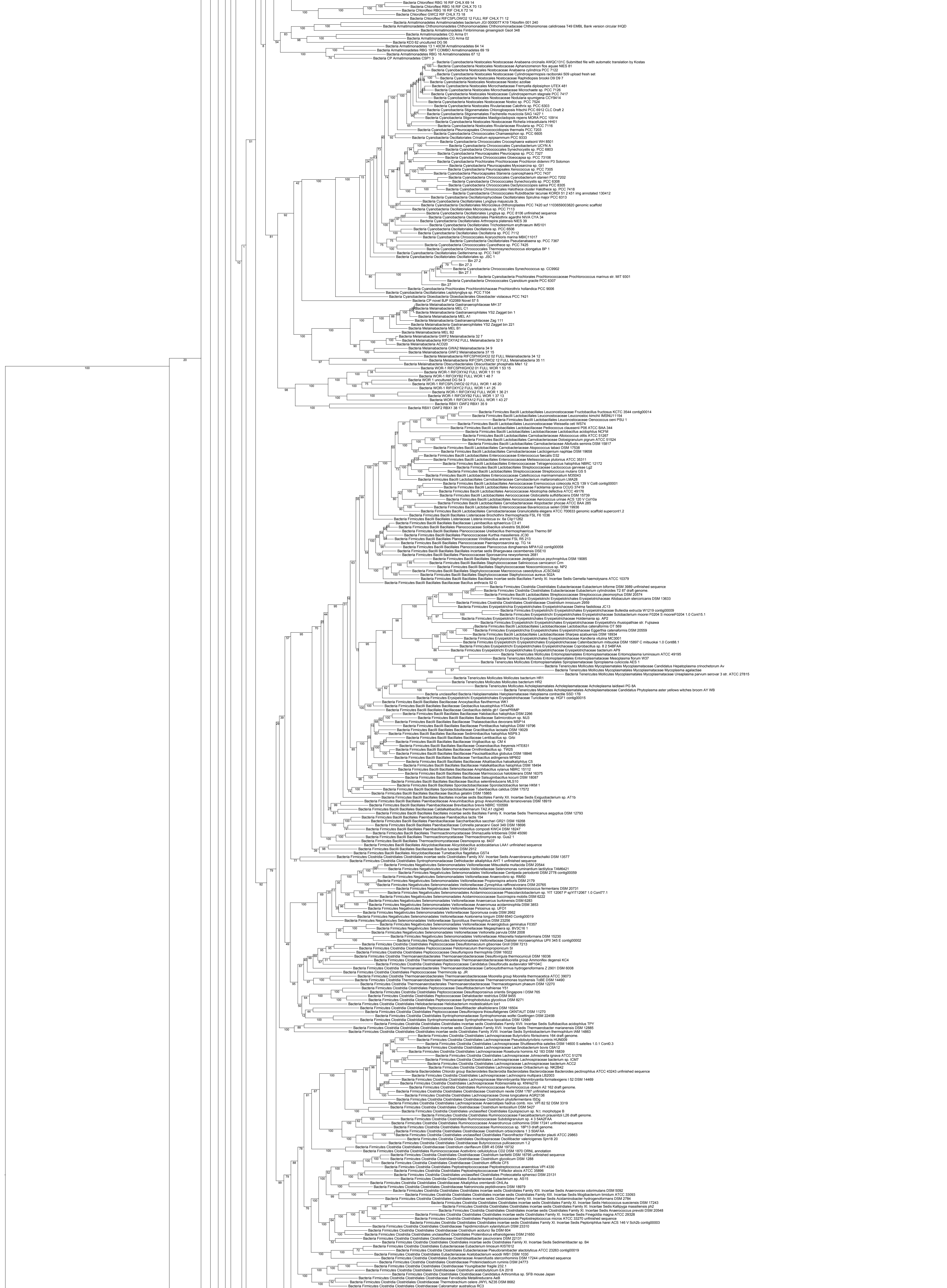

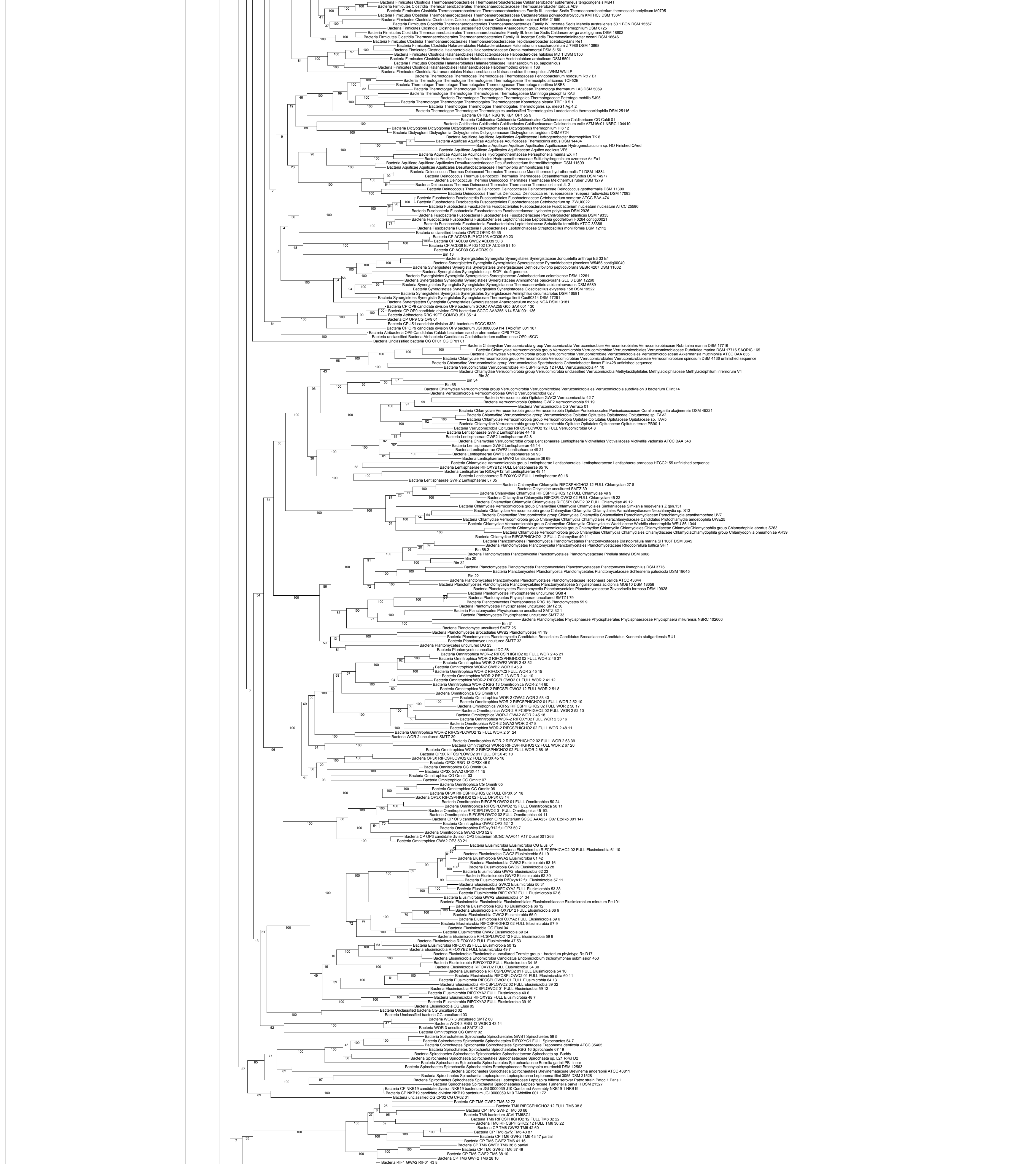

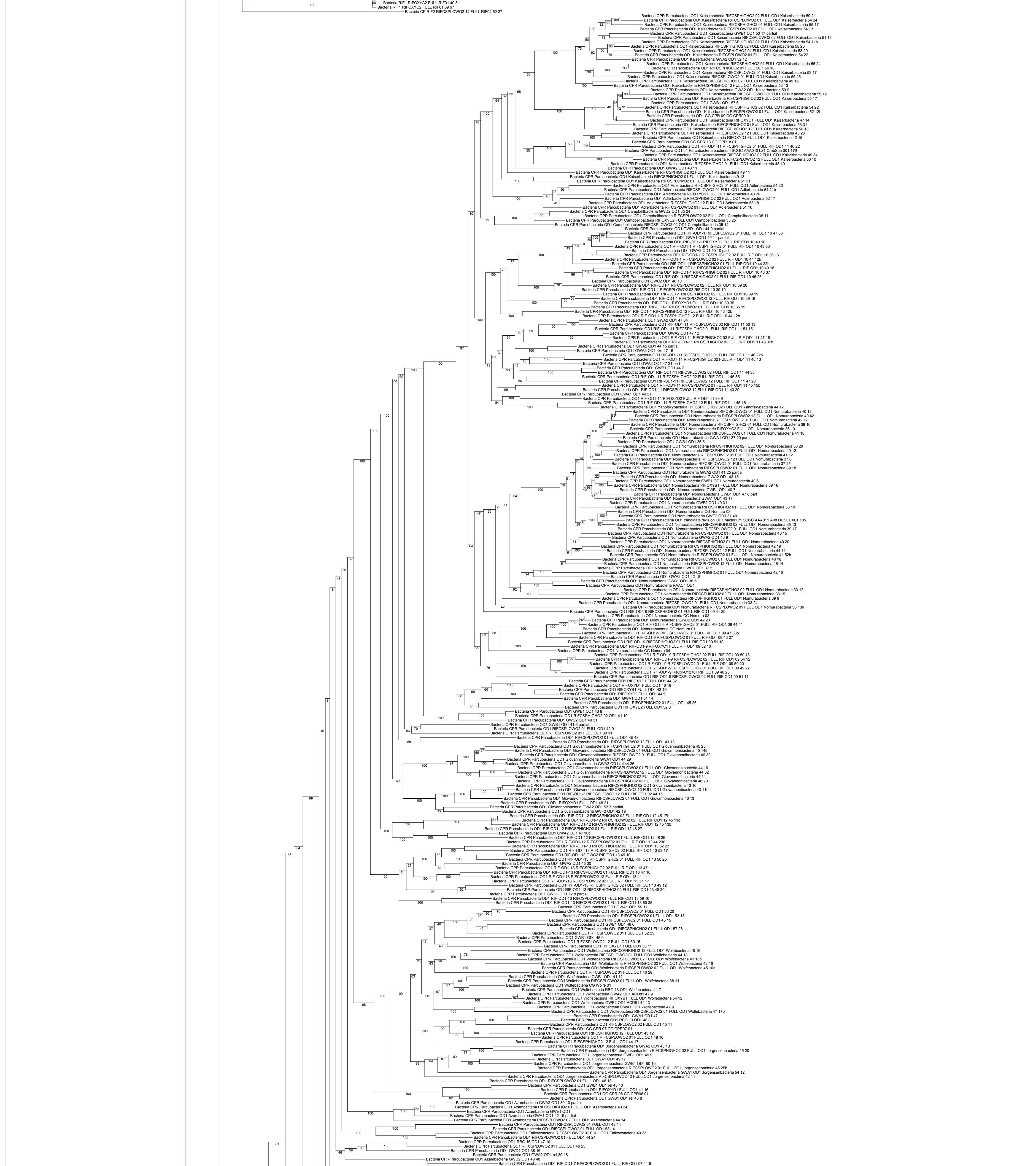

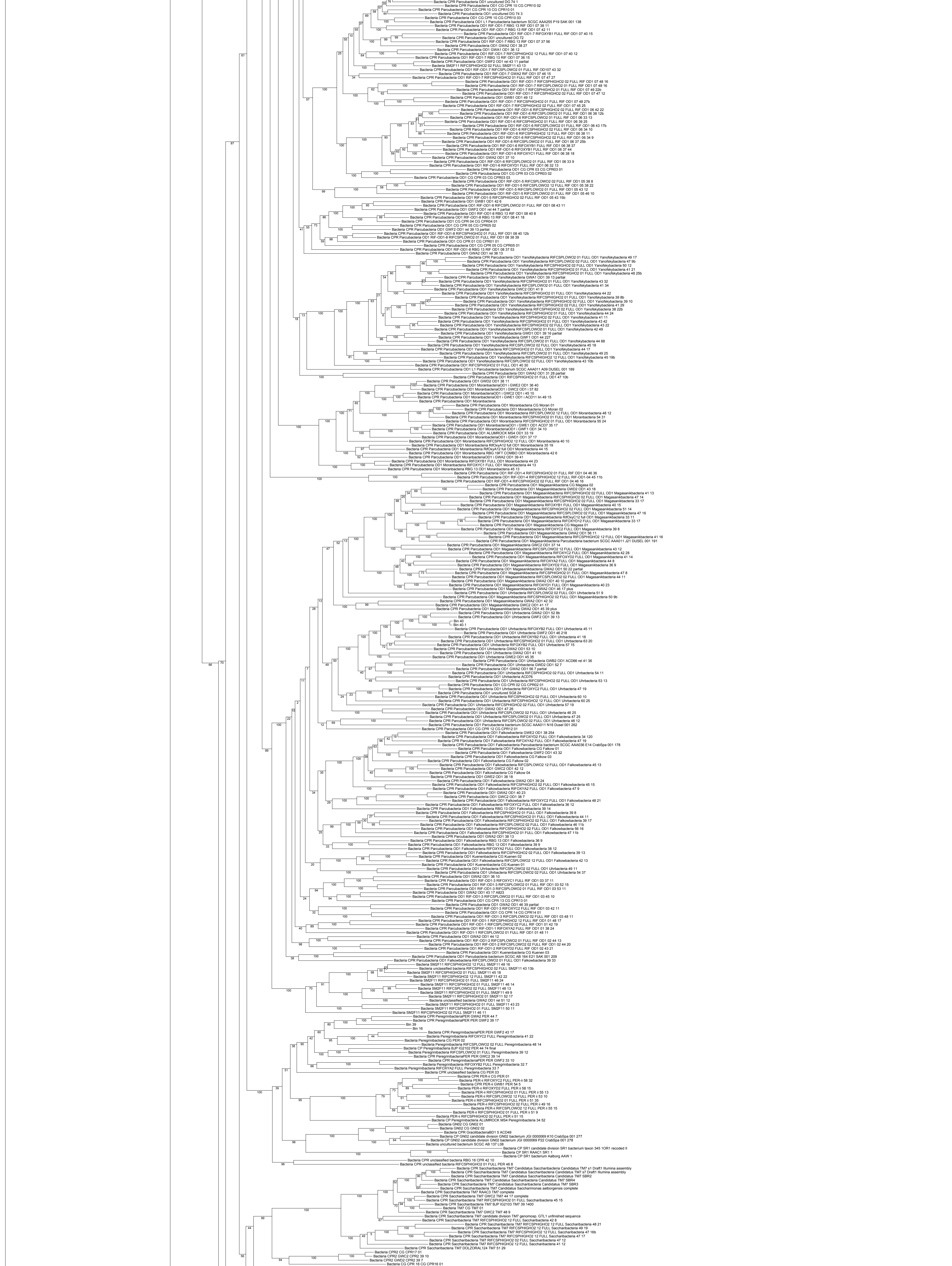

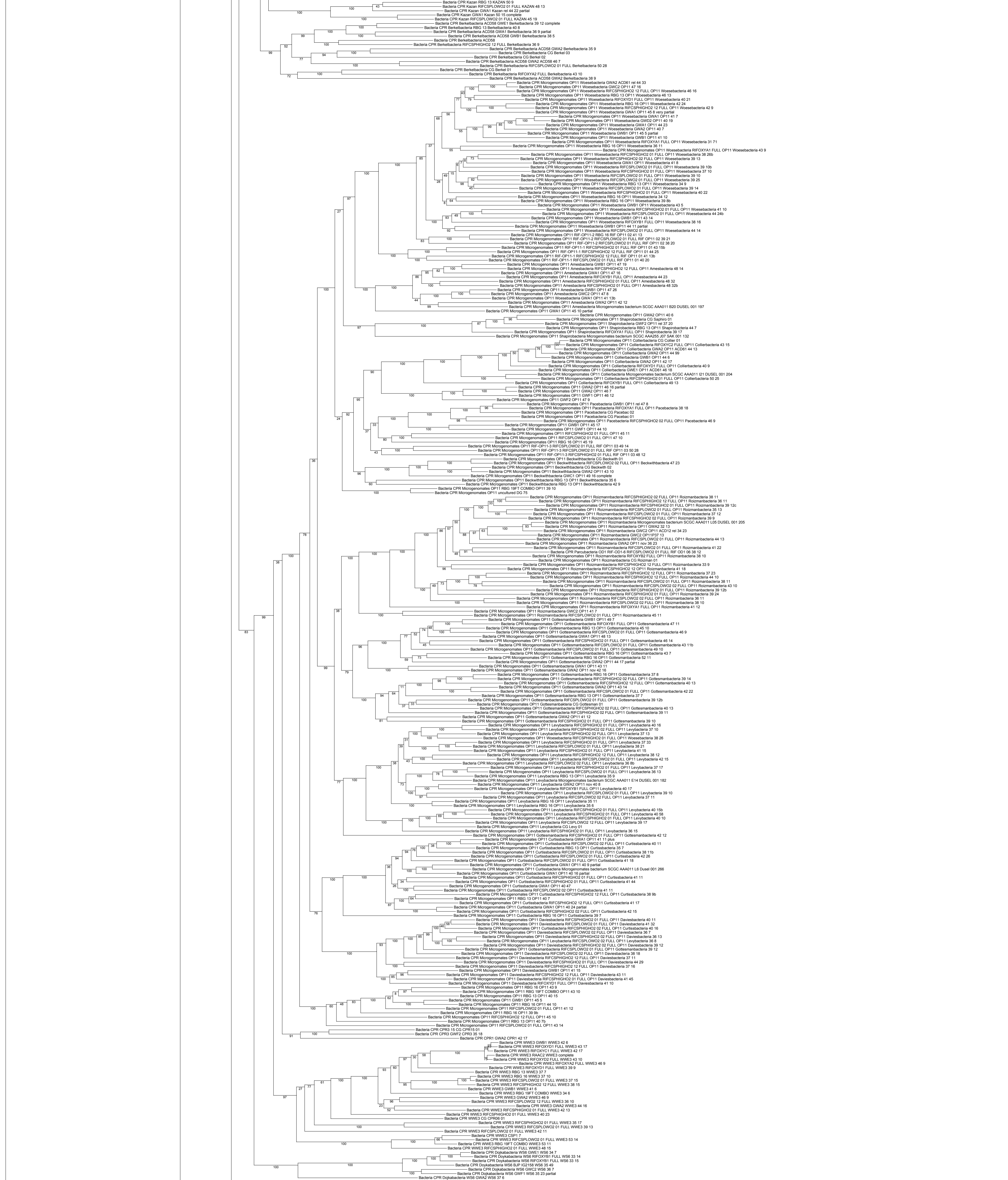

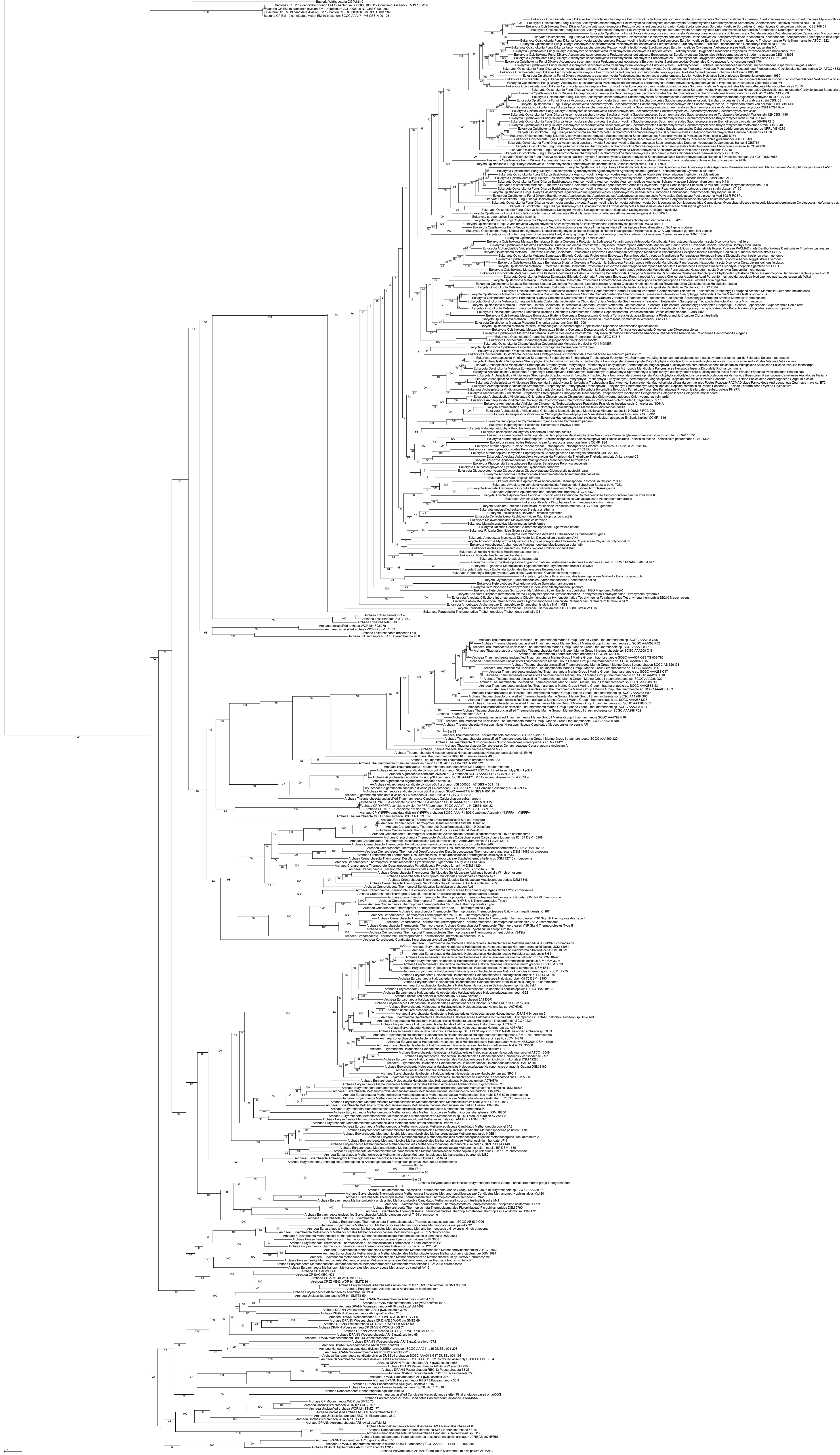

Supplement: FIG S1 [file mbo004173471sf1.pdf]
