# Supplementary material for: Metabolic Roles of Uncultivated Bacterioplankton Lineages in the Northern Gulf of Mexico “Dead Zone”
Source: mBio. 2017 Sep 12;8(5):e01017-17. doi: 10.1128/mBio.01017-17 (PMC5596340; doi:10.1128/mBio.01017-17)
Supplement: FIG S2 [file mbo004173471sf2.pdf]

Figure S2

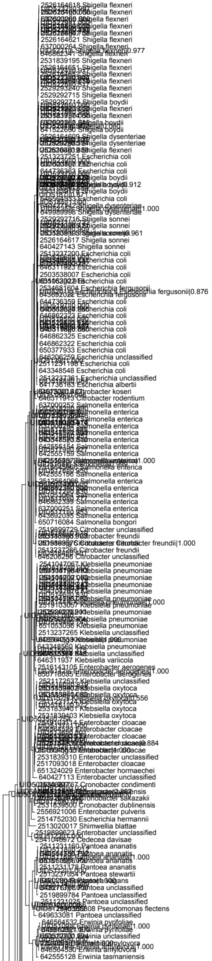
2526164618 Shigella flexneri
2526164621 Shigella flexneri
637000264 Shigella flexneri
646862341 Shigella flexneri
2531839195 Shigella flexneri
2526164651 Shigella flexneri
2529293240 Shigella flexneri
2529292715 Shigella flexneri
2529292714 Shigella boydii
641522650 Shigella boydii
2526164609 Shigella dysenteriae
2513237251 Escherichia coli
644736363 Escherichia coli
649989906 Shigella dysenteriae
2529292716 Shigella sonnei
2526164617 Shigella sonnei
640427143 Shigella sonnei
2513237200 Escherichia coli
646311923 Escherichia coli
2503538007 Escherichia coli
2534681604 Escherichia fergusonii
644736359 Escherichia coli
646862323 Escherichia coli
646862325 Escherichia coli
646862322 Escherichia coli
650377933 Escherichia coli
646206259 Escherichia unclassified
2511231198 Escherichia coli
643348548 Escherichia coli
2513237361 Escherichia unclassified
641736163 Escherichia albertii
646311913 Citrobacter rodentium
637000252 Salmonella enterica
642555154 Salmonella enterica
642555159 Salmonella enterica
642555156 Salmonella enterica
2512564066 Salmonella enterica
651053064 Salmonella enterica
646862339 Salmonella enterica
637000251 Salmonella enterica
643692095 Salmonella enterica
650716084 Salmonella bongori
2519899729 Citrobacter unclassified
2513237266 Citrobacter freundii
646206256 Citrobacter unclassified
2541047067 Klebsiella pneumoniae
2519103057 Klebsiella pneumoniae
651053086 Klebsiella pneumoniae
2513237265 Klebsiella unclassified
643348560 Klebsiella pneumoniae
646311937 Klebsiella variicola
2516143105 Enterobacter aerogenes
650716085 Enterobacter aerogenes
2521172537 Klebsiella unclassified
2531839401 Klebsiella oxytoca
2531839403 Klebsiella oxytoca
2531839310 Enterobacter unclassified
2517093018 Enterobacter cloacae
651324029 Enterobacter hormaechei
640427113 Enterobacter unclassified
2531839500 Cronobacter dublinensis
2556921006 Enterobacter pulveris
2514752030 Escherichia hermannii
2513020017 Shimwellia blattae
2519899623 Enterobacter unclassified
2541046972 Cedecea davisae
2511231160 Pantoea ananatis
2511231178 Pantoea ananatis
2513237354 Pantoea stewartii
2519899784 Pantoea unclassified
2511231025 Pantoea unclassified
649633081 Pantoea unclassified
646564532 Erwinia pyrifoliae
646564530 Erwinia amylovora
642555128 Erwinia tasmaniensis

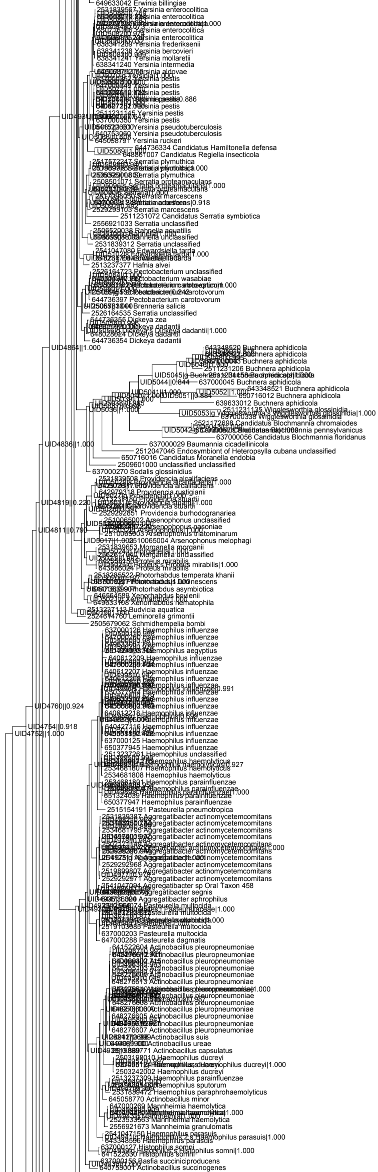

649633042 Erwinia billingiae
2531839567 Yersinia enterocolitica
638341209 Yersinia frederiksenii
638341238 Yersinia bercovieri
638341241 Yersinia mollaretii
638341240 Yersinia intermedia
2511231145 Yersinia pestis
637000350 Yersinia pestis
640753060 Yersinia pseudotuberculosis
645058791 Yersinia ruckeri
644736334 Candidatus Hamiltonella defensa
648861007 Candidatus Regiella insecticola
2517572247 Serratia plymuthica
2508501071 Serratia proteamaculans
2529293103 Serratia marcescens
2511231072 Candidatus Serratia symbiotica
2556921033 Serratia unclassified
2506520038 Rahnella aquatilis
2531839312 Serratia unclassified
2541047080 Edwardsiella tarda
2513237377 Hafnia alvei
2526164723 Pectobacterium unclassified
644736397 Pectobacterium carotovorum
2526164535 Serratia unclassified
644736355 Dickeya zea
648028024 Dickeya dadantii
644736354 Dickeya dadantii
UID4864||1.000
643348520 Buchnera aphidicola
2511231206 Buchnera aphidicola
UID5044||0.844
637000045 Buchnera aphidicola
643348521 Buchnera aphidicola
650716012 Buchnera aphidicola
639633012 Buchnera aphidicola
2511231135 Wigglesworthia glossinidia
637000338 Wigglesworthia glossinidia
2521172698 Candidatus Blochmannia chromaiodes
637000056 Candidatus Blochmannia floridanus
637000029 Baumannia cicadellinicola
2512047046 Endosymbiont of Heteropsylla cubana unclassified
650716016 Candidatus Moranella endobia
2509601000 unclassified unclassified
637000270 Sodalis glossinidius
UID4836||1.000
2531839508 Providencia alcalifaciens
642979318 Providencia rustigianii
2529292851 Providencia burhodogranariea
UID4819||0.220
2510065002 Arsenophonus unclassified
2510065003 Arsenophonus triatominarum
UID4811||0.790
2510065004 Arsenophonus melophagi
2531839653 Morganella morganii
643886024 Proteus mirabilis
2518285522 Photorhabdus temperata khanii
646564589 Xenorhabdus bovienii
649633106 Xenorhabdus nematophila
2513237113 Budvicia aquatica
2524614760 Leminorella grimontii
2505679062 Schmidhempelia bombi
637000126 Haemophilus influenzae
640612209 Haemophilus influenzae
640612207 Haemophilus influenzae
UID4760||0.924
UID4754||0.918
UID4752||1.000
637000125 Haemophilus influenzae
650377945 Haemophilus influenzae
2513237261 Haemophilus unclassified
2534681808 Haemophilus haemolyticus
651324039 Haemophilus parainfluenzae
650377947 Haemophilus parainfluenzae
2515154191 Pasteurella pneumotropica
2531839387 Aggregatibacter actinomycetemcomitans
2529292968 Aggregatibacter actinomycetemcomitans
2529292971 Aggregatibacter actinomycetemcomitans
2541047094 Aggregatibacter sp Oral Taxon 458
2519103085 Pasteurella multocida
637000203 Pasteurella multocida
647000288 Pasteurella dagmatis
641522604 Actinobacillus pleuropneumoniae
648276613 Actinobacillus pleuropneumoniae
648276608 Actinobacillus pleuropneumoniae
648276605 Actinobacillus pleuropneumoniae
648276607 Actinobacillus pleuropneumoniae
2519899771 Actinobacillus capsulatus
2503198010 Haemophilus ducreyi
2503242002 Haemophilus ducreyi
2513237309 Haemophilus parainfluenzae
2531839472 Haemophilus paraphrohaemolyticus
645058770 Actinobacillus minor
647000269 Mannheimia haemolytica
2556921673 Mannheimia granulomatis
2541047150 Haemophilus parasuis
637000127 Histophilus somni
637000156 Basfia succiniciproducens

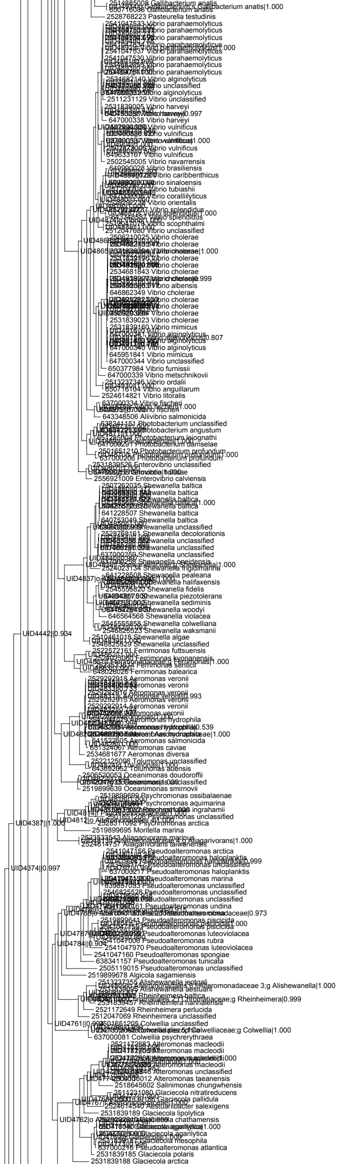
2514885008 Gallibacterium anatis
650716088 Gallibacterium anatis
2528768223 Pasteurella testudinis
2541047543 Vibrio parahaemolyticus
2541047537 Vibrio parahaemolyticus
2541047530 Vibrio parahaemolyticus
2511231129 Vibrio unclassified
2531839005 Vibrio harveyi
647000338 Vibrio harveyi
2502545005 Vibrio navarrensis
649990028 Vibrio brasiliensis
2512047080 Vibrio unclassified
2534681843 Vibrio cholerae
646862349 Vibrio cholerae
2531839023 Vibrio cholerae
2531839160 Vibrio mimicus
647000340 Vibrio alginolyticus
645951841 Vibrio mimicus
647000344 Vibrio unclassified
650377984 Vibrio furnissii
647000339 Vibrio metschnikovii
2513237346 Vibrio ordalii
650716104 Vibrio anguillarum
2524614821 Vibrio litoralis
637000334 Vibrio fischeri
643348506 Aliivibrio salmonicida
638341151 Photobacterium unclassified
651285004 Photobacterium leiognathi
647000251 Photobacterium damselae
2501651210 Photobacterium profundum
637000206 Photobacterium profundum
2531839526 Enterovibrio unclassified
2556921009 Enterovibrio calviensis
2507262035 Shewanella baltica
641228507 Shewanella baltica
640753049 Shewanella baltica
2528768161 Shewanella decolorationis
637000259 Shewanella unclassified
2524023134 Shewanella frigidimarina
641228508 Shewanella pealeana
2543555820 Shewanella fidelis
646564568 Shewanella violacea
2545555858 Shewanella colwelliana
2546825523 Shewanella waksmanii
2510461018 Shewanella algae
2546825529 Shewanella unclassified
2522572161 Ferrimonas futtsuensis
648028026 Ferrimonas balearica
2529292918 Aeromonas veronii
2529292914 Aeromonas veronii
641522605 Aeromonas salmonicida
651324007 Aeromonas caviae
2534681677 Aeromonas diversa
2522125098 Tolumonas unclassified
643692052 Tolumonas auensis
2506520053 Oceanimonas doudoroffii
2519899639 Oceanimonas smirnovii
2519899699 Psychromonas ossibalaenae
2523231092 Psychromonas arctica
2519899695 Moritella marina
2523533543 Aliagarivorans marinus
2524614757 Aliagarivorans taiwanensis
2541047156 Pseudoalteromonas arctica
637000217 Pseudoalteromonas haloplanktis
639857033 Pseudoalteromonas unclassified
2546825528 Pseudoalteromonas unclassified
2519899641 Pseudoalteromonas piscicida
2541047006 Pseudoalteromonas rubra
2541047970 Pseudoalteromonas luteoviolacea
2541047160 Pseudoalteromonas spongiae
638341157 Pseudoalteromonas tunicata
2505119015 Pseudoalteromonas unclassified
2519899678 Algicola sagamiensis
2513237355 Alishewanella jeotgali
2521172649 Rheinheimera perlucida
2512047069 Rheinheimera unclassified
2501651205 Colwellia unclassified
637000081 Colwellia psychrerythraea
2521172683 Alteromonas macleodii
2518645602 Salinimonas chungwhensis
2511231080 Glaciecola nitratireducens
2524614540 Aestuariibacter salexigens
2531839189 Glaciecola lipolytica
637000216 Pseudoalteromonas atlantica
2531839185 Glaciecola polaris
2531839188 Glaciecola arctica
UID4442||0.934
UID4387||1.000
UID4374||0.997
UID4784||0.904
UID4761||0.992

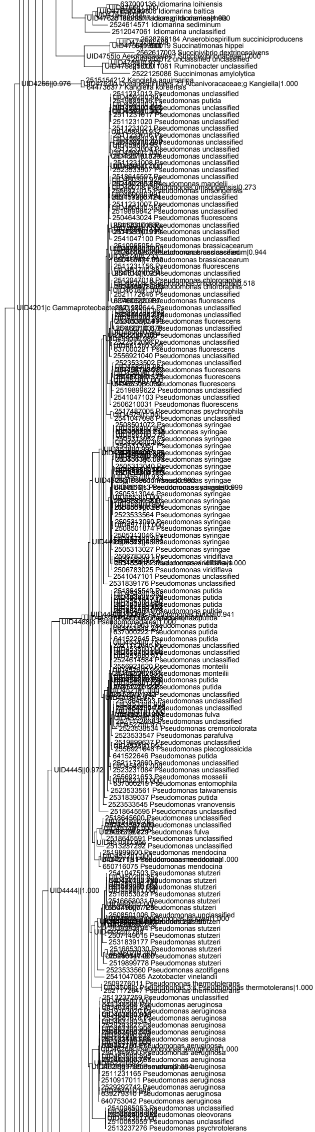

637000136 Idiomarina loihiensis
2524614571 Idiomarina sediminum
2512047061 Idiomarina unclassified
2528768184 Anaerobiospirillum succiniciproducens
2562617003 Succinivibrio dextrinosolvens
2522125086 Succinimonas amylolytica
UID4266||0.976
2515154212 Kangiella aquimarina
644736377 Kangiella koreensis
2511231012 Pseudomonas unclassified
2511231017 Pseudomonas unclassified
2511231020 Pseudomonas unclassified
2511231021 Pseudomonas unclassified
2523533507 Pseudomonas unclassified
2511231007 Pseudomonas unclassified
2519899642 Pseudomonas unclassified
2504643024 Pseudomonas fluorescens
2541047100 Pseudomonas unclassified
2510096505 Pseudomonas brassicacearum
2511231156 Pseudomonas fluorescens
2512047018 Pseudomonas chlororaphis
2521172646 Pseudomonas unclassified
2556921040 Pseudomonas unclassified
2523533502 Pseudomonas unclassified
637000221 Pseudomonas fluorescens
2519899622 Pseudomonas unclassified
2541047103 Pseudomonas unclassified
2506210031 Pseudomonas fluorescens
2517487005 Pseudomonas psychrophila
2541047098 Pseudomonas unclassified
2508501072 Pseudomonas syringae
2523533564 Pseudomonas syringae
2505313044 Pseudomonas syringae
2505313060 Pseudomonas syringae
2508501074 Pseudomonas syringae
2505313046 Pseudomonas syringae
2505313027 Pseudomonas syringae
2506783031 Pseudomonas viridiflava
2506783025 Pseudomonas viridiflava
2541047101 Pseudomonas unclassified
2531839176 Pseudomonas unclassified
2518645549 Pseudomonas putida
637000222 Pseudomonas putida
641522645 Pseudomonas putida
2524614584 Pseudomonas unclassified
2556921620 Pseudomonas monteilii
2523533534 Pseudomonas cremoricolorata
2523533547 Pseudomonas parafulva
2519899637 Pseudomonas unclassified
2556921648 Pseudomonas plecoglossicida
641522646 Pseudomonas putida
2521172660 Pseudomonas unclassified
2523231084 Pseudomonas unclassified
2556921653 Pseudomonas mosselii
637000219 Pseudomonas entomophila
2523533561 Pseudomonas taiwanensis
2531839037 Pseudomonas putida
2523533545 Pseudomonas vranovensis
2518645595 Pseudomonas unclassified
UID4445||0.972
2518645600 Pseudomonas unclassified
2518645591 Pseudomonas unclassified
2513237292 Pseudomonas unclassified
2519899600 Pseudomonas mendocina
650716075 Pseudomonas mendocina
UID4444||1.000
2541047503 Pseudomonas stutzeri
2516653029 Pseudomonas stutzeri
2516653031 Pseudomonas stutzeri
2508501006 Pseudomonas unclassified
2507149015 Pseudomonas stutzeri
2531839177 Pseudomonas stutzeri
2516653030 Pseudomonas stutzeri
2519899778 Pseudomonas stutzeri
2523533560 Pseudomonas azotifigens
2541047085 Azotobacter vinelandii
2509276011 Pseudomonas thermotolerans
2513237259 Pseudomonas unclassified
2511231165 Pseudomonas aeruginosa
2510917011 Pseudomonas aeruginosa
2529292742 Pseudomonas aeruginosa
640753042 Pseudomonas aeruginosa
2510065053 Pseudomonas unclassified
2510065055 Pseudomonas unclassified
2513237276 Pseudomonas psychrotolerans

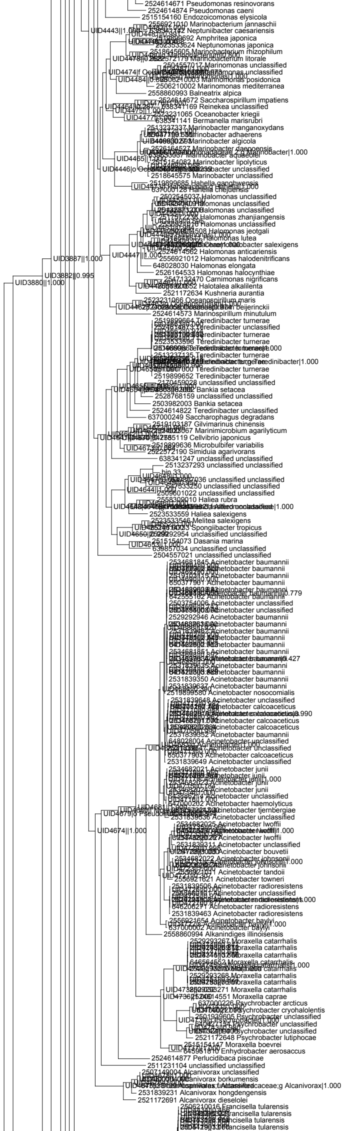
2524614671 Pseudomonas resinovorans
2524614874 Pseudomonas caeni
2515154160 Endozoicomonas elysicola
2556921010 Marinobacterium jannaschii
2519899692 Amphritea japonica
2523533624 Neptunomonas japonica
2518645605 Marinobacterium rhizophilum
2504557017 Marinomonas unclassified
2506210003 Marinomonas posidonica
2506210002 Marinomonas mediterranea
2558860993 Balneatrix alpica
2524614672 Saccharospirillum impatiens
638341169 Reinekea unclassified
2523231065 Oceanobacter kriegii
638341141 Bermanella marisrubri
2513237337 Marinobacter manganoxydans
2526164527 Marinobacter daepoensis
2515154082 Marinobacter lipolyticus
2518645575 Marinobacter unclassified
2519899685 Hahella ganghwensis
2502545037 Halomonas unclassified
2517572230 Halomonas zhanjiangensis
2524614562 Halomonas anticariensis
2556921012 Halomonas halodenitrificans
648028030 Halomonas elongata
2526164533 Halomonas halocynthiae
2547132470 Carnimonas nigrificans
2521172634 Kushneria aurantia
2523231066 Oceanospirillum maris
2524614573 Marinospirillum minutulum
2519899664 Teredinibacter turnerae
2513237135 Teredinibacter turnerae
2519899652 Teredinibacter turnerae
2170459028 unclassified unclassified
2528768159 unclassified unclassified
2503982003 Bankia setacea
2524614822 Teredinibacter unclassified
637000249 Saccharophagus degradans
2519103187 Gilvimarinus chinensis
2519899636 Microbulbifer variabilis
2522572190 Simiduia agarivorans
638341247 unclassified unclassified
2513237293 unclassified unclassified
2509601022 unclassified unclassified
2558309010 Haliea rubra
2523533559 Haliea salexigens
2523533546 Melitea salexigens
2515154073 Dasania marina
639857034 unclassified unclassified
2504557021 unclassified unclassified
2534681845 Acinetobacter baumannii
650377901 Acinetobacter baumannii
642555102 Acinetobacter baumannii
2503754006 Acinetobacter unclassified
2529292946 Acinetobacter baumannii
2531839350 Acinetobacter baumannii
2519899580 Acinetobacter nosocomialis
2531839648 Acinetobacter unclassified
2531839552 Acinetobacter baumannii
648028004 Acinetobacter unclassified
650377903 Acinetobacter calcoaceticus
2531839649 Acinetobacter unclassified
2534682021 Acinetobacter junii
2534682024 Acinetobacter junii
647000202 Acinetobacter haemolyticus
2531839536 Acinetobacter unclassified
2534682025 Acinetobacter lwoffii
2531839311 Acinetobacter unclassified
2556921011 Acinetobacter tandoii
2556921621 Acinetobacter towneri
2531839506 Acinetobacter radioresistens
646206271 Acinetobacter radioresistens
2531839463 Acinetobacter radioresistens
2556921654 Acinetobacter baylyi
637000002 Acinetobacter baylyi
2558860994 Alkanindiges illinoisensis
2529293267 Moraxella catarrhalis
2529293268 Moraxella catarrhalis
2524614551 Moraxella caprae
637000226 Psychrobacter arcticus
2501939605 Psychrobacter unclassified
2521172648 Psychrobacter lutiphocae
2515154147 Moraxella boevrei
2524614877 Perlucidibaca piscinae
2511231104 unclassified unclassified
2507149004 Alcanivorax unclassified
2531839231 Alcanivorax hongdengensis
2521172691 Alcanivorax dieselolei
2506210016 Francisella tularensis
UID3887||1.000
UID3882||0.995
UID3880||1.000
UID4674||1.000

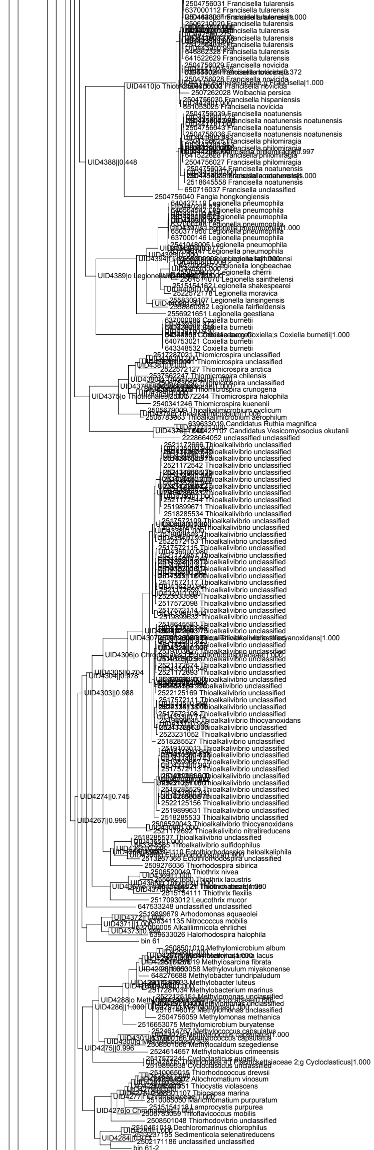
2504756031 Francisella tularensis
637000112 Francisella tularensis
2506210020 Francisella tularensis
646862328 Francisella tularensis
641522629 Francisella tularensis
2504756029 Francisella novicida
2507262028 Wolbachia persica
2504756030 Francisella hispaniensis
651053025 Francisella novicida
2504756039 Francisella noatunensis
2504756043 Francisella noatunensis
2504756036 Francisella noatunensis noatunensis
641522628 Francisella philomiragia
2504756027 Francisella philomiragia
2504756034 Francisella noatunensis
2518645558 Francisella noatunensis
650716037 Francisella unclassified
UID4388||0.448
2504756040 Fangia hongkongiensis
640427119 Legionella pneumophila
650377956 Legionella pneumophila
637000146 Legionella pneumophila
2541948005 Legionella pneumophila
2561511070 Legionella sainthelensi
2515154162 Legionella shakespearei
2522572178 Legionella moravica
2558309107 Legionella lansingensis
2558860982 Legionella fairfieldensis
2556921651 Legionella geestiana
637000086 Coxiella burnetii
640753021 Coxiella burnetii
643348532 Coxiella burnetii
2517287021 Thiomicrospira unclassified
2522572127 Thiomicrospira arctica
2537562247 Thiomicrospira chilensis
2540341246 Thiomicrospira kuenenii
2505679009 Thioalkalimicrobium cyclicum
2506783063 Thioalkalimicrobium aerophilum
639633019 Candidatus Ruthia magnifica
2228664052 unclassified unclassified
2521172666 Thioalkalivibrio unclassified
2521172542 Thioalkalivibrio unclassified
2521172544 Thioalkalivibrio unclassified
2519899671 Thioalkalivibrio unclassified
2518285534 Thioalkalivibrio unclassified
2517572109 Thioalkalivibrio unclassified
2522572153 Thioalkalivibrio unclassified
2517572115 Thioalkalivibrio unclassified
2517572117 Thioalkalivibrio unclassified
2523533598 Thioalkalivibrio unclassified
2517572098 Thioalkalivibrio unclassified
2517572114 Thioalkalivibrio unclassified
2519899632 Thioalkalivibrio unclassified
2518645583 Thioalkalivibrio unclassified
2521172674 Thioalkalivibrio unclassified
2521172693 Thioalkalivibrio unclassified
2522125169 Thioalkalivibrio unclassified
2517572111 Thioalkalivibrio unclassified
2517572108 Thioalkalivibrio unclassified
2523231052 Thioalkalivibrio unclassified
2518285527 Thioalkalivibrio unclassified
UID4305||0.704
UID4304||0.978
UID4303||0.988
2519103013 Thioalkalivibrio unclassified
2517572113 Thioalkalivibrio unclassified
2518285529 Thioalkalivibrio unclassified
2522125156 Thioalkalivibrio unclassified
2519899631 Thioalkalivibrio unclassified
2518285533 Thioalkalivibrio unclassified
UID4274||0.745
UID4267||0.996
2506520043 Thioalkalivibrio thiocyanoxidans
2521172692 Thioalkalivibrio nitratireducens
2518285537 Thioalkalivibrio unclassified
2509276036 Thiorhodospira sibirica
2506520049 Thiothrix nivea
2556921650 Thiothrix lacustris
2515154111 Thiothrix flexilis
2517093012 Leucothrix mucor
647533248 unclassified unclassified
2519899679 Arhodomonas aquaeolei
638341135 Nitrococcus mobilis
637000005 Alkalilimnicola ehrlichei
639633026 Halorhodospira halophila
bin 61
2508501010 Methylomicrobium album
648276688 Methylobacter tundripaludum
2517287034 Methylobacterium marinus
UID4286||1.000
2504756059 Methylomonas methanica
2516653075 Methylomicrobium buryatense
2524614767 Methylococcus capsulatus
2508501066 Methylocaldum szegediense
2524614657 Methylohalobius crimeensis
UID4275||0.996
2517572241 Cycloclasticus pugetii
2510065015 Thiorhodococcus drewsii
2508501107 Thiocapsa marina
2510065050 Marichromatium purpuratum
2515154118 Lamprocystis purpurea
2506783059 Thioflavicoccus mobilis
2508501048 Thiorhodovibrio unclassified
2510461019 Dechloromarinus chlorophilus
2513237155 Sedimenticola selenatireducens
2502171186 unclassified unclassified
bin 61-2

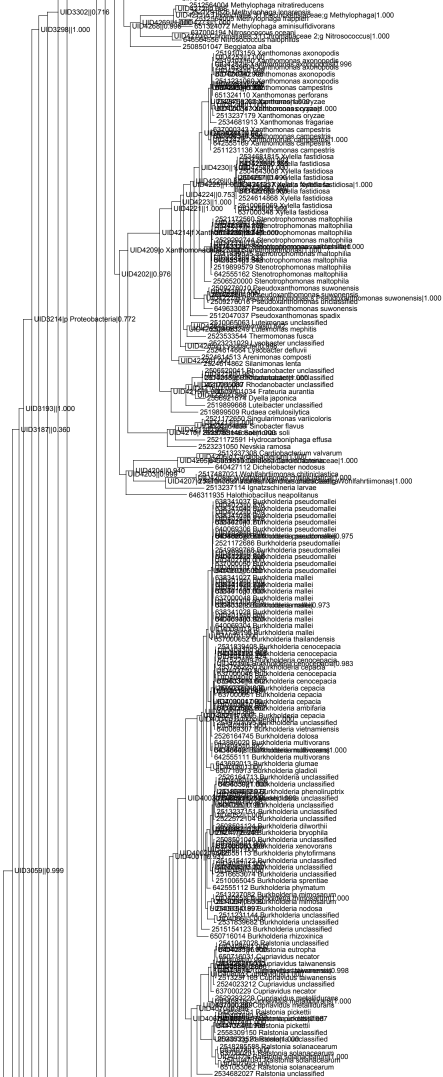

UID3302||0.716
UID3298||1.000
UID3214|p Proteobacteria|0.772
UID3193||1.000
UID3187||0.360
UID3059||0.999
2512564004 Methylophaga nitratireducens
Methylophaga lonarensis
Piscirickettsiaceae;g Methylophaga|1.000
Methylophaga frappieri
UID4269||1.000
UID4268||0.996
651324072 Methylophaga aminisulfidivorans
637000194 Nitrosococcus oceani
Chromatiales 3;f Chromatiaceae 2;g Nitrosococcus|1.000
646564556 Nitrosococcus halophilus
2508501047 Beggiatoa alba
2519103159 Xanthomonas axonopodis
Xanthomonas axonopodis
Xanthomonas axonopodis|0.996
Xanthomonas axonopodis
Xanthomonas axonopodis
2511231060 Xanthomonas axonopodis
Xanthomonas campestris
651324110 Xanthomonas perforans
Xanthomonas oryzae
Xanthomonas oryzae|1.000
2513237179 Xanthomonas oryzae
2534681913 Xanthomonas fragariae
637000343 Xanthomonas campestris
Xanthomonas campestris
Xanthomonas campestris|1.000
Xanthomonas campestris
2511231136 Xanthomonas campestris
2534681815 Xylella fastidiosa
Xylella fastidiosa
2504643008 Xylella fastidiosa
Xylella fastidiosa
Xylella fastidiosa|1.000
Xylella fastidiosa
2524614868 Xylella fastidiosa
2510065069 Xylella fastidiosa
637000348 Xylella fastidiosa
UID4230||1.000
UID4226||0.585
UID4225||1.000
UID4224||0.753
UID4223||1.000
UID4221||1.000
UID4214|f Xanthomonadaceae
UID4209|o Xanthomonadales
UID4202||0.976
2521172560 Stenotrophomonas maltophilia
Stenotrophomonas maltophilia
Stenotrophomonas maltophilia
2529292744 Stenotrophomonas maltophilia
Stenotrophomonas maltophilia|1.000
Stenotrophomonas maltophilia
Stenotrophomonas maltophilia
2519899579 Stenotrophomonas maltophilia
642555162 Stenotrophomonas maltophilia
2506520000 Stenotrophomonas maltophilia
2509276010 Pseudoxanthomonas suwonensis
Pseudoxanthomonas suwonensis
Pseudoxanthomonas suwonensis|1.000
2509276016 Pseudoxanthomonas unclassified
649633087 Pseudoxanthomonas suwonensis
2512047037 Pseudoxanthomonas spadix
2510065063 Luteimonas unclassified
Luteimonas mephitis
2523533544 Thermomonas fusca
2523231029 Lysobacter unclassified
2524614664 Lysobacter defluvii
2524614513 Arenimonas composti
2524614862 Silanimonas lenta
2506520041 Rhodanobacter unclassified
Rhodanobacter|1.000
Rhodanobacter unclassified
Rhodanobacter unclassified
2509601034 Frateuria aurantia
2556921674 Dyella japonica
2519899668 Luteibacter unclassified
2519899509 Rudaea cellulosilytica
2521172650 Singularimonas variicoloris
Sinobacter flavus
Steroidobacter soli
2521172591 Hydrocarboniphaga effusa
2523231050 Nevskia ramosa
2513237308 Cardiobacterium valvarum
Cardiobacteriaceae|1.000
640427112 Dichelobacter nodosus
UID4204||0.940
UID4203||0.999
2517487021 Wohlfahrtiimonas chitiniclastica
Wohlfahrtiimonas chitiniclastica|1.000
Wohlfahrtiimonas|1.000
2513237114 Ignatzschineria larvae
646311935 Halothiobacillus neapolitanus
638341037 Burkholderia pseudomallei
Burkholderia pseudomallei
Burkholderia pseudomallei
Burkholderia pseudomallei
640069306 Burkholderia pseudomallei
Burkholderia pseudomallei|0.975
2521172686 Burkholderia pseudomallei
2519899768 Burkholderia pseudomallei
Burkholderia pseudomallei
637000050 Burkholderia pseudomallei
Burkholderia pseudomallei
638341027 Burkholderia mallei
Burkholderia mallei
Burkholderia mallei
Burkholderia mallei
637000048 Burkholderia mallei
Burkholderia mallei|0.973
638341028 Burkholderia mallei
Burkholderia mallei
640069304 Burkholderia mallei
Burkholderia mallei
637000052 Burkholderia thailandensis
2531839408 Burkholderia cenocepacia
Burkholderia cenocepacia
Burkholderia cenocepacia
Burkholderia cenocepacia|0.983
Burkholderia cepacia
637000046 Burkholderia cenocepacia
Burkholderia cenocepacia
Burkholderia cepacia
637000051 Burkholderia cepacia
Burkholderia cepacia
Burkholderia ambifaria
Burkholderia cepacia
Burkholderia unclassified
640069307 Burkholderia vietnamiensis
2526164745 Burkholderia dolosa
643886020 Burkholderia multivorans
Burkholderia multivorans|1.000
642555111 Burkholderia multivorans
643692013 Burkholderia glumae
650716013 Burkholderia gladioli
2526164713 Burkholderia unclassified
Burkholderia unclassified
Burkholderia phenoliruptrix
Burkholderia unclassified
Burkholderia unclassified
2513237151 Burkholderia unclassified
2522572104 Burkholderia unclassified
2508501124 Burkholderia dilworthii
Burkholderia bryophila
2508501040 Burkholderia unclassified
Burkholderia xenovorans
Burkholderia phytofirmans
2515154122 Burkholderia unclassified
Burkholderia unclassified
2516653074 Burkholderia unclassified
2510065045 Burkholderia sprentiae
642555112 Burkholderia phymatum
2513237082 Burkholderia mimosarum
Burkholderia mimosarum|1.000
Burkholderia mimosarum
Burkholderia nodosa
2511231144 Burkholderia unclassified
2531839682 Burkholderia unclassified
2515154123 Burkholderia unclassified
650716014 Burkholderia rhizoxinica
UID4001||0.931
2541047028 Ralstonia unclassified
Ralstonia eutropha
650716031 Cupriavidus necator
Cupriavidus taiwanensis
Cupriavidus taiwanensis|0.998
2513237165 Cupriavidus taiwanensis
2524023212 Cupriavidus unclassified
637000229 Cupriavidus necator
2529293220 Cupriavidus metallidurans
Cupriavidus metallidurans|1.000
Cupriavidus metallidurans
Ralstonia pickettii
Ralstonia pickettii|0.987
Ralstonia pickettii
2558309150 Ralstonia unclassified
Ralstonia|1.000
Ralstonia unclassified
2518285588 Ralstonia solanacearum
Ralstonia solanacearum
Ralstonia solanacearum|1.000
Ralstonia solanacearum
651053062 Ralstonia solanacearum
2534682027 Ralstonia unclassified

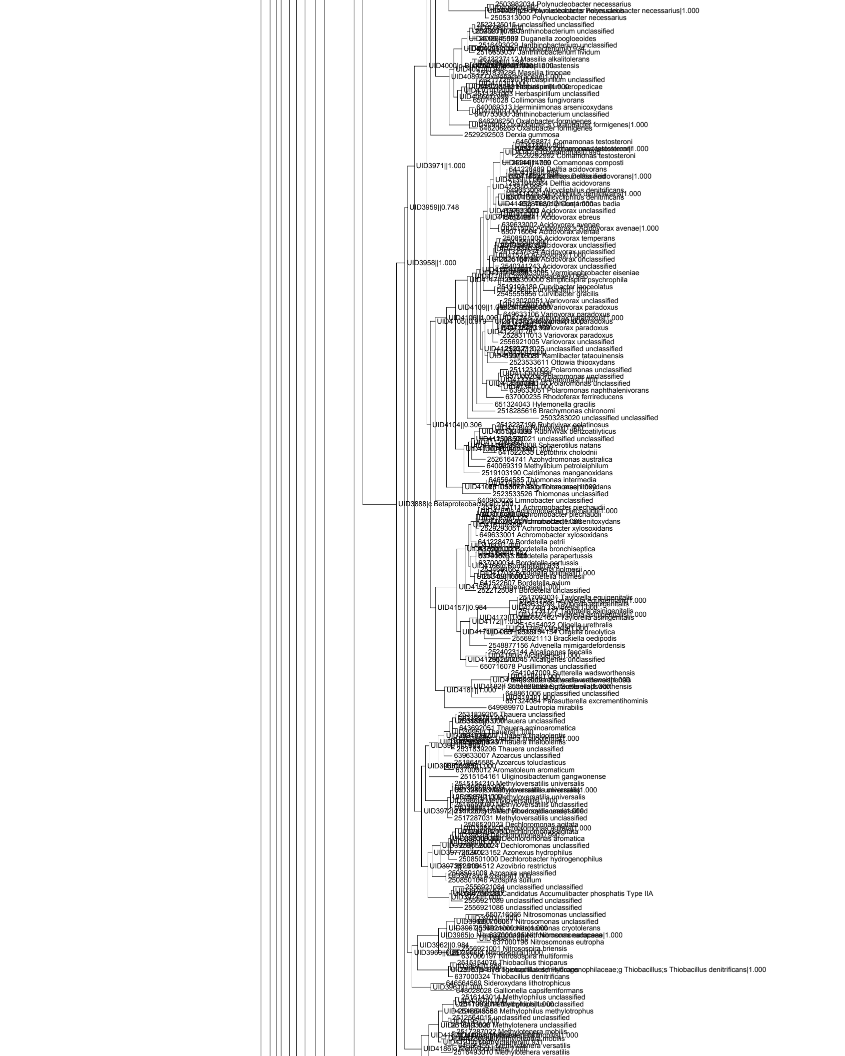
2503982034 Polynucleobacter necessarius
2505313000 Polynucleobacter necessarius
2522125015 unclassified unclassified
2516493029 Janthinobacterium unclassified
2516653037 Janthinobacterium lividum
2513237112 Massilia alkalitolerans
2531839286 Massilia timonae
650716028 Collimonas fungivorans
640069313 Herminiimonas arsenicoxydans
640753030 Janthinobacterium unclassified
646206250 Oxalobacter formigenes
646206265 Oxalobacter formigenes
2529292503 Derxia gummosa
645058871 Comamonas testosteroni
2529292992 Comamonas testosteroni
641228489 Delftia acidovorans
UID3971||1.000
UID3959||0.748
639633002 Acidovorax avenae
650716004 Acidovorax avenae
2508501005 Acidovorax temperans
2540341243 Acidovorax unclassified
UID3958||1.000
2519103180 Curvibacter lanceolatus
2545555856 Curvibacter gracilis
2513020051 Variovorax unclassified
649633106 Variovorax paradoxus
2528311013 Variovorax paradoxus
2556921005 Variovorax unclassified
2523533611 Ottowia thiooxydans
2511231002 Polaromonas unclassified
639633051 Polaromonas naphthalenivorans
637000235 Rhodoferax ferrireducens
651324043 Hylemonella gracilis
2518285616 Brachymonas chironomi
2503283020 unclassified unclassified
UID4104||0.306
2513237199 Rubrivivax gelatinosus
641522633 Leptothrix cholodnii
2526164741 Azohydromonas australica
640069319 Methylibium petroleiphilum
2519103190 Caldimonas manganoxidans
646564585 Thiomonas intermedia
2523533526 Thiomonas unclassified
UID3888|c Betaproteobacteria|1.000
640963026 Limnobacter unclassified
2529293051 Achromobacter xylosoxidans
649633001 Achromobacter xylosoxidans
641228479 Bordetella petrii
641522607 Bordetella avium
2522125081 Bordetella unclassified
UID4157||0.984
2517093031 Taylorella equigenitalis
2515154022 Oligella urethralis
2556921113 Brackiella oedipodis
2548877156 Advenella mimigardefordensis
2524023144 Alcaligenes faecalis
650716078 Pusillimonas unclassified
2541047009 Sutterella wadsworthensis
UID4181||1.000
648861006 unclassified unclassified
651324084 Parasutterella excrementihominis
649989970 Lautropia mirabilis
2531839205 Thauera unclassified
643692051 Thauera aminoaromatica
2531839206 Thauera unclassified
639633007 Azoarcus unclassified
2518645585 Azoarcus toluclasticus
637000012 Aromatoleum aromaticum
2515154161 Uliginosibacterium gangwonense
2515154210 Methyloversatilis universalis
2517287031 Methyloversatilis unclassified
2506520023 Dechloromonas agitata
2508501000 Dechlorobacter hydrogenophilus
2508501008 Azospira unclassified
2508501046 Azospira suillum
2556921084 unclassified unclassified
2556921089 unclassified unclassified
2556921086 unclassified unclassified
650716066 Nitrosomonas unclassified
637000196 Nitrosomonas eutropha
UID3962||0.984
2556921001 Nitrosospira briensis
637000197 Nitrosospira multiformis
2515154076 Thiobacillus thioparus
637000324 Thiobacillus denitrificans
646564569 Sideroxydans lithotrophicus
648028028 Gallionella capsiferriformans
2516143014 Methylophilus unclassified
2512564015 unclassified unclassified
2517287022 Methylotenera mobilis
2516493010 Methylotenera versatilis

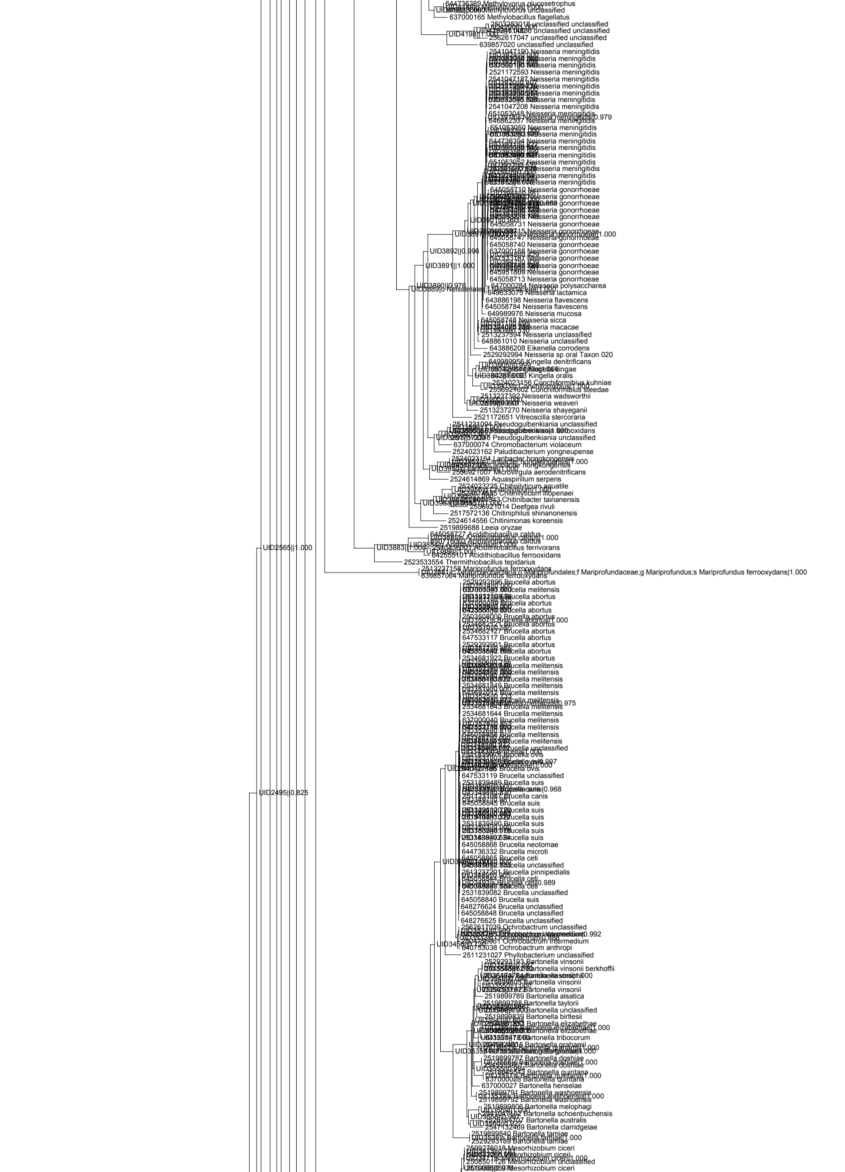

644736389 Methylovorus glucosetrophus
637000165 Methylobacillus flagellatus
2503283018 unclassified unclassified
2562617047 unclassified unclassified
639857020 unclassified unclassified
2541047190 Neisseria meningitidis
2521172593 Neisseria meningitidis
2541047208 Neisseria meningitidis
645058710 Neisseria gonorrhoeae
645058731 Neisseria gonorrhoeae
645058740 Neisseria gonorrhoeae
645058713 Neisseria gonorrhoeae
647000284 Neisseria polysaccharea
643886198 Neisseria flavescens
645058784 Neisseria flavescens
649989976 Neisseria mucosa
645058748 Neisseria sicca
2513237394 Neisseria unclassified
648861010 Neisseria unclassified
643886208 Eikenella corrodens
2529292994 Neisseria sp oral Taxon 020
2524023156 Conchiformibius kuhniae
2513237270 Neisseria shayeganii
2521172651 Vitreoscilla stercoraria
637000074 Chromobacterium violaceum
2524023162 Paludibacterium yongneupense
2524614869 Aquaspirillum serpens
2556921014 Deefgea rivuli
2517572136 Chitiniphilus shinanonensis
2524614556 Chitinimonas koreensis
2519899688 Leeia oryzae
642555101 Acidithiobacillus ferrooxidans
2523533554 Thermithiobacillus tepidarius
UID2565||1.000
UID2495||0.825
UID3892||0.996
UID3891||1.000
UID3883||1.000
647533117 Brucella abortus
2534681644 Brucella melitensis
647533119 Brucella unclassified
645058845 Brucella suis
645058868 Brucella neotomae
644736332 Brucella microti
2531839082 Brucella unclassified
645058840 Brucella suis
648276624 Brucella unclassified
645058848 Brucella unclassified
648276625 Brucella unclassified
640753038 Ochrobactrum anthropi
2511231027 Phyllobacterium unclassified
2519899789 Bartonella alsatica
2519899839 Bartonella birtlesii
637000027 Bartonella henselae
2547132469 Bartonella clarridgeiae

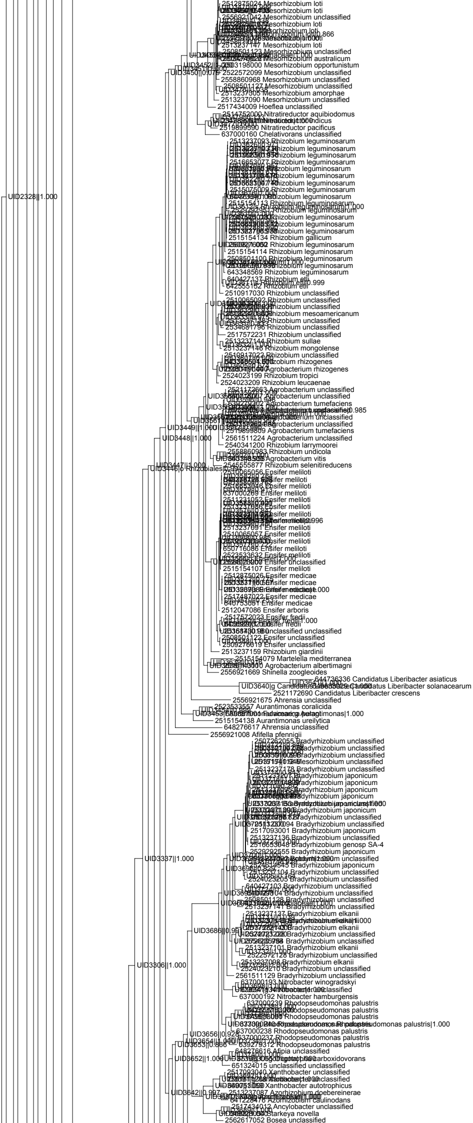
2512875024 Mesorhizobium loti
2556921042 Mesorhizobium unclassified
2513237147 Mesorhizobium loti
2508501123 Mesorhizobium unclassified
2503198000 Mesorhizobium opportunistum
2522572099 Mesorhizobium unclassified
2558860968 Mesorhizobium unclassified
2508501127 Mesorhizobium unclassified
2513237305 Mesorhizobium amorphae
2513237090 Mesorhizobium unclassified
2517434009 Hoeflea unclassified
2514752000 Nitratireductor aquibiodomus
2516899590 Nitratireductor pacificus
637000160 Chelativorans unclassified
UID2328||1.000
2513237093 Rhizobium leguminosarum
2516653077 Rhizobium leguminosarum
2515075009 Rhizobium leguminosarum
2515154113 Rhizobium leguminosarum
2515154134 Rhizobium gallicum
2515154114 Rhizobium leguminosarum
2508501100 Rhizobium leguminosarum
643348569 Rhizobium leguminosarum
640427137 Rhizobium etli
642555152 Rhizobium etli
2510917030 Rhizobium unclassified
2510065092 Rhizobium unclassified
2534681796 Rhizobium unclassified
2517572231 Rhizobium unclassified
2513237144 Rhizobium sullae
2513237146 Rhizobium mongolense
2510917022 Rhizobium unclassified
2524023199 Rhizobium tropici
2524023209 Rhizobium leucaenae
2521172663 Agrobacterium unclassified
2561511224 Agrobacterium unclassified
2540341200 Rhizobium larrymoorei
2558860983 Rhizobium undicola
2545555877 Rhizobium selenitireducens
UID3449||1.000
UID3448||1.000
UID3447||1.000
UID3446|o Rhizobiales|0.992
2510065056 Ensifer meliloti
637000269 Ensifer meliloti
2511231052 Ensifer meliloti
2513237091 Ensifer meliloti
2510065057 Ensifer meliloti
650716086 Ensifer meliloti
2523533632 Ensifer meliloti
2515154107 Ensifer meliloti
2512875026 Ensifer medicae
2517487022 Ensifer medicae
640753061 Ensifer medicae
2512047086 Ensifer arboris
2517572023 Ensifer fredii
2508501122 Ensifer unclassified
2509276019 Ensifer unclassified
2513237159 Rhizobium giardinii
2515154079 Martelella mediterranea
2556921669 Shinella zoogleoides
644736336 Candidatus Liberibacter asiaticus
UID3640|g Candidatus Liberibacter|1.000
2521172690 Candidatus Liberibacter crescens
2556921675 Ahrensia unclassified
2523533557 Aurantimonas coralicida
2515154138 Aurantimonas ureilytica
648276617 Ahrensia unclassified
2556921008 Afifella pfennigii
2507262055 Bradyrhizobium unclassified
2513237178 Bradyrhizobium unclassified
2517093001 Bradyrhizobium japonicum
2513237136 Bradyrhizobium unclassified
2516653048 Bradyrhizobium genosp SA-4
2529292555 Bradyrhizobium japonicum
UID3337||1.000
2513237104 Bradyrhizobium unclassified
2524023205 Bradyrhizobium unclassified
640427103 Bradyrhizobium unclassified
2508501128 Bradyrhizobium unclassified
2513237141 Bradyrhizobium unclassified
2513237137 Bradyrhizobium elkanii
UID3686||0.951
2513237101 Bradyrhizobium elkanii
2522572128 Bradyrhizobium unclassified
UID3306||1.000
2513237098 Bradyrhizobium elkanii
2524023210 Bradyrhizobium unclassified
2561511129 Bradyrhizobium unclassified
637000193 Nitrobacter winogradskyi
637000192 Nitrobacter hamburgensis
637000239 Rhodopseudomonas palustris
637000238 Rhodopseudomonas palustris
637000237 Rhodopseudomonas palustris
639279312 Rhodopseudomonas palustris
UID3656||0.923
UID3653||0.868
648276616 Afipia unclassified
UID3652||1.000
651324015 unclassified unclassified
2517093040 Xanthobacter unclassified
UID3642||0.997
2513237087 Azorhizobium doebereinerae
641228476 Azorhizobium caulinodans
2517434012 Ancylobacter unclassified
2562617052 Bosea unclassified

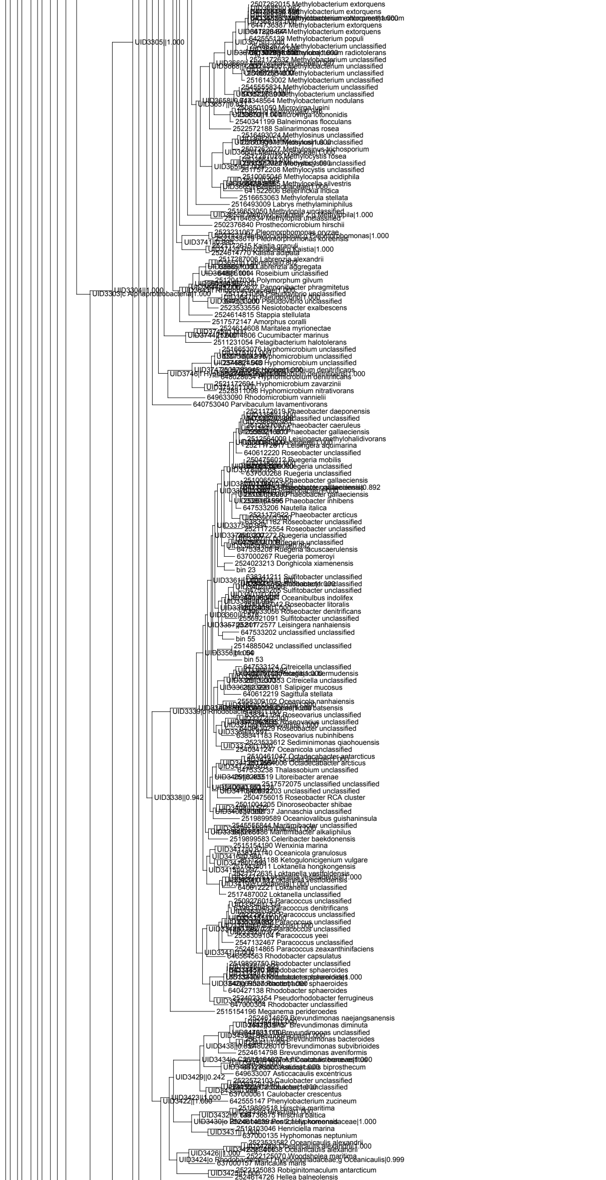

2507262015 Methylobacterium extorquens
644736387 Methylobacterium extorquens
642555139 Methylobacterium populi
2521172632 Methylobacterium unclassified
2516143002 Methylobacterium unclassified
643348564 Methylobacterium nodulans
2508501050 Microvirga lupini
2540341199 Balneimonas flocculans
2522572188 Salinarimonas rosea
2516493024 Methylosinus unclassified
2507262027 Methylosinus trichosporium
2517572208 Methylocystis unclassified
2510065046 Methylocapsa acidiphila
641522606 Beijerinckia indica
2516653063 Methyloferula stellata
2516493009 Labrys methylaminiphilus
2516653050 Methylopila unclassified
2502376840 Prosthecomicrobium hirschii
2523231067 Pleomorphomonas oryzae
2524614770 Kaistia adipata
2517287006 Labrenzia alexandrii
2512047034 Polymorphum gilvum
2523533556 Nesiotobacter exalbescens
2524614815 Stappia stellulata
2517572147 Amorphus coralli
2524614608 Maritalea myrionectae
2511231054 Pelagibacterium halotolerans
2516653076 Hyphomicrobium unclassified
649633090 Rhodomicrobium vannielii
640753040 Parvibaculum lavamentivorans
2521172619 Phaeobacter daeponensis
640612220 Roseobacter unclassified
2504756012 Ruegeria mobilis
637000268 Ruegeria unclassified
2510065029 Phaeobacter gallaeciensis
647533206 Nautella italica
2521172622 Phaeobacter arcticus
2521172554 Roseobacter unclassified
647533208 Ruegeria lacuscaerulensis
637000267 Ruegeria pomeroyi
2524023213 Donghicola xiamenensis
bin 23
2556921091 Sulfitobacter unclassified
2521172577 Leisingera nanhaiensis
647533202 unclassified unclassified
bin 55
2514885042 unclassified unclassified
bin 53
647533124 Citreicella unclassified
640612219 Sagittula stellata
2558309102 Oceanicola nanhaiensis
638341183 Roseovarius nubinhibens
2523533612 Sediminimonas qiaohouensis
2540341247 Oceanicola unclassified
2510461047 Octadecabacter antarcticus
647533238 Thalassobium unclassified
2517572075 unclassified unclassified
2504756015 Roseobacter RCA cluster
2501004205 Dinoroseobacter shibae
2519899589 Oceaniovalibus guishaninsula
2519899583 Celeribacter baekdonensis
2515154190 Wenxinia marina
2517487002 Loktanella unclassified
2509276015 Paracoccus unclassified
2558309104 Paracoccus yeei
2547132467 Paracoccus unclassified
2524614865 Paracoccus zeaxanthinifaciens
646564563 Rhodobacter capsulatus
2519899760 Rhodobacter unclassified
640427138 Rhodobacter sphaeroides
2524023154 Pseudorhodobacter ferrugineus
647000304 Rhodobacter unclassified
2515154196 Meganema perideroedes
2524614659 Brevundimonas naejangsanensis
2524614798 Brevundimonas aveniformis
649633007 Asticcacaulis excentricus
637000061 Caulobacter crescentus
642555147 Phenylobacterium zucineum
2519899518 Hirschia maritima
2519103046 Henriciella marina
637000135 Hyphomonas neptunium
2523533582 Oceanicaulis alexandrii
2522125070 Woodsholea maritima
637000157 Maricaulis maris
2522125083 Robiginitomaculum antarcticum
2524614726 Hellea balneolensis
UID3303|c Alphaproteobacteria|1.000
UID3338||0.942
UID3422||1.000

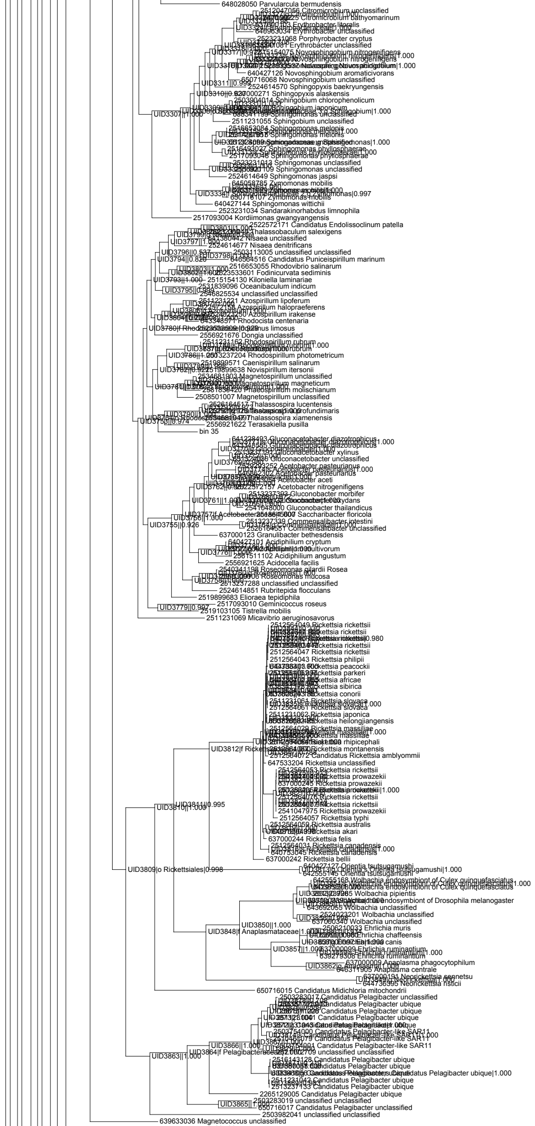

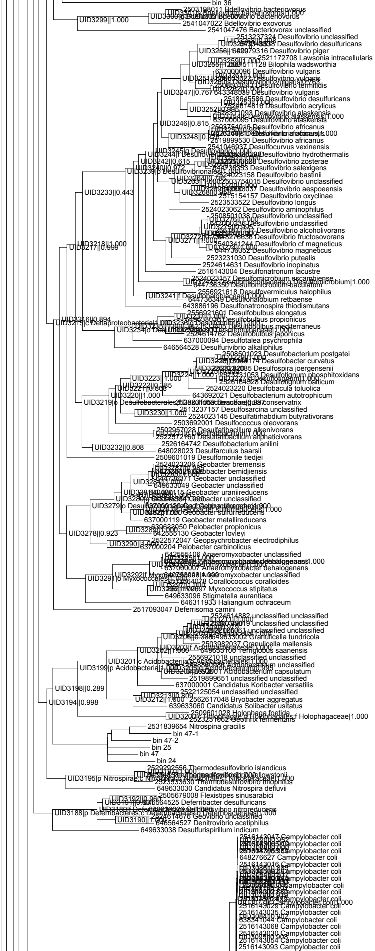
bin 36
2503198011 Bdellovibrio bacteriovorus
UID3299||1.000
2541047022 Bdellovibrio exovorus
2541047476 Bacteriovorax unclassified
2513237324 Desulfovibrio unclassified
637000079316 Desulfovibrio piger
2521172708 Lawsonia intracellularis
637000096 Desulfovibrio vulgaris
UID3247||0.767 643348539 Desulfovibrio vulgaris
2518645586 Desulfovibrio desulfuricans
2524614816 Desulfovibrio acrylicus
637000095 Desulfovibrio alaskensis
UID3246||0.815
2519899530 Desulfovibrio africanus
2541046937 Desulfocurvus vexinensis
Desulfovibrio hydrothermalis
Desulfovibrio zosterae
Desulfovibrio salexigens
2524023158 Desulfovibrio bastinii
Desulfovibrio unclassified
Desulfovibrio aespoeensis
2515154157 Desulfovibrio oxyclinae
2523533522 Desulfovibrio longus
2524023062 Desulfovibrio aminophilus
2508501038 Desulfovibrio unclassified
Desulfovibrio unclassified
Desulfovibrio alcoholivorans
Desulfovibrio fructosovorans
2540341244 Desulfovibrio cf magneticus
644736352 Desulfovibrio magneticus
UID3233||0.443
UID3217||0.999
2523231030 Desulfovibrio putealis
2524614631 Desulfovibrio inopinatus
2516143004 Desulfonatronum lacustre
2524023157 Desulfomicrobium escambiense
Desulfomicrobium baculatum
UID3241|f Desulfohalobiaceae|1.000
2556921618 Desulfovermiculus halophilus
644736349 Desulfohalobium retbaense
643886196 Desulfonatronospira thiodismutans
2556921601 Desulfobulbus elongatus
Desulfobulbus propionicus
UID3216||0.894
Desulfobulbus mediterraneus
2524614762 Desulfobulbus japonicus
637000094 Desulfotalea psychrophila
646564528 Desulfurivibrio alkaliphilus
2508501023 Desulfobacterium postgatei
Desulfobacter curvatus
Desulfospira joergensenii
Desulfotignum phosphitoxidans
Desulfotignum balticum
2524023220 Desulfobacula toluolica
643692021 Desulfobacterium autotrophicum
UID3220||1.000
UID3230||1.000
2513237157 Desulfosarcina unclassified
2524023145 Desulfatirhabdium butyrativorans
2503692001 Desulfococcus oleovorans
2502957028 Desulfatibacillum alkenivorans
Desulfatibacillum aliphaticivorans
UID3232||0.808
2526164742 Desulfobacterium anilini
648028023 Desulfarculus baarsii
2509601019 Desulfomonile tiedjei
2524023206 Geobacter bremensis
Geobacter bemidjiensis
Geobacter unclassified
649633049 Geobacter unclassified
Geobacter uraniireducens
Geobacter unclassified
Geobacter sulfurreducens
637000119 Geobacter metallireducens
UID3278||0.923
639633050 Pelobacter propionicus
642555130 Geobacter lovleyi
2522572047 Geopsychrobacter electrodiphilus
637000204 Pelobacter carbinolicus
642555106 Anaeromyxobacter unclassified
637000007 Anaeromyxobacter dehalogenans
Anaeromyxobacter unclassified
Corallococcus coralloides
Myxococcus stipitatus
649633096 Stigmatella aurantiaca
646311933 Haliangium ochraceum
2517093047 Deferrisoma camini
2524614882 unclassified unclassified
unclassified unclassified
unclassified unclassified
Granulicella tundricola
2503982037 Granulicella mallensis
649633100 Terriglobus saanensis
2556921018 unclassified unclassified
Acidobacterium unclassified
Acidobacterium capsulatum
2519899651 unclassified unclassified
637000001 Candidatus Koribacter versatilis
UID3198||0.289
2522125054 unclassified unclassified
2562617048 Bryobacter aggregatus
UID3194||0.998
639633060 Candidatus Solibacter usitatus
2509601028 Holophaga foetida
2523231062 Geothrix fermentans
2531839654 Nitrospina gracilis
bin 47-1
bin 47-2
bin 25
bin 47
bin 24
2529292556 Thermodesulfovibrio islandicus
Thermodesulfovibrio yellowstonii
2523533630 Thermodesulfovibrio thiophilus
649633030 Candidatus Nitrospira defluvii
2505679008 Flexistipes sinusarabici
646564525 Deferribacter desulfuricans
Denitrovibrio nitroreducens
2524614676 Geovibrio unclassified
UID3190||1.000
646564527 Denitrovibrio acetiphilus
649633038 Desulfurispirillum indicum
2516143047 Campylobacter coli
Campylobacter coli
648276627 Campylobacter coli
2516143016 Campylobacter coli
Campylobacter coli
2516143029 Campylobacter coli
2516143035 Campylobacter coli
638341044 Campylobacter coli
2516143068 Campylobacter coli
2516143030 Campylobacter coli
2516143054 Campylobacter coli
2516143093 Campylobacter coli

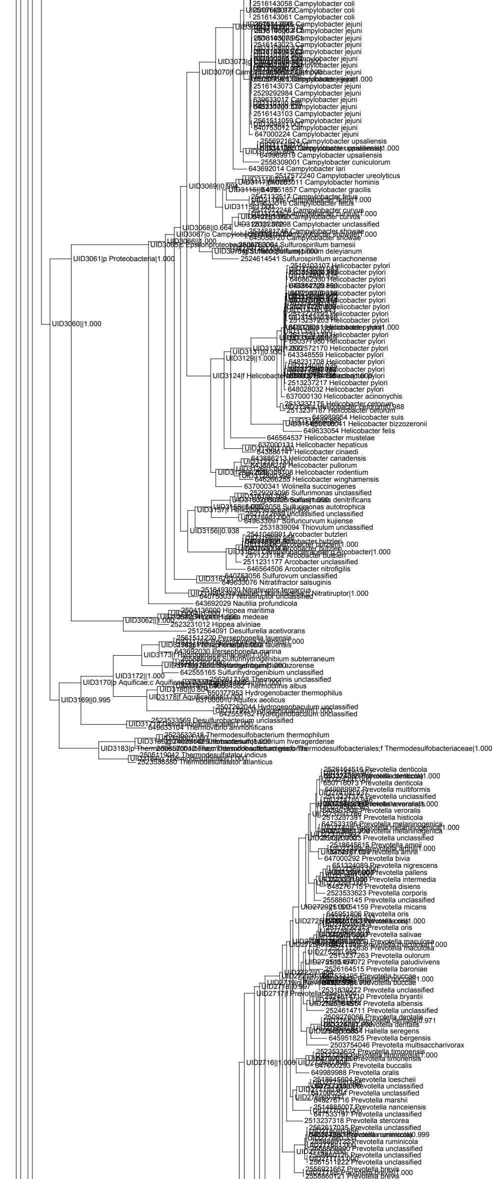
2516143058 Campylobacter coli
2516143061 Campylobacter coli
2516143023 Campylobacter jejuni
2516143073 Campylobacter jejuni
2529292984 Campylobacter jejuni
639633017 Campylobacter jejuni
2516143103 Campylobacter jejuni
2561511059 Campylobacter jejuni
640753012 Campylobacter jejuni
647000224 Campylobacter jejuni
2556921624 Campylobacter upsaliensis
649989919 Campylobacter upsaliensis
2558309001 Campylobacter cuniculorum
643692014 Campylobacter lari
2517572240 Campylobacter ureolyticus
UID3069||0.994
UID3115||1.000
2503230298 Campylobacter unclassified
UID3068||0.664
2534681746 Campylobacter showae
645058720 Campylobacter showae
UID3066||1.000
2524614541 Sulfurospirillum arcachonense
UID3061|p Proteobacteria|1.000
2519103107 Helicobacter pylori
646862330 Helicobacter pylori
2513237203 Helicobacter pylori
650377960 Helicobacter pylori
UID3060||1.000
643348559 Helicobacter pylori
UID3129||1.000
648231708 Helicobacter pylori
2513237217 Helicobacter pylori
648028032 Helicobacter pylori
637000130 Helicobacter acinonychis
2513237187 Helicobacter cetorum
649989954 Helicobacter suis
649633054 Helicobacter felis
646564537 Helicobacter mustelae
637000131 Helicobacter hepaticus
643886141 Helicobacter cinaedi
643886213 Helicobacter canadensis
646206255 Helicobacter winghamensis
637000341 Wolinella succinogenes
2529293096 Sulfurimonas unclassified
649633097 Sulfuricurvum kujiense
2531839094 Thiovulum unclassified
UID3156||0.938
2541046991 Arcobacter butzleri
2511231182 Arcobacter butzleri
2511231177 Arcobacter unclassified
646564506 Arcobacter nitrofigilis
640753056 Sulfurovum unclassified
649633076 Nitratifractor salsuginis
2516493030 Nitratiruptor tergarcus
640753037 Nitratiruptor unclassified
643692029 Nautilia profundicola
2504136000 Hippea maritima
UID3062||1.000
2523231012 Hippea alviniae
2512564091 Desulfurella acetivorans
2561511220 Persephonella lauensis
643692030 Persephonella marina
642555165 Sulfurihydrogenibium unclassified
UID3172||1.000
2562617198 Thermocrinis unclassified
UID3170|p Aquificae;c Aquificae
UID3180||0.804
650377953 Hydrogenobacter thermophilus
UID3169||0.995
637000010 Aquifex aeolicus
2507262044 Hydrogenobaculum unclassified
642555132 Hydrogenobaculum unclassified
2523533569 Desulfurobacterium unclassified
649633104 Thermovibrio ammonificans
2523533618 Thermodesulfobacterium thermophilum
UID3183|p Thermodesulfobacteria
2505119042 Thermodesulfatator indicus
2523533580 Thermodesulfatator atlanticus
2526164516 Prevotella denticola
650716073 Prevotella denticola
649989987 Prevotella multiformis
2513237374 Prevotella unclassified
645951836 Prevotella veroralis
2513237391 Prevotella histicola
647533196 Prevotella melaninogenica
2562617023 Prevotella unclassified
2518645615 Prevotella amnii
647000292 Prevotella bivia
651324089 Prevotella nigrescens
648276715 Prevotella disiens
2523533623 Prevotella corporis
2558860145 Prevotella unclassified
645951806 Prevotella oris
2513237263 Prevotella oulorum
2526164515 Prevotella baroniae
2531839272 Prevotella unclassified
2524614711 Prevotella unclassified
2509276066 Prevotella dentalis
645951825 Prevotella bergensis
2503754046 Prevotella multisaccharivorax
2523533627 Prevotella timonensis
647000293 Prevotella buccalis
649989988 Prevotella oralis
2518645604 Prevotella loescheii
648276716 Prevotella marshii
2514885007 Prevotella nanceiensis
647533197 Prevotella unclassified
2513237318 Prevotella stercorea
2562617035 Prevotella unclassified
2556921667 Prevotella brevis

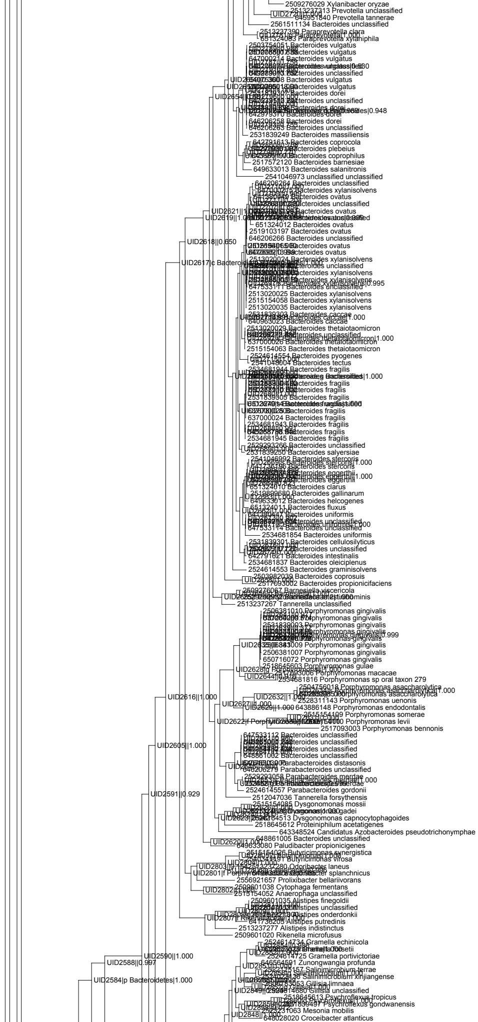

2509276029 Xylanibacter oryzae
2513237313 Prevotella unclassified
646951840 Prevotella tannerae
2561511134 Bacteroides unclassified
2513237390 Paraprevotella clara
651324083 Paraprevotella xylaniphila
2503754051 Bacteroides vulgatus
647000214 Bacteroides vulgatus
2531839107 Bacteroides dorei
642979370 Bacteroides dorei
646206258 Bacteroides dorei
646206263 Bacteroides unclassified
2531839249 Bacteroides massiliensis
642791613 Bacteroides coprocola
2517572120 Bacteroides barnesiae
649633013 Bacteroides salanitronis
2541046973 unclassified unclassified
646206264 Bacteroides unclassified
UID2621||1.000
UID2619||1.000
651324012 Bacteroides ovatus
2519103197 Bacteroides ovatus
646206266 Bacteroides unclassified
UID2618||0.650
UID2617|c Bacteroidia
2513020024 Bacteroides xylanisolvens
647533111 Bacteroides unclassified
2513020025 Bacteroides xylanisolvens
2515154058 Bacteroides xylanisolvens
2513020035 Bacteroides xylanisolvens
2531839303 Bacteroides caccae
640963023 Bacteroides caccae
2513020029 Bacteroides thetaiotaomicron
637000026 Bacteroides thetaiotaomicron
2515154063 Bacteroides thetaiotaomicron
2524614554 Bacteroides pyogenes
2541046004 Bacteroides tectus
2534681944 Bacteroides fragilis
2531839305 Bacteroides fragilis
637000024 Bacteroides fragilis
2534681943 Bacteroides fragilis
2534681945 Bacteroides fragilis
2529293266 Bacteroides unclassified
2531839250 Bacteroides salyersiae
2541046992 Bacteroides stercoris
651324010 Bacteroides clarus
2519899680 Bacteroides gallinarum
649633012 Bacteroides helcogenes
651324011 Bacteroides fluxus
647533114 Bacteroides unclassified
2534681854 Bacteroides uniformis
2531839301 Bacteroides cellulosilyticus
642791621 Bacteroides intestinalis
2534681837 Bacteroides oleiciplenus
2524614553 Bacteroides graminisolvens
2503982039 Bacteroides coprosuis
2517093002 Bacteroides propionicifaciens
2509276067 Barnesiella viscericola
2513237267 Tannerella unclassified
2506381010 Porphyromonas gingivalis
2531839003 Porphyromonas gingivalis
2506381007 Porphyromonas gingivalis
650716072 Porphyromonas gingivalis
2518645603 Porphyromonas gulae
2534681816 Porphyromonas sp oral taxon 279
2504756018 Porphyromonas asaccharolytica
2528311143 Porphyromonas uenonis
UID2616||1.000
UID2632||1.000
UID2627||1.000
UID2629||1.000 643886148 Porphyromonas endodontalis
2515154199 Porphyromonas somerae
Porphyromonas levii
2517093003 Porphyromonas bennonis
647533112 Bacteroides unclassified
646861002 Bacteroides unclassified
UID2605||1.000
646206279 Parabacteroides unclassified
2529293058 Parabacteroides merdae
2524614557 Parabacteroides gordonii
2512047036 Tannerella forsythensis
UID2591||0.929
2515154085 Dysgonomonas mossii
2524614513 Dysgonomonas capnocytophagoides
2518645612 Proteiniphilum acetatigenes
643348524 Candidatus Azobacteroides pseudotrichonymphae
UID2620||1.000
648861005 Bacteroides unclassified
649633080 Paludibacter propionicigenes
2515154026 Butyricimonas synergistica
Butyricimonas virosa
2513237280 Odoribacter laneus
Odoribacter splanchnicus
2556921657 Prolixibacter bellariivorans
UID2802||1.000
2509601038 Cytophaga fermentans
2515154052 Anaerophaga unclassified
2509601035 Alistipes finegoldii
Alistipes unclassified
Alistipes onderdonkii
641736205 Alistipes putredinis
2513237277 Alistipes indistinctus
2509601020 Rikenella microfusus
2524614734 Gramella echinicola
UID2590||1.000
UID2588||0.997
2524614725 Gramella portivictoriae
646564591 Zunongwangia profunda
UID2584|p Bacteroidetes|1.000
2522125157 Salinimicrobium terrae
Salinimicrobium xinjiangense
Gillisia limnaea
Gillisia unclassified
2518645613 Psychroflexus tropicus
2531839497 Psychroflexus gondwanensis
Mesonia mobilis
648028020 Croceibacter atlanticus

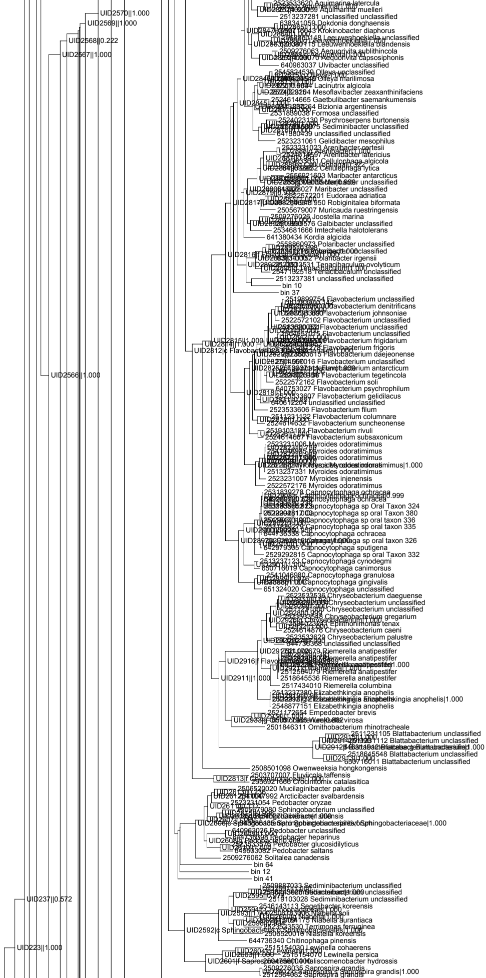

UID2570||1.000
UID2569||1.000
UID2568||0.222
UID2567||1.000
UID2566||1.000
UID237||0.572
UID223||1.000
2523533620 Aquimarina latercula
2513237281 unclassified unclassified
638341059 Dokdonia donghaensis
2509276063 Aequorivita sublithincola
640963037 Ulvibacter unclassified
2545824539 Olleya unclassified
2524614665 Gaetbulibacter saemankumensis
2531839038 Formosa unclassified
2524023130 Psychroserpens burtonensis
641380439 unclassified unclassified
2523231061 Gelidibacter mesophilus
2523231023 Arenibacter certesii
2505679007 Muricauda ruestringensis
2509276026 Joostella marina
2534681666 Imtechella halotolerans
641380434 Kordia algicida
2558860973 Polaribacter unclassified
2513237381 unclassified unclassified
bin 10
bin 37
2519899754 Flavobacterium unclassified
2522572102 Flavobacterium unclassified
2522572162 Flavobacterium soli
640753027 Flavobacterium psychrophilum
640612204 unclassified unclassified
2523533606 Flavobacterium filum
2511231122 Flavobacterium columnare
2524614632 Flavobacterium suncheonense
2519103183 Flavobacterium rivuli
2524614667 Flavobacterium subsaxonicum
2523231006 Myroides odoratimimus
2513237331 Myroides odoratimimus
2523231007 Myroides injenensis
2522572176 Myroides odoratimimus
2531839278 Capnocytophaga ochracea
644736358 Capnocytophaga ochracea
642979305 Capnocytophaga sputigena
2529292815 Capnocytophaga sp oral Taxon 332
2513237123 Capnocytophaga cynodegmi
650716019 Capnocytophaga canimorsus
2541046980 Capnocytophaga granulosa
651324020 Capnocytophaga unclassified
2523533536 Chryseobacterium daeguense
2524614876 Chryseobacterium caeni
2523533629 Chryseobacterium palustre
644736368 unclassified unclassified
2512564079 Riemerella anatipestifer
2518645536 Riemerella anatipestifer
2517434010 Riemerella columbina
2513237380 Elizabethkingia anophelis
2548877151 Elizabethkingia anophelis
2521172654 Empedobacter brevis
2501846311 Ornithobacterium rhinotracheale
2511231105 Blattabacterium unclassified
2518645548 Blattabacterium unclassified
650716011 Blattabacterium unclassified
2508501098 Owenweeksia hongkongensis
2503707007 Fluviicola taffensis
2506520020 Mucilaginibacter paludis
2523231054 Pedobacter oryzae
2505679080 Sphingobacterium unclassified
640963036 Pedobacter unclassified
649633082 Pedobacter saltans
2509276062 Solitalea canadensis
bin 64
bin 12
bin 41
2509887033 Sediminibacterium unclassified
2519103028 Sediminibacterium unclassified
2516143113 Segetibacter koreensis
2523533530 Terrimonas ferruginea
2506520019 Niastella koreensis
644736340 Chitinophaga pinensis
2515154030 Lewinella cohaerens
2515154070 Lewinella persica
2509276035 Saprospira grandis
UID2592|c Sphingobacteriia
UID2601|f Saprospiraceae
UID2816|f Flavobacteriaceae
UID2812|c Flavobacteriia
UID2911||1.000
UID2916|f Flavobacteriaceae
UID2813|f Cryomorphaceae|1.000

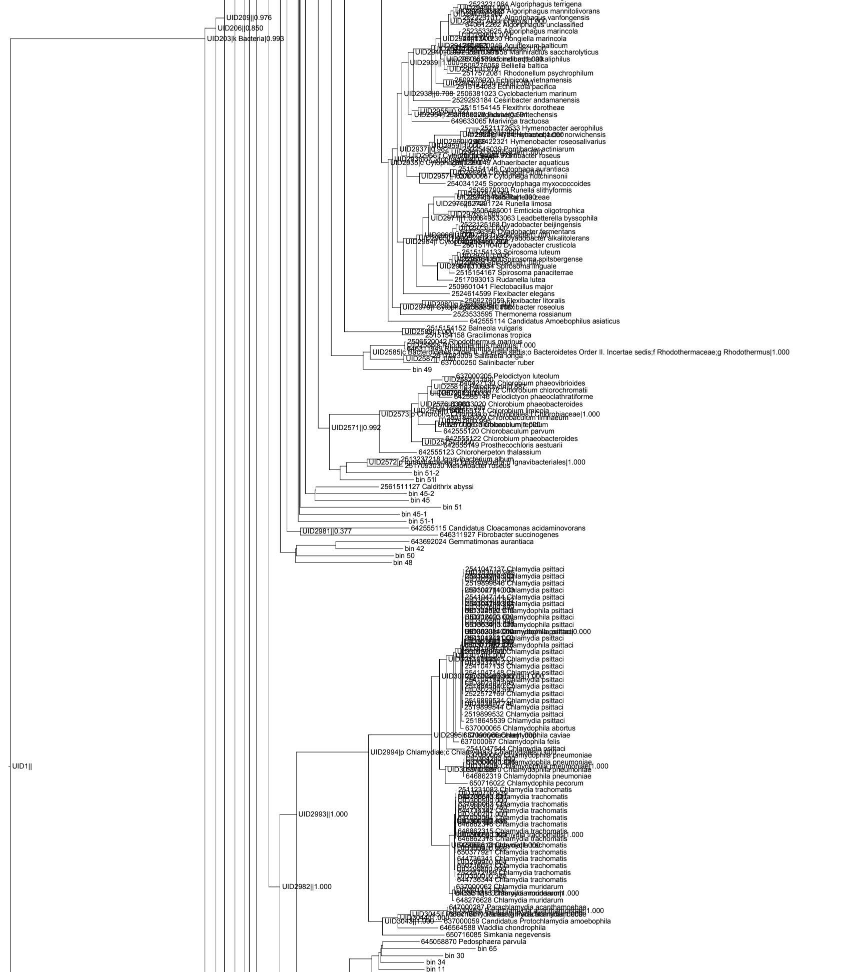
UID209||0.976
UID206||0.850
UID203|k Bacteria|0.993
UID1||
2523231064 Algoriphagus terrigena
Algoriphagus mannitolivorans
Algoriphagus vanfongensis
640612202 Algoriphagus unclassified
2523533625 Algoriphagus marincola
Hongiella marincola
Aquiflexum balticum
Mariniradius saccharolyticus
2509276058 Belliella baltica
2517572081 Rhodonellum psychrophilum
2509276020 Echinicola vietnamensis
2515154083 Echinicola pacifica
UID2939||1.000
UID2938||0.708
2506381023 Cyclobacterium marinum
2529293184 Cesiribacter andamanensis
2515154145 Flexithrix dorotheae
649633065 Marivirga tractuosa
2521172633 Hymenobacter aerophilus
Hymenobacter norwichensis
Hymenobacter roseosalivarius
Pontibacter actiniarum
Pontibacter roseus
Adhaeribacter aquaticus
2515154146 Cytophaga aurantiaca
Cytophaga hutchinsonii
2540341245 Sporocytophaga myxococcoides
2505679030 Runella slithyformis
Runella limosa
2506485001 Emticicia oligotrophica
649633063 Leadbetterella byssophila
Dyadobacter beijingensis
Dyadobacter fermentans
Dyadobacter alkalitolerans
2561511040 Dyadobacter crusticola
2515154133 Spirosoma luteum
Spirosoma spitsbergense
Spirosoma linguale
2515154167 Spirosoma panaciterrae
2517093013 Rudanella lutea
2509601041 Flectobacillus major
2524614599 Flexibacter elegans
2509276059 Flexibacter litoralis
Flexibacter roseolus
2523533595 Thermonema rossianum
642555114 Candidatus Amoebophilus asiaticus
2515154152 Balneola vulgaris
2515154158 Gracilimonas tropica
2506520042 Rhodothermus marinus
UID2585|c Bacteroidetes Order II. Incertae sedis;o Bacteroidetes Order II. Incertae sedis;f Rhodothermaceae;g Rhodothermus|1.000
Salisaeta longa
637000250 Salinibacter ruber
bin 49
637000205 Pelodictyon luteolum
Chlorobium phaeovibrioides
Chlorobium chlorochromatii
642555146 Pelodictyon phaeoclathratiforme
Chlorobium phaeobacteroides
Chlorobium limicola
Chlorobaculum limnaeum
UID2573|p Chlorobi;c Chlorobia;o Chlorobiales;f Chlorobiaceae|1.000
642555120 Chlorobaculum parvum
642555122 Chlorobium phaeobacteroides
642555149 Prosthecochloris aestuarii
642555123 Chloroherpeton thalassium
UID2571||0.992
2513237218 Ignavibacterium album
2517093030 Melioribacter roseus
bin 51-2
bin 51I
2561511127 Caldithrix abyssi
bin 45-2
bin 45
bin 51
bin 45-1
bin 51-1
UID2981||0.377
642555115 Candidatus Cloacamonas acidaminovorans
646311927 Fibrobacter succinogenes
643692024 Gemmatimonas aurantiaca
bin 42
bin 50
bin 48
2541047137 Chlamydia psittaci
2519899546 Chlamydia psittaci
2541047144 Chlamydia psittaci
Chlamydophila psittaci
2541047135 Chlamydia psittaci
2541047148 Chlamydia psittaci
2522572169 Chlamydia psittaci
2519899534 Chlamydia psittaci
2519899544 Chlamydia psittaci
2519899532 Chlamydia psittaci
2518645539 Chlamydia psittaci
637000065 Chlamydophila abortus
637000067 Chlamydophila felis
UID2994|p Chlamydiae;c Chlamydiia
2541047544 Chlamydia psittaci
Chlamydophila pneumoniae
646862319 Chlamydophila pneumoniae
650716022 Chlamydophila pecorum
2511231082 Chlamydia trachomatis
Chlamydia trachomatis
646862315 Chlamydia trachomatis
646862318 Chlamydia trachomatis
650377921 Chlamydia trachomatis
644736341 Chlamydia trachomatis
644736344 Chlamydia trachomatis
637000062 Chlamydia muridarum
648276628 Chlamydia muridarum
UID2993||1.000
UID2982||1.000
647000287 Parachlamydia acanthamoebae
637000059 Candidatus Protochlamydia amoebophila
646564588 Waddlia chondrophila
650716085 Simkania negevensis
645058870 Pedosphaera parvula
bin 65
bin 30
bin 34
bin 11

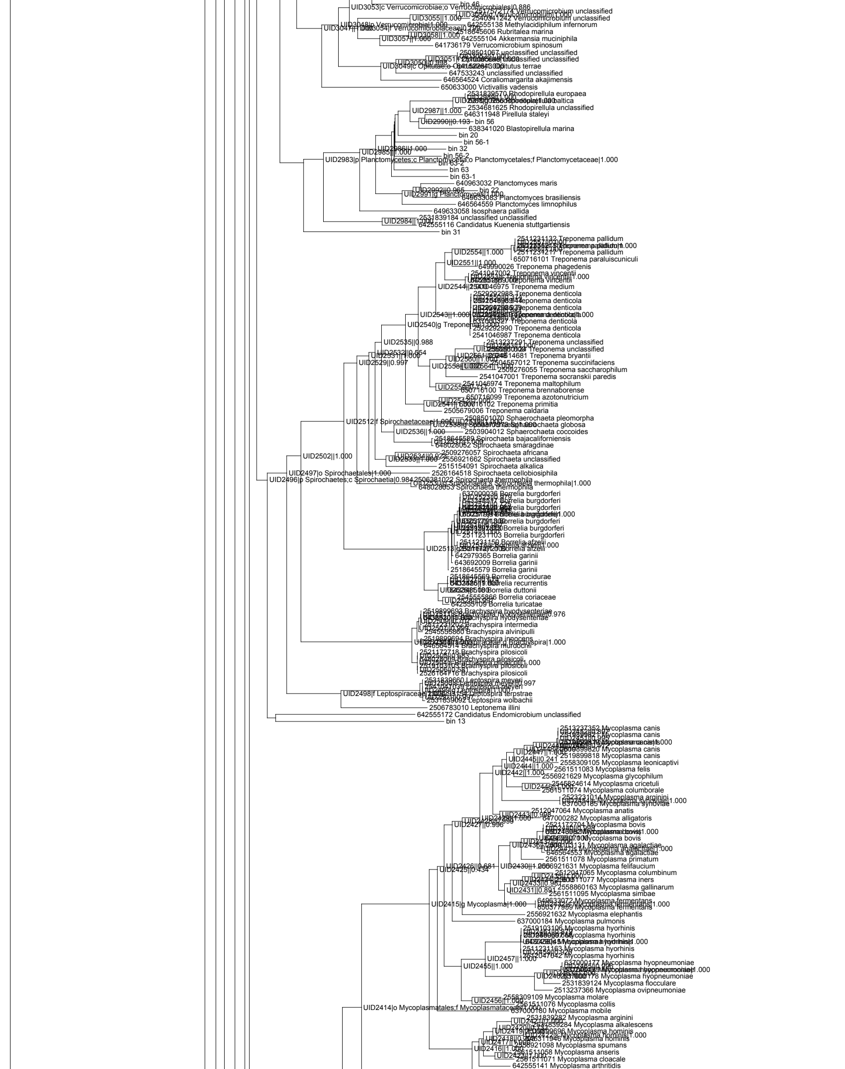
bin 46
UID3053|c Verrucomicrobiae;o Verrucomicrobiales|0.886
UID3055||1.000
642555138 Methylacidiphilum infernorum
2518645606 Rubritalea marina
UID3047||1.000
UID3057||1.000
642555104 Akkermansia muciniphila
641736179 Verrucomicrobium spinosum
2508501067 unclassified unclassified
UID3050||0.998
UID3049|c Opitutae;o Opitutales
647533243 unclassified unclassified
646564524 Coraliomargarita akajimensis
650633000 Victivallis vadensis
2531839570 Rhodopirellula europaea
UID2987||1.000
2534681625 Rhodopirellula unclassified
646311948 Pirellula staleyi
UID2990||0.193
bin 56
638341020 Blastopirellula marina
bin 20
bin 56-1
UID2986||1.000
UID2985||1.000
bin 32
bin 56-2
bin 63-2
UID2983|p Planctomycetes;c Planctomycetia;o Planctomycetales;f Planctomycetaceae|1.000
bin 63
bin 63-1
640963032 Planctomyces maris
UID2992||0.965
bin 22
UID2991|g Planctomyces|1.000
649633083 Planctomyces brasiliensis
646564559 Planctomyces limnophilus
649633058 Isosphaera pallida
2531839184 unclassified unclassified
UID2984||1.000
642555116 Candidatus Kuenenia stuttgartiensis
bin 31
2511231132 Treponema pallidum
UID2554||1.000
2511231217 Treponema pallidum
650716101 Treponema paraluiscuniculi
UID2551||1.000
649990026 Treponema phagedenis
2541047002 Treponema vincentii
UID2544||1.000
2541046975 Treponema medium
2529292988 Treponema denticola
UID2543||1.000
UID2540|g Treponema|1.000
2529292990 Treponema denticola
2541046987 Treponema denticola
UID2535||0.988
2513237291 Treponema unclassified
UID2532||0.554
UID2531||1.000
UID2529||0.997
2504557012 Treponema succinifaciens
2509276055 Treponema saccharophilum
2541047001 Treponema socranskii paredis
2541046974 Treponema maltophilum
650716100 Treponema brennaborense
650716099 Treponema azotonutricium
650716102 Treponema primitia
2505679006 Treponema caldaria
UID2512|f Spirochaetaceae|1.000
2508501070 Sphaerochaeta pleomorpha
UID2536||1.000
2503904012 Sphaerochaeta coccoides
2518645589 Spirochaeta bajacaliforniensis
648028052 Spirochaeta smaragdinae
UID2502||1.000
2509276057 Spirochaeta africana
2556921662 Spirochaeta unclassified
2515154091 Spirochaeta alkalica
UID2497|o Spirochaetales|1.000
2526164518 Spirochaeta cellobiosiphila
UID2496|p Spirochaetes;c Spirochaetia|0.984
648028053 Spirochaeta thermophila
637000036 Borrelia burgdorferi
2511231103 Borrelia burgdorferi
2511231159 Borrelia afzelii
642979365 Borrelia garinii
643692009 Borrelia garinii
2518645579 Borrelia garinii
2518645569 Borrelia crocidurae
2545555866 Borrelia coriaceae
642555109 Borrelia turicatae
2519899693 Brachyspira hyodysenteriae
2545555860 Brachyspira alvinipulli
2519899694 Brachyspira innocens
646564514 Brachyspira murdochii
2521172718 Brachyspira pilosicoli
2526164116 Brachyspira pilosicoli
2531839060 Leptospira meyeri
UID2498|f Leptospiraceae
2531839092 Leptospira wolbachii
2506783010 Leptonema illini
642555172 Candidatus Endomicrobium unclassified
bin 13
2513237352 Mycoplasma canis
2519899820 Mycoplasma canis
2519899818 Mycoplasma canis
UID2447||1.000
UID2445||0.241
UID2444||1.000
2558309105 Mycoplasma leonicaptivi
2561511083 Mycoplasma felis
UID2442||1.000
2556921629 Mycoplasma glycophilum
2545824614 Mycoplasma cricetuli
2561511074 Mycoplasma columborale
2523231014 Mycoplasma arginini
637000185 Mycoplasma synoviae
2512047064 Mycoplasma anatis
UID2443||0.998
647000282 Mycoplasma alligatoris
UID2427||0.996
2521172704 Mycoplasma bovis
646564553 Mycoplasma agalactiae
2561511078 Mycoplasma primatum
UID2426||0.681
UID2425||0.434
UID2430||1.000
2556921631 Mycoplasma felifaucium
2512047065 Mycoplasma columbinum
2561511077 Mycoplasma iners
UID2433||0.987
2558860163 Mycoplasma gallinarum
UID2431||0.891
2561511095 Mycoplasma simbae
UID2415|g Mycoplasma|1.000
649633072 Mycoplasma fermentans
650377955 Mycoplasma fermentans
2556921632 Mycoplasma elephantis
637000184 Mycoplasma pulmonis
2519103106 Mycoplasma hyorhinis
2511231163 Mycoplasma hyorhinis
UID2457||1.000
UID2455||1.000
637000177 Mycoplasma hyopneumoniae
637000178 Mycoplasma hyopneumoniae
2531839124 Mycoplasma flocculare
2513237366 Mycoplasma ovipneumoniae
2558309109 Mycoplasma molare
UID2456||1.000
2561511076 Mycoplasma collis
UID2414|o Mycoplasmatales;f Mycoplasmataceae
637000180 Mycoplasma mobile
2531839282 Mycoplasma arginini
2531839284 Mycoplasma alkalescens
2556921098 Mycoplasma spumans
UID2416||1.000
2561511058 Mycoplasma anseris
2561511071 Mycoplasma cloacale
642555141 Mycoplasma arthritidis

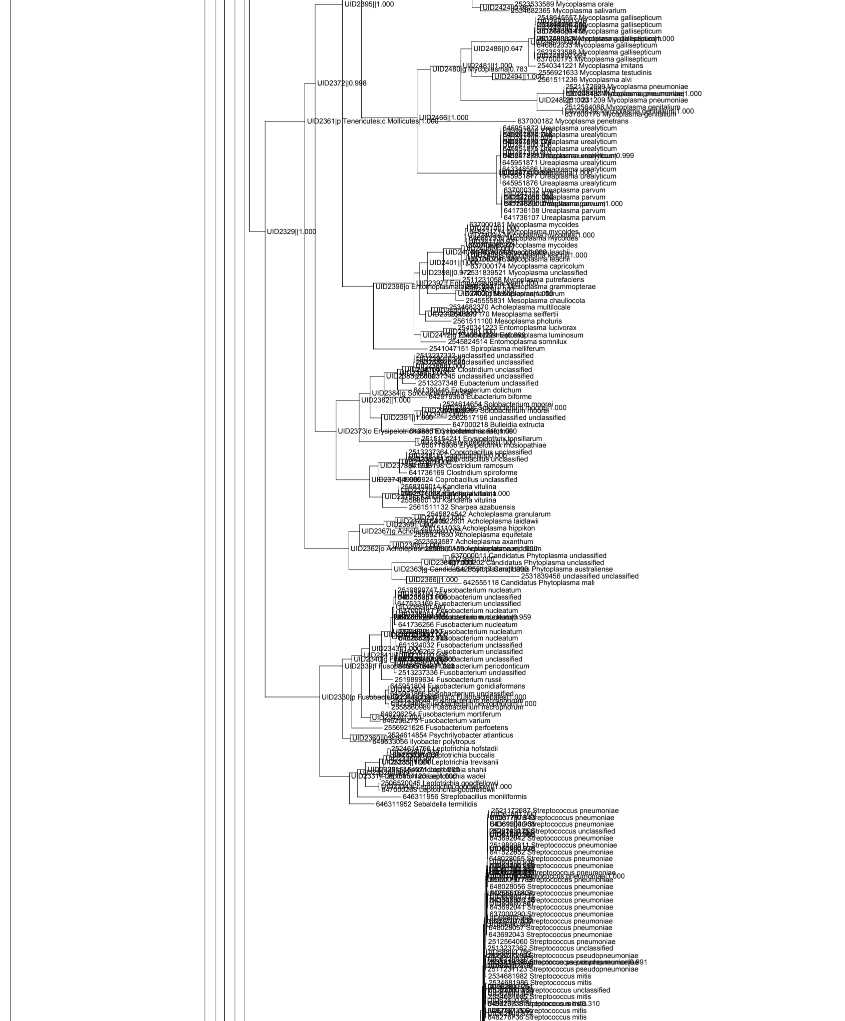

UID2395||1.000
2523533589 Mycoplasma orale
2534682365 Mycoplasma salivarium
2518645557 Mycoplasma gallisepticum
646862333 Mycoplasma gallisepticum
2523533588 Mycoplasma gallisepticum
637000175 Mycoplasma gallisepticum
UID2486||0.647
UID2481||1.000
2540341221 Mycoplasma imitans
UID2480|g Mycoplasma|0.783
2556921633 Mycoplasma testudinis
UID2494||1.000
2561511236 Mycoplasma alvi
UID2372||0.998
2521172699 Mycoplasma pneumoniae
2511231209 Mycoplasma pneumoniae
2512564088 Mycoplasma genitalium
637000176 Mycoplasma genitalium
UID2466||1.000
UID2361|p Tenericutes;c Mollicutes|1.000
637000182 Mycoplasma penetrans
645951872 Ureaplasma urealyticum
645951871 Ureaplasma urealyticum
645951877 Ureaplasma urealyticum
645951876 Ureaplasma urealyticum
637000332 Ureaplasma parvum
641736108 Ureaplasma parvum
641736107 Ureaplasma parvum
UID2329||1.000
637000181 Mycoplasma mycoides
637000174 Mycoplasma capricolum
UID2401||1.000
UID2398||0.972
2531839521 Mycoplasma unclassified
2511231058 Mycoplasma putrefaciens
UID2396|o Entomoplasmatales
2545555831 Mesoplasma chauliocola
2534682370 Acholeplasma multilocale
2561511100 Mesoplasma photuris
2540341223 Entomoplasma lucivorax
2545824514 Entomoplasma somnilux
2541047151 Spiroplasma melliferum
2513237345 unclassified unclassified
2513237348 Eubacterium unclassified
641380446 Eubacterium dolichum
642979360 Eubacterium biforme
UID2382||1.000
2524614654 Solobacterium moorei
UID2391||1.000
2562617196 unclassified unclassified
647000218 Bulleidia extructa
UID2373|o Erysipelotrichales
2515154211 Erysipelothrix tonsillarum
650716066 Erysipelothrix rhusiopathiae
2513237364 Coprobacillus unclassified
641736169 Clostridium spiroforme
2558309014 Kandleria vitulina
2558860130 Kandleria vitulina
2561511132 Sharpea azabuensis
2545824542 Acholeplasma granularum
2561511033 Acholeplasma hippikon
2556921630 Acholeplasma equifetale
2523533587 Acholeplasma axanthum
UID2362|o Acholeplasmatales
637000011 Candidatus Phytoplasma unclassified
UID2363|g Candidatus Phytoplasma
2531839456 unclassified unclassified
UID2366||1.000
642555118 Candidatus Phytoplasma mali
2519899747 Fusobacterium nucleatum
647533169 Fusobacterium unclassified
641736256 Fusobacterium nucleatum
651324032 Fusobacterium unclassified
2513237336 Fusobacterium unclassified
2519899634 Fusobacterium russii
645951804 Fusobacterium gonidiaformans
2558860969 Fusobacterium necrophorum
646206254 Fusobacterium mortiferum
646206273 Fusobacterium varium
2556921626 Fusobacterium perfoetens
UID2330|p Fusobacteria
2524614854 Psychrilyobacter atlanticus
649633056 Ilyobacter polytropus
2524614766 Leptotrichia hofstadii
2506520045 Leptotrichia goodfellowii
647000268 Leptotrichia goodfellowii
646311956 Streptobacillus moniliformis
646311952 Sebaldella termitidis
2521172687 Streptococcus pneumoniae
643692042 Streptococcus pneumoniae
2519899811 Streptococcus pneumoniae
641522652 Streptococcus pneumoniae
648028055 Streptococcus pneumoniae
648028056 Streptococcus pneumoniae
643692041 Streptococcus pneumoniae
637000290 Streptococcus pneumoniae
643692043 Streptococcus pneumoniae
2512564060 Streptococcus pneumoniae
2513237362 Streptococcus unclassified
2511231123 Streptococcus pseudopneumoniae
2534681982 Streptococcus mitis
2534681986 Streptococcus mitis
648276736 Streptococcus mitis

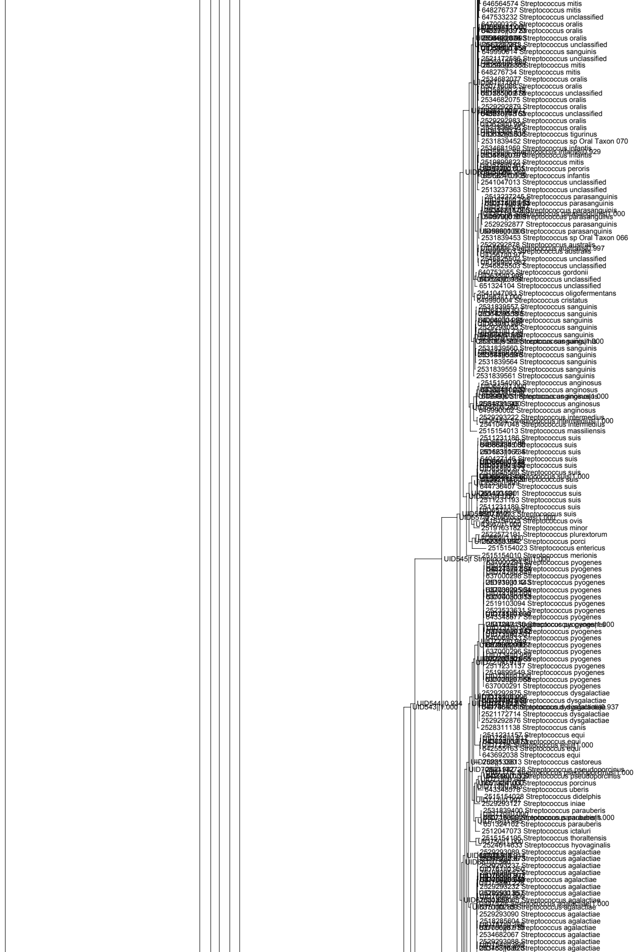
646564574 Streptococcus mitis
648276737 Streptococcus mitis
647533232 Streptococcus unclassified
649990014 Streptococcus sanguinis
2521172586 Streptococcus unclassified
648276734 Streptococcus mitis
2534682077 Streptococcus oralis
2534682075 Streptococcus oralis
2529292879 Streptococcus oralis
2529292983 Streptococcus oralis
2531839452 Streptococcus sp Oral Taxon 070
2534681959 Streptococcus infantis
2518899822 Streptococcus mitis
2541047013 Streptococcus unclassified
2513237363 Streptococcus unclassified
2513237245 Streptococcus parasanguinis
2529292877 Streptococcus parasanguinis
2531839453 Streptococcus sp Oral Taxon 066
2529292878 Streptococcus australis
2546525502 Streptococcus unclassified
2546525503 Streptococcus unclassified
640753055 Streptococcus gordonii
651324104 Streptococcus unclassified
2541047083 Streptococcus oligofermentans
649990004 Streptococcus cristatus
2531839557 Streptococcus sanguinis
2529293055 Streptococcus sanguinis
2531839560 Streptococcus sanguinis
2531839564 Streptococcus sanguinis
2531839559 Streptococcus sanguinis
2531839561 Streptococcus sanguinis
2515154090 Streptococcus anginosus
649990002 Streptococcus anginosus
2529293222 Streptococcus intermedius
2541047048 Streptococcus intermedius
2515154013 Streptococcus massiliensis
2511231186 Streptococcus suis
640427146 Streptococcus suis
644736407 Streptococcus suis
2511231193 Streptococcus suis
2511231189 Streptococcus suis
2515154025 Streptococcus ovis
2519103182 Streptococcus minor
2522572191 Streptococcus plurextorum
2515154023 Streptococcus entericus
2515154010 Streptococcus merionis
UID545|f Streptococcaceae|1.000
637000298 Streptococcus pyogenes
2519103094 Streptococcus pyogenes
2523533631 Streptococcus pyogenes
643348577 Streptococcus pyogenes
637000296 Streptococcus pyogenes
2511231137 Streptococcus pyogenes
2519899549 Streptococcus pyogenes
637000291 Streptococcus pyogenes
2529292875 Streptococcus dysgalactiae
2521172714 Streptococcus dysgalactiae
2529292876 Streptococcus dysgalactiae
2528311138 Streptococcus canis
2511231157 Streptococcus equi
642555163 Streptococcus equi
643692038 Streptococcus equi
643348578 Streptococcus uberis
2515154028 Streptococcus didelphis
2529293127 Streptococcus iniae
2531839400 Streptococcus parauberis
651324102 Streptococcus parauberis
2512047073 Streptococcus ictaluri
2515154195 Streptococcus thoraltensis
2524614633 Streptococcus hyovaginalis
2529293089 Streptococcus agalactiae
2529293232 Streptococcus agalactiae
2529293090 Streptococcus agalactiae
2518285604 Streptococcus agalactiae
2534682067 Streptococcus agalactiae
2529293088 Streptococcus agalactiae
UID544|0.934
UID543|1.000

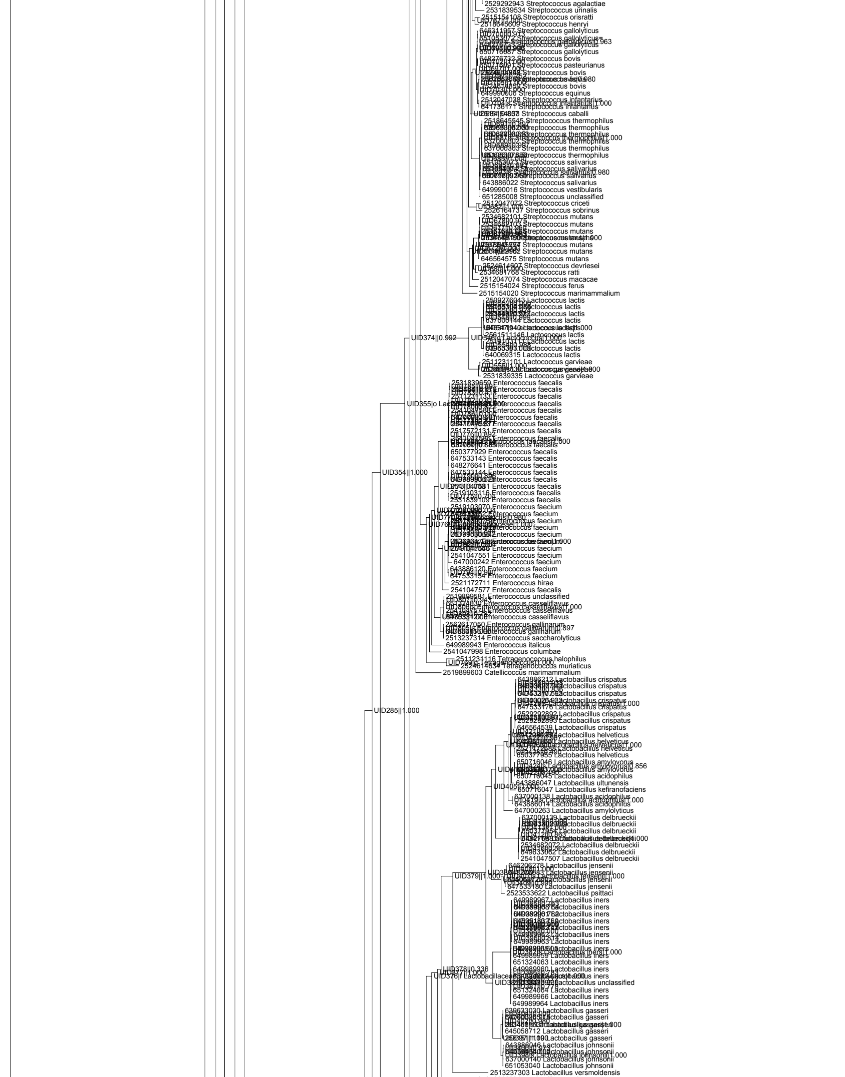
2529292943 Streptococcus agalactiae
2531839534 Streptococcus urinalis
2515154108 Streptococcus orisratti
2518645609 Streptococcus henryi
646311957 Streptococcus gallolyticus
651053072 Streptococcus gallolyticus
650716067 Streptococcus gallolyticus
648276732 Streptococcus bovis
650716091 Streptococcus pasteurianus
2524614859 Streptococcus bovis
649990006 Streptococcus equinus
2512047038 Streptococcus infantarius
641736171 Streptococcus infantarius
Streptococcus caballi
2518645545 Streptococcus thermophilus
637000302 Streptococcus thermophilus
637000303 Streptococcus thermophilus
643886022 Streptococcus salivarius
649990016 Streptococcus vestibularis
651285008 Streptococcus unclassified
2512047072 Streptococcus criceti
2526164737 Streptococcus sobrinus
2534682101 Streptococcus mutans
646564575 Streptococcus mutans
2524614607 Streptococcus devriesei
2534681768 Streptococcus ratti
2512047074 Streptococcus macacae
2515154024 Streptococcus ferus
2515154020 Streptococcus marimammalium
2509276043 Lactococcus lactis
637000144 Lactococcus lactis
2561511146 Lactococcus lactis
640069315 Lactococcus lactis
UID374||0.992
2511231101 Lactococcus garvieae
2531839335 Lactococcus garvieae
2531839659 Enterococcus faecalis
2517572131 Enterococcus faecalis
650377929 Enterococcus faecalis
647533143 Enterococcus faecalis
648276641 Enterococcus faecalis
647533144 Enterococcus faecalis
UID354||1.000
2519103116 Enterococcus faecalis
2531839109 Enterococcus faecalis
2519103070 Enterococcus faecium
2541047551 Enterococcus faecium
647000242 Enterococcus faecium
643886120 Enterococcus faecium
647533154 Enterococcus faecium
2521172711 Enterococcus hirae
2541047577 Enterococcus faecalis
2519899581 Enterococcus unclassified
651324030 Enterococcus casseliflavus
2562617050 Enterococcus gallinarum
2513237314 Enterococcus saccharolyticus
649989943 Enterococcus italicus
2541047998 Enterococcus columbae
2511231116 Tetragenococcus halophilus
2524614634 Tetragenococcus muriaticus
2519899603 Catellicoccus marimammalium
UID285||1.000
643886212 Lactobacillus crispatus
647533176 Lactobacillus crispatus
2529292892 Lactobacillus crispatus
646564539 Lactobacillus crispatus
650377955 Lactobacillus helveticus
650716046 Lactobacillus amylovorus
650716045 Lactobacillus acidophilus
643886047 Lactobacillus ultunensis
650716047 Lactobacillus kefiranofaciens
637000138 Lactobacillus acidophilus
643886014 Lactobacillus acidophilus
647000263 Lactobacillus amylolyticus
637000139 Lactobacillus delbrueckii
2534682072 Lactobacillus delbrueckii
649633062 Lactobacillus delbrueckii
2541047507 Lactobacillus delbrueckii
646206278 Lactobacillus jensenii
647533180 Lactobacillus jensenii
UID379||1.000
2523533622 Lactobacillus psittaci
649989967 Lactobacillus iners
649989963 Lactobacillus iners
649989959 Lactobacillus iners
651324063 Lactobacillus iners
649989960 Lactobacillus iners
UID378||0.336
651324064 Lactobacillus iners
649989966 Lactobacillus iners
649989964 Lactobacillus iners
639633030 Lactobacillus gasseri
650058712 Lactobacillus gasseri
643886046 Lactobacillus johnsonii
637000140 Lactobacillus johnsonii
651053040 Lactobacillus johnsonii
2513237303 Lactobacillus versmoldensis

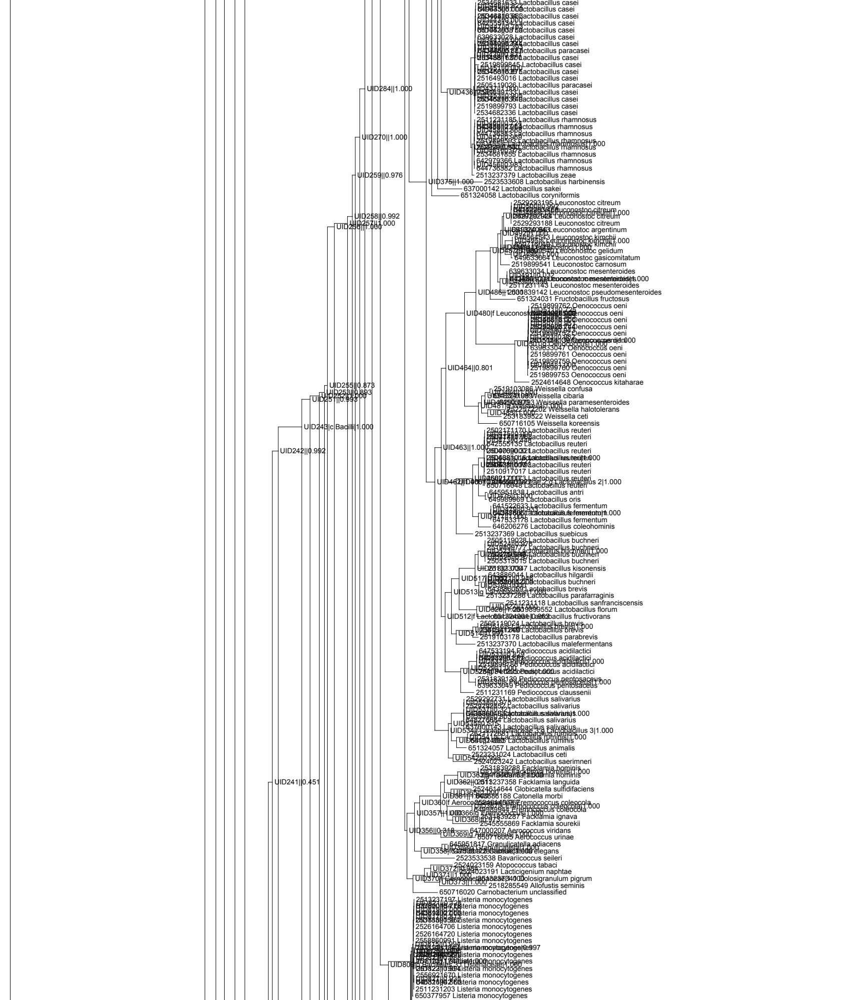

2534681633 Lactobacillus casei
Lactobacillus casei
Lactobacillus paracasei
2519899845 Lactobacillus casei
2516493016 Lactobacillus casei
2505119026 Lactobacillus paracasei
2519899793 Lactobacillus casei
2534682336 Lactobacillus casei
2511231185 Lactobacillus rhamnosus
Lactobacillus rhamnosus
2534681855 Lactobacillus rhamnosus
642979366 Lactobacillus rhamnosus
644736382 Lactobacillus rhamnosus
2513237379 Lactobacillus zeae
2523533608 Lactobacillus harbinensis
637000142 Lactobacillus sakei
651324058 Lactobacillus coryniformis
UID284||1.000
UID436
UID270||1.000
UID259||0.976
UID375||1.000
UID258||0.992
UID257||1.000
UID256||1.000
2529293195 Leuconostoc citreum
Leuconostoc citreum
2529293188 Leuconostoc citreum
Leuconostoc argentinum
645564543 Leuconostoc kimchii
Leuconostoc gelidum
649633064 Leuconostoc gasicomitatum
2519899541 Leuconostoc carnosum
639633034 Leuconostoc mesenteroides
2511231143 Leuconostoc mesenteroides
2500839142 Leuconostoc pseudomesenteroides
651324031 Fructobacillus fructosus
UID486
2519899762 Oenococcus oeni
Oenococcus oeni
639633047 Oenococcus oeni
2519899761 Oenococcus oeni
2519899759 Oenococcus oeni
2519899760 Oenococcus oeni
2519899753 Oenococcus oeni
2524614648 Oenococcus kitaharae
UID480|f Leuconostocaceae
UID464||0.801
2519103086 Weissella confusa
Weissella cibaria
Weissella paramesenteroides
Weissella halotolerans
2531839522 Weissella ceti
650716105 Weissella koreensis
UID255||0.873
UID253||0.893
UID252||1.000
UID251||0.993
UID243|c Bacilli|1.000
UID242||0.992
2502171170 Lactobacillus reuteri
Lactobacillus reuteri
642555135 Lactobacillus reuteri
2510917017 Lactobacillus reuteri
650716048 Lactobacillus reuteri
645951838 Lactobacillus antri
649989969 Lactobacillus oris
UID463||1.000
UID462
641522633 Lactobacillus fermentum
647533178 Lactobacillus fermentum
646206276 Lactobacillus coleohominis
2513237369 Lactobacillus suebicus
2505119028 Lactobacillus buchneri
Lactobacillus buchneri
2505313015 Lactobacillus buchneri
Lactobacillus kisonensis
643886044 Lactobacillus hilgardii
Lactobacillus brevis
2513237286 Lactobacillus parafarraginis
2511231118 Lactobacillus sanfranciscensis
Lactobacillus florum
Lactobacillus fructivorans
UID517
UID513
UID512
2505119024 Lactobacillus brevis
2519103178 Lactobacillus parabrevis
2513237370 Lactobacillus malefermentans
647533194 Pediococcus acidilactici
Pediococcus acidilactici
2531839139 Pediococcus pentosaceus
639633046 Pediococcus pentosaceus
2511231169 Pediococcus claussenii
2529292731 Lactobacillus salivarius
Lactobacillus salivarius
637000143 Lactobacillus salivarius
Lactobacillus ruminis
651324057 Lactobacillus animalis
2523231024 Lactobacillus ceti
2524023242 Lactobacillus saerimneri
UID534
UID535
2531839288 Facklamia hominis
2513237358 Facklamia languida
2524614644 Globicatella sulfidifaciens
Catonella morbi
Eremococcus coleocola
Facklamia ignava
2545555869 Facklamia sourekii
647000207 Aerococcus viridans
Aerococcus urinae
645951817 Granulicatella adiacens
2523533538 Bavariicoccus seileri
2524023159 Atopococcus tabaci
2524023191 Lacticigenium naphtae
Dolosigranulum pigrum
2518285549 Allofustis seminis
650716020 Carnobacterium unclassified
UID362
UID360|f Aerococcaceae
UID357
UID356||0.318
UID370
UID241||0.451
2513237197 Listeria monocytogenes
Listeria monocytogenes
2526164706 Listeria monocytogenes
2526164720 Listeria monocytogenes
2558860991 Listeria monocytogenes
2556921670 Listeria monocytogenes
2511231203 Listeria monocytogenes
650377957 Listeria monocytogenes

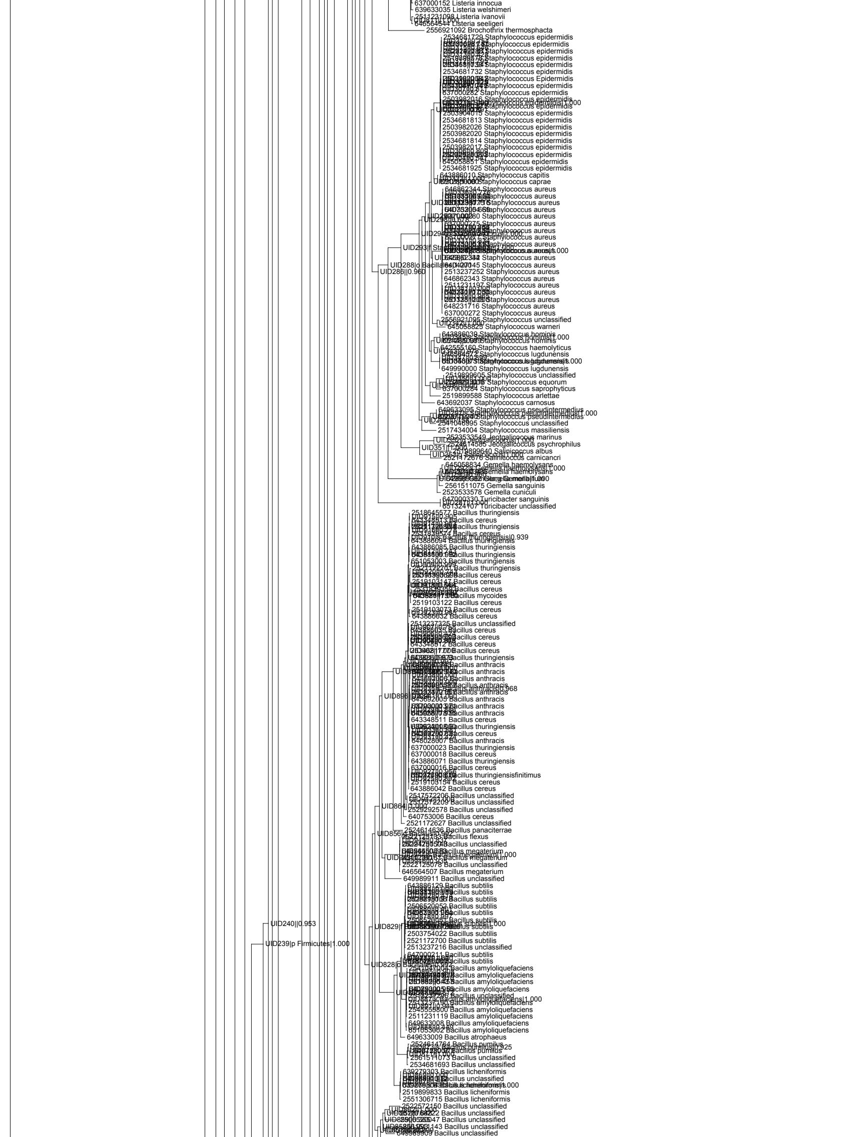
637000152 Listeria innocua
639633035 Listeria welshimeri
2511231098 Listeria ivanovii
646564544 Listeria seeligeri
2556921092 Brochothrix thermosphacta
2534681729 Staphylococcus epidermidis
2534681732 Staphylococcus epidermidis
2534681813 Staphylococcus epidermidis
2503982026 Staphylococcus epidermidis
2503982020 Staphylococcus epidermidis
2534681814 Staphylococcus epidermidis
2534681925 Staphylococcus epidermidis
643886010 Staphylococcus capitis
646862344 Staphylococcus aureus
UID293|f Sta
UID288|o Bacillales
UID286||0.960
2513237252 Staphylococcus aureus
646862343 Staphylococcus aureus
2511231197 Staphylococcus aureus
648231716 Staphylococcus aureus
637000272 Staphylococcus aureus
2556921095 Staphylococcus unclassified
645058825 Staphylococcus warneri
643886039 Staphylococcus hominis
642555160 Staphylococcus haemolyticus
649990000 Staphylococcus lugdunensis
2519899605 Staphylococcus unclassified
637000284 Staphylococcus saprophyticus
2519899588 Staphylococcus arlettae
643692037 Staphylococcus carnosus
649633095 Staphylococcus pseudintermedius
2541046995 Staphylococcus unclassified
2517434004 Staphylococcus massiliensis
2523533549 Jeotgalicoccus marinus
2524614585 Jeotgalicoccus psychrophilus
2519899640 Salinicoccus albus
2521172676 Salinicoccus carnicancri
645058834 Gemella haemolysans
2561511075 Gemella sanguinis
2523533578 Gemella cuniculi
647000330 Turicibacter sanguinis
651324107 Turicibacter unclassified
2518645577 Bacillus thuringiensis
643886094 Bacillus thuringiensis
643886085 Bacillus thuringiensis
651053003 Bacillus thuringiensis
2519103147 Bacillus cereus
2519103122 Bacillus cereus
643886032 Bacillus cereus
2513237325 Bacillus unclassified
643348512 Bacillus cereus
643692005 Bacillus anthracis
643348511 Bacillus cereus
648028007 Bacillus anthracis
637000023 Bacillus thuringiensis
637000018 Bacillus cereus
643886071 Bacillus thuringiensis
637000016 Bacillus cereus
2519103154 Bacillus cereus
643886042 Bacillus cereus
2517572206 Bacillus unclassified
2529292578 Bacillus unclassified
640753006 Bacillus cereus
2521172627 Bacillus unclassified
2524614636 Bacillus panaciterrae
2522125078 Bacillus unclassified
646564507 Bacillus megaterium
649989911 Bacillus unclassified
643886129 Bacillus subtilis
2506520052 Bacillus subtilis
UID240||0.953
UID239|p Firmicutes|1.000
2503754022 Bacillus subtilis
2521172700 Bacillus subtilis
2513237216 Bacillus unclassified
647000211 Bacillus subtilis
2541047004 Bacillus amyloliquefaciens
2545555800 Bacillus amyloliquefaciens
2511231119 Bacillus amyloliquefaciens
649633008 Bacillus amyloliquefaciens
649633009 Bacillus atrophaeus
2524614764 Bacillus pumilus
2561511073 Bacillus unclassified
2534681693 Bacillus unclassified
639279303 Bacillus licheniformis
2519899833 Bacillus licheniformis
2551306715 Bacillus licheniformis
2522572150 Bacillus unclassified
649989909 Bacillus unclassified

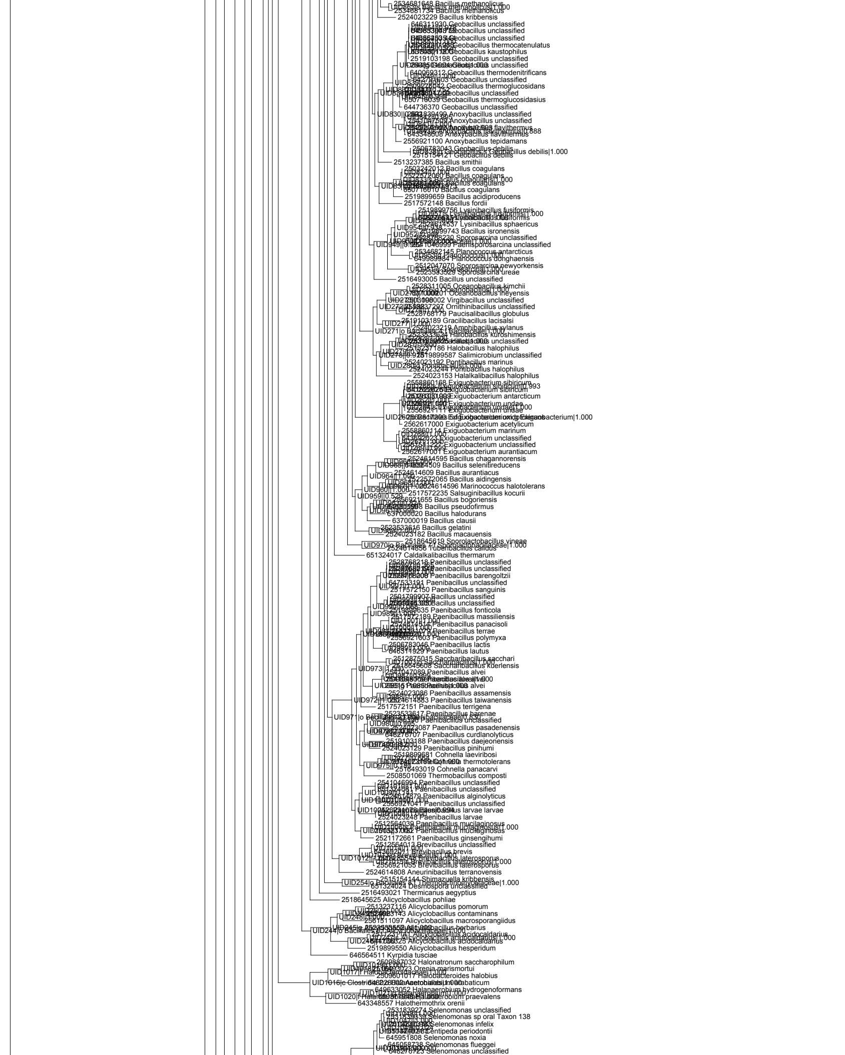

2534681648 Bacillus methanolicus
2534681734 Bacillus methanolicus
2524023229 Bacillus kribbensis
646311930 Geobacillus unclassified
Geobacillus thermocatenulatus
Geobacillus kaustophilus
2519103198 Geobacillus unclassified
640069312 Geobacillus thermodenitrificans
Geobacillus thermoglucosidans
650716039 Geobacillus thermoglucosidasius
644736370 Geobacillus unclassified
Anoxybacillus unclassified
643348508 Anoxybacillus flavithermus
2556921100 Anoxybacillus tepidamans
2506783043 Geobacillus debilis
2515154121 Geobacillus debilis
2513237385 Bacillus smithii
2503242012 Bacillus coagulans
650716010 Bacillus coagulans
2519899659 Bacillus acidiproducens
2517572148 Bacillus fordii
2519899756 Lysinibacillus fusiformis
Lysinibacillus sphaericus
2519899743 Bacillus isronensis
Sporosarcina unclassified
2541046995 Paenisporosarcina unclassified
2534682145 Planococcus antarcticus
649989984 Planococcus donghaensis
2512047070 Sporosarcina newyorkensis
2523533529 Sporosarcina ureae
2516493005 Bacillus unclassified
2528311005 Oceanobacillus kimchii
Oceanobacillus iheyensis
Virgibacillus unclassified
Ornithinibacillus unclassified
2528768179 Paucisalibacillus globulus
2519103189 Gracilibacillus lacisalsi
2524023219 Amphibacillus xylanus
Halobacillus kuroshimensis
2513237186 Halobacillus halophilus
2519899587 Salimicrobium unclassified
2524023192 Pontibacillus marinus
2524023244 Pontibacillus halophilus
2524023153 Halalkalibacillus halophilus
2558860168 Exiguobacterium sibiricum
Exiguobacterium antarcticum
Exiguobacterium undae
2562617000 Exiguobacterium acetylicum
2558860114 Exiguobacterium marinum
Exiguobacterium unclassified
Exiguobacterium aurantiacum
2524614595 Bacillus chagannorensis
Bacillus selenitireducens
2524614609 Bacillus aurantiacus
2522572065 Bacillus aidingensis
2524614596 Marinococcus halotolerans
2517572235 Salsuginibacillus kocurii
2556921655 Bacillus bogoriensis
Bacillus pseudofirmus
637000020 Bacillus halodurans
637000019 Bacillus clausii
2523533616 Bacillus gelatini
2524023182 Bacillus macauensis
2518645619 Sporolactobacillus vineae
2524614856 Tuberibacillus calidus
651324017 Caldalkalibacillus thermarum
2528768218 Paenibacillus unclassified
Paenibacillus barengoltzii
647533191 Paenibacillus unclassified
2517572150 Paenibacillus sanguinis
2501799907 Bacillus unclassified
2519899635 Paenibacillus fonticola
2517572189 Paenibacillus massiliensis
Paenibacillus panacisoli
Paenibacillus terrae
2556921003 Paenibacillus polymyxa
2506783046 Paenibacillus lactis
646311929 Paenibacillus lautus
2512875015 Saccharibacillus sacchari
2518645608 Saccharibacillus kuerlensis
2541047089 Paenibacillus alvei
2524023086 Paenibacillus assamensis
Paenibacillus taiwanensis
2517572151 Paenibacillus terrigena
2523533617 Paenibacillus barenae
2524023087 Paenibacillus pasadenensis
646276707 Paenibacillus curdlanolyticus
2519103188 Paenibacillus daejeonensis
2524023129 Paenibacillus pinihumi
2519899681 Cohnella laeviribosi
2516493019 Cohnella panacarvi
2508501069 Thermobacillus composti
2541046994 Paenibacillus unclassified
Paenibacillus alginolyticus
2556921041 Paenibacillus unclassified
2524023248 Paenibacillus larvae
2512564039 Paenibacillus mucilaginosus
2521172661 Paenibacillus ginsengihumi
2512564013 Brevibacillus unclassified
Brevibacillus brevis
2556921055 Brevibacillus laterosporus
2524614808 Aneurinibacillus terranovensis
2515154144 Shimazuella kribbensis
651324024 Desmospora unclassified
2516493021 Thermicanus aegyptius
2518645625 Alicyclobacillus pohliae
2513237116 Alicyclobacillus pomorum
Alicyclobacillus contaminans
2561511097 Alicyclobacillus macrosporangiidus
Alicyclobacillus herbarius
Alicyclobacillus acidocaldarius
2519899550 Alicyclobacillus hesperidum
646564511 Kyrpidia tusciae
2509887032 Halonatronum saccharophilum
Orenia marismortui
2509601017 Halobacteroides halobius
649633052 Halanaerobium hydrogeniformans
Halanaerobium praevalens
643348557 Halothermothrix orenii
2531839274 Selenomonas unclassified
Selenomonas sp oral Taxon 138
Selenomonas infelix
Centipeda periodontii
645951808 Selenomonas noxia
645058738 Selenomonas flueggei
646276723 Selenomonas unclassified

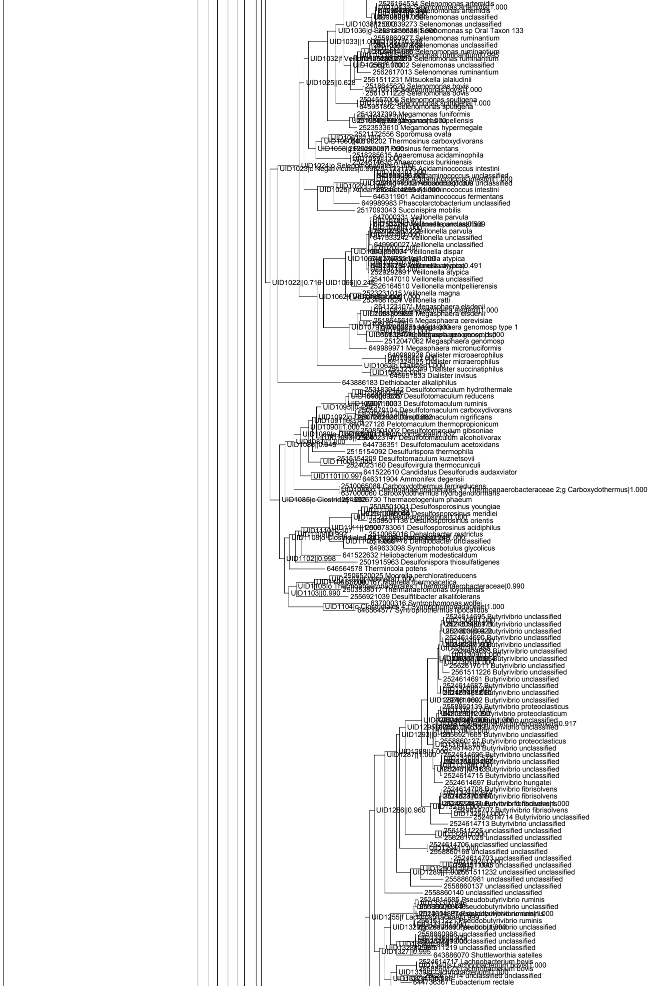

2526164534 Selenomonas artemidis
2558860977 Selenomonas ruminantium
2562617013 Selenomonas ruminantium
2561511231 Mitsuokella jalaludinii
2518645620 Selenomonas bovis
2561511229 Selenomonas bovis
2504557006 Selenomonas sputigena
645951802 Selenomonas sputigena
2513237399 Megamonas funiformis
2523533610 Megamonas hypermegale
2521172556 Sporomusa ovata
2518285615 Anaeromusa acidaminophila
2524614635 Anaeroarcus burkinensis
646311901 Acidaminococcus fermentans
649989983 Phascolarctobacterium unclassified
2517093043 Succinispira mobilis
UID1023|c Negativicutes|0.998
UID1025||0.628
647000331 Veillonella parvula
647533242 Veillonella unclassified
649990027 Veillonella unclassified
2529292891 Veillonella atypica
2541047010 Veillonella unclassified
2526164510 Veillonella montpellierensis
2523231015 Veillonella magna
2534681624 Veillonella ratti
2511231071 Megasphaera elsdenii
2518645616 Megasphaera cerevisiae
2512047062 Megasphaera genomosp
649989971 Megasphaera micronuciformis
649989928 Dialister microaerophilus
645951833 Dialister invisus
UID1022||0.710
UID1066||0.245
643886183 Dethiobacter alkaliphilus
2531839442 Desulfotomaculum hydrothermale
2515679104 Desulfotomaculum carboxydivorans
2510427128 Pelotomaculum thermopropionicum
2508501002 Desulfotomaculum gibsoniae
644736351 Desulfotomaculum acetoxidans
2515154092 Desulfurispora thermophila
2515154209 Desulfotomaculum kuznetsovii
2524023160 Desulfovirgula thermocuniculi
UID1101||0.997
641522610 Candidatus Desulforudis audaxviator
646311904 Ammonifex degensii
2510065088 Carboxydothermus ferrireducens
637000060 Carboxydothermus hydrogenoformans
2506783061 Desulfosporosinus acidiphilus
2510065016 Dehalobacter restrictus
649633098 Syntrophobotulus glycolicus
UID1102||0.998
641522632 Heliobacterium modesticaldum
2501915963 Desulfonispora thiosulfatigenes
646564578 Thermincola potens
2506520025 Moorella perchloratireducens
UID1103||0.990
2556921039 Desulfitibacter alkalitolerans
637000316 Syntrophomonas wolfei
646564577 Syntrophothermus lipocalidus
2524614695 Butyrivibrio unclassified
2524614690 Butyrivibrio unclassified
2562617011 Butyrivibrio unclassified
2561511226 Butyrivibrio unclassified
2524614691 Butyrivibrio unclassified
2524614687 Butyrivibrio unclassified
2558860139 Butyrivibrio proteoclasticus
2556921665 Butyrivibrio unclassified
2558860127 Butyrivibrio proteoclasticus
2524614570 Butyrivibrio unclassified
2524614715 Butyrivibrio unclassified
2524614697 Butyrivibrio hungatei
2524614708 Butyrivibrio fibrisolvens
2524614707 Butyrivibrio fibrisolvens
2524614714 Butyrivibrio unclassified
2524614713 Butyrivibrio unclassified
UID1286||0.960
UID1287||1.000
2561511225 unclassified unclassified
2562617029 unclassified unclassified
2524614706 unclassified unclassified
2558860166 unclassified unclassified
2524614703 unclassified unclassified
2561511232 unclassified unclassified
2558860981 unclassified unclassified
2558860137 unclassified unclassified
2558860140 unclassified unclassified
2524614685 Pseudobutyrivibrio ruminis
2558860988 unclassified unclassified
2561511219 unclassified unclassified
643886070 Shuttleworthia satelles
2524614717 Lachnobacterium bovis
644736367 Eubacterium rectale

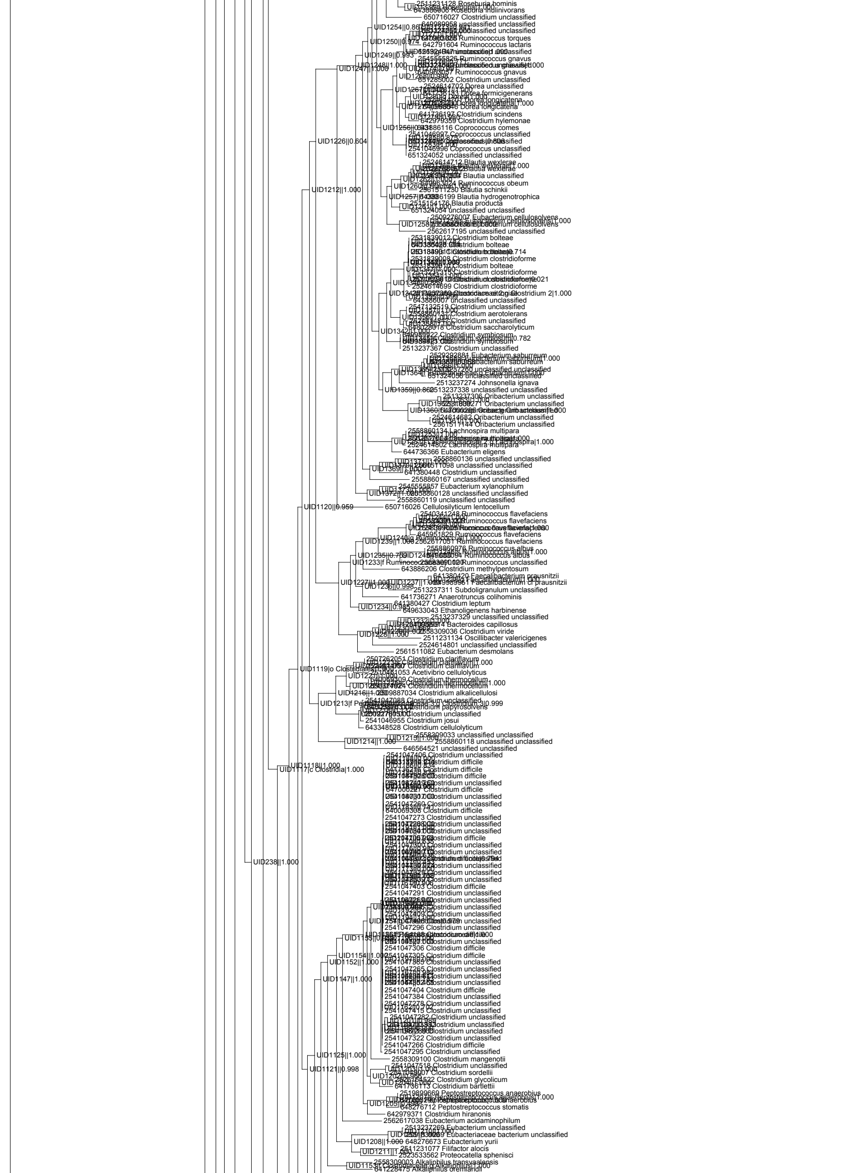

2511231128 Roseburia hominis
643886006 Roseburia inulinivorans
650716027 Clostridium unclassified
649989958 unclassified unclassified
UID1254||0.867
UID1250||0.974
642791604 Ruminococcus lactaris
UID1249||0.993
UID1248||1.000
UID1247||1.000
651285002 Clostridium unclassified
2524614702 Dorea unclassified
641736197 Clostridium scindens
642979359 Clostridium hylemonae
UID1226||0.604
2541046997 Coprococcus unclassified
2541046996 Coprococcus unclassified
651324052 unclassified unclassified
2524614712 Blautia wexlerae
650963024 Ruminococcus obeum
2561511230 Blautia schinkii
UID1212||1.000
2515154176 Blautia producta
651324054 unclassified unclassified
2562617195 unclassified unclassified
2531839012 Clostridium bolteae
2531839008 Clostridium clostridioforme
2524614699 Clostridium clostridioforme
643886007 unclassified unclassified
2547132519 Clostridium unclassified
640028018 Clostridium saccharolyticum
2513237367 Clostridium unclassified
2529292881 Eubacterium saburreum
2513237274 Johnsonella ignava
UID1359||0.862
2513237338 unclassified unclassified
2513237306 Oribacterium unclassified
2524614682 Oribacterium unclassified
2561511144 Oribacterium unclassified
2558860134 Lachnospira multipara
2524614802 Lachnospira multipara
644736366 Eubacterium eligens
2558860136 unclassified unclassified
641380448 Clostridium unclassified
2558860167 unclassified unclassified
2545555857 Eubacterium xylanophilum
2558860119 unclassified unclassified
UID1120||0.959
650716026 Cellulosilyticum lentocellum
2540341248 Ruminococcus flavefaciens
645951829 Ruminococcus flavefaciens
2562617051 Ruminococcus flavefaciens
2558860976 Ruminococcus albus
643886206 Clostridium methylpentosum
641380420 Faecalibacterium prausnitzii
2513237311 Subdoligranulum unclassified
641736271 Anaerotruncus colihominis
641380427 Clostridium leptum
UID1234||0.985
649633043 Ethanoligenens harbinense
2513237329 unclassified unclassified
2511231134 Oscillibacter valericigenes
2524614801 unclassified unclassified
2561511082 Eubacterium desmolans
2507262051 Clostridium clariflavum
UID1119|o Clostridiales
Acetivibrio cellulolyticus
UID1216||1.000
Clostridium alkalicellulosi
UID1213|f
2541047088 Clostridium unclassified
2541046955 Clostridium josui
643348528 Clostridium cellulolyticum
2558309033 unclassified unclassified
UID1214||1.000
2558860118 unclassified unclassified
646564521 unclassified unclassified
UID1118||1.000
UID1117|c Clostridia|1.000
2541047406 Clostridium unclassified
647000227 Clostridium difficile
640069308 Clostridium difficile
2541047273 Clostridium unclassified
UID238||1.000
2541047403 Clostridium difficile
2541047291 Clostridium unclassified
2541047296 Clostridium unclassified
2541047306 Clostridium difficile
UID1147||1.000
2541047404 Clostridium difficile
2541047384 Clostridium unclassified
2541047415 Clostridium unclassified
2541047282 Clostridium unclassified
2541047322 Clostridium unclassified
2541047266 Clostridium difficile
2541047295 Clostridium unclassified
UID1125||1.000
2558309100 Clostridium mangenotii
UID1121||0.998
2541047518 Clostridium unclassified
Clostridium sordellii
Clostridium glycolicum
641736113 Clostridium bartlettii
2519899669 Peptostreptococcus anaerobius
648276712 Peptostreptococcus stomatis
642979371 Clostridium hiranonis
2562617038 Eubacterium acidaminophilum
2513237269 Eubacterium unclassified
Eubacteriaceae bacterium unclassified
UID1208||1.000
648276673 Eubacterium yurii
2511231077 Filifactor alocis
2523533562 Proteocatella sphenisci
2558309003 Alkaliphilus transvaalensis

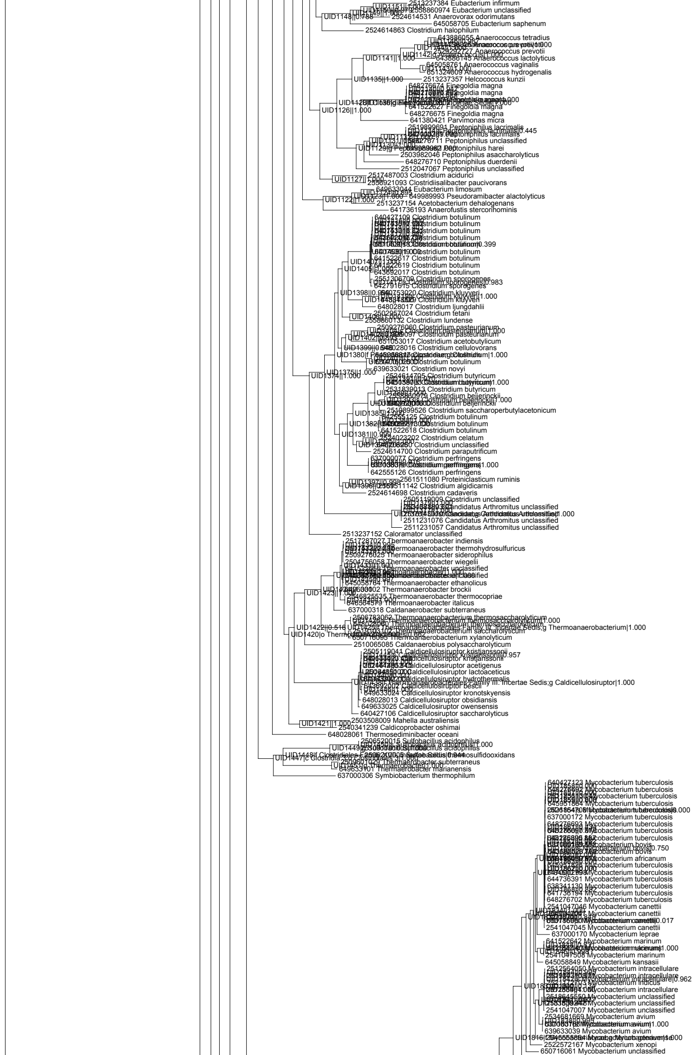

2513237384 Eubacterium infirmum
2558860974 Eubacterium unclassified
2524614531 Anaerovorax odorimutans
645058705 Eubacterium saphenum
2524614863 Clostridium halophilum
643886055 Anaerococcus tetradius
2529292727 Anaerococcus prevotii
643886145 Anaerococcus lactolyticus
645058761 Anaerococcus vaginalis
651324009 Anaerococcus hydrogenalis
2513237357 Helcococcus kunzii
648276674 Finegoldia magna
641522627 Finegoldia magna
648276675 Finegoldia magna
641380421 Parvimonas micra
2519899691 Peptoniphilus lacrimalis
648276711 Peptoniphilus unclassified
2503982046 Peptoniphilus asaccharolyticus
648276710 Peptoniphilus duerdenii
2512047067 Peptoniphilus unclassified
2517487003 Clostridium acidurici
2556921093 Clostridiisalibacter paucivorans
649633044 Eubacterium limosum
649989993 Pseudoramibacter alactolyticus
2513237154 Acetobacterium dehalogenans
641736193 Anaerofustis stercorihominis
640427109 Clostridium botulinum
641522617 Clostridium botulinum
641522619 Clostridium botulinum
643692017 Clostridium botulinum
2551306709 Clostridium sporogenes
642791615 Clostridium sporogenes
640753020 Clostridium kluyveri
648028017 Clostridium ljungdahlii
2502957024 Clostridium tetani
2558860132 Clostridium lundense
2509276060 Clostridium pasteurianum
651053017 Clostridium acetobutylicum
648028016 Clostridium cellulovorans
639633021 Clostridium novyi
2524614705 Clostridium butyricum
2531839013 Clostridium butyricum
2519899526 Clostridium saccharoperbutylacetonicum
642555125 Clostridium botulinum
641522618 Clostridium botulinum
2524023202 Clostridium celatum
2524614700 Clostridium paraputrificum
637000077 Clostridium perfringens
642555126 Clostridium perfringens
2561511080 Proteiniclasticum ruminis
2524614698 Clostridium cadaveris
2505119009 Clostridium unclassified
2511231076 Candidatus Arthromitus unclassified
2511231057 Candidatus Arthromitus unclassified
2513237152 Caloramator unclassified
2517287027 Thermoanaerobacter indiensis
2509276025 Thermoanaerobacter siderophilus
2504756058 Thermoanaerobacter wiegelii
645058764 Thermoanaerobacter ethanolicus
2546825535 Thermoanaerobacter thermocopriae
646564579 Thermoanaerobacter italicus
637000318 Caldanaerobacter subterraneus
2506783062 Thermoanaerobacterium thermosaccharolyticum
650716095 Thermoanaerobacterium xylanolyticum
2510065085 Caldanaerobius polysaccharolyticus
2505119041 Caldicellulosiruptor kristjanssonii
649633024 Caldicellulosiruptor kronotskyensis
648028013 Caldicellulosiruptor obsidiansis
649633025 Caldicellulosiruptor owensensis
640427106 Caldicellulosiruptor saccharolyticus
2503508009 Mahella australiensis
2540341239 Caldicoprobacter oshimai
648028061 Thermosediminibacter oceani
2506520015 Sulfobacillus acidophilus
637000306 Symbiobacterium thermophilum
640427123 Mycobacterium tuberculosis
637000172 Mycobacterium tuberculosis
648276693 Mycobacterium tuberculosis
644736391 Mycobacterium tuberculosis
638341130 Mycobacterium tuberculosis
648276702 Mycobacterium tuberculosis
2541047046 Mycobacterium canettii
2541047045 Mycobacterium canettii
637000170 Mycobacterium leprae
641522642 Mycobacterium marinum
2541047508 Mycobacterium marinum
645058849 Mycobacterium kansasii
2512564050 Mycobacterium intracellulare
2541047007 Mycobacterium unclassified
2534681669 Mycobacterium avium
639633039 Mycobacterium avium
2522572167 Mycobacterium xenopi
650716061 Mycobacterium unclassified

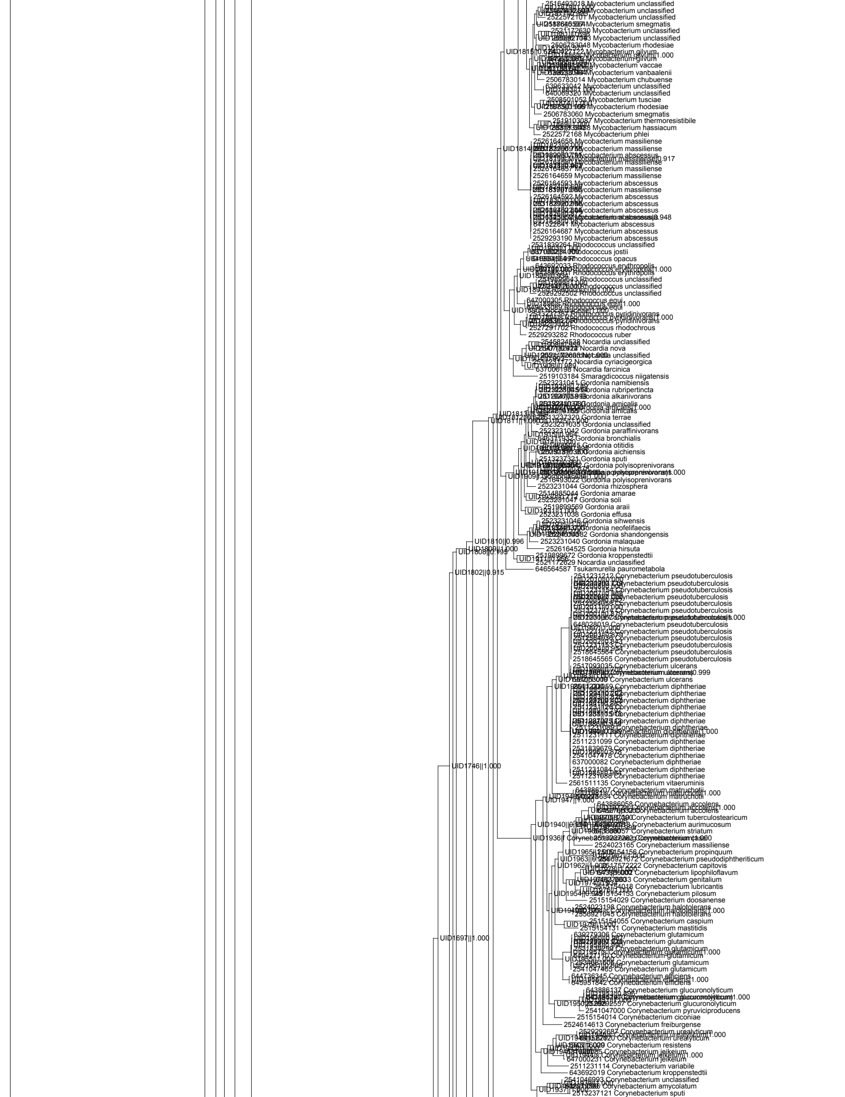

2516493018 Mycobacterium unclassified
2522572101 Mycobacterium unclassified
2521172630 Mycobacterium unclassified
2506783048 Mycobacterium rhodesiae
2506783014 Mycobacterium chubuense
639633042 Mycobacterium unclassified
640069320 Mycobacterium unclassified
2508501052 Mycobacterium tusciae
2506783060 Mycobacterium smegmatis
2519103087 Mycobacterium thermoresistibile
2522572168 Mycobacterium phlei
2526164658 Mycobacterium massiliense
2526164659 Mycobacterium massiliense
2526164687 Mycobacterium abscessus
2529293190 Mycobacterium abscessus
2531839264 Rhodococcus unclassified
2529293282 Rhodococcus ruber
2546824538 Nocardia unclassified
637000198 Nocardia farcinica
2519103184 Smaragdicoccus niigatensis
2523231041 Gordonia namibiensis
2513237320 Gordonia terrae
2523231035 Gordonia unclassified
2523231042 Gordonia paraffinivorans
2513237321 Gordonia sputi
2516493022 Gordonia polyisoprenivorans
2523231044 Gordonia rhizosphera
2514885044 Gordonia amarae
2523231047 Gordonia soli
2519899569 Gordonia araii
2523231038 Gordonia effusa
2523231046 Gordonia sihwensis
2523231040 Gordonia malaquae
2526164525 Gordonia hirsuta
UID1810||0.996
2519899672 Gordonia kroppenstedtii
2521172629 Nocardia unclassified
UID1802||0.915
646564587 Tsukamurella paurometabola
2511231212 Corynebacterium pseudotuberculosis
648028019 Corynebacterium pseudotuberculosis
2518645565 Corynebacterium pseudotuberculosis
2517093035 Corynebacterium ulcerans
2511231099 Corynebacterium diphtheriae
637000082 Corynebacterium diphtheriae
UID1746||1.000
2561511135 Corynebacterium vitaeruminis
643886058 Corynebacterium accolens
2524023165 Corynebacterium massiliense
2515154029 Corynebacterium doosanense
2515154055 Corynebacterium caspium
2519154131 Corynebacterium mastitidis
UID1697||1.000
639279306 Corynebacterium glutamicum
2541047465 Corynebacterium glutamicum
644736345 Corynebacterium efficiens
643886137 Corynebacterium glucuronolyticum
2541047000 Corynebacterium pyruviciproducens
2515154014 Corynebacterium ciconiae
2524614613 Corynebacterium freiburgense
2529292687 Corynebacterium urealyticum
647000231 Corynebacterium jeikeium
2511231114 Corynebacterium variabile
643692019 Corynebacterium kroppenstedtii
2541046993 Corynebacterium unclassified
2513237121 Corynebacterium sputi

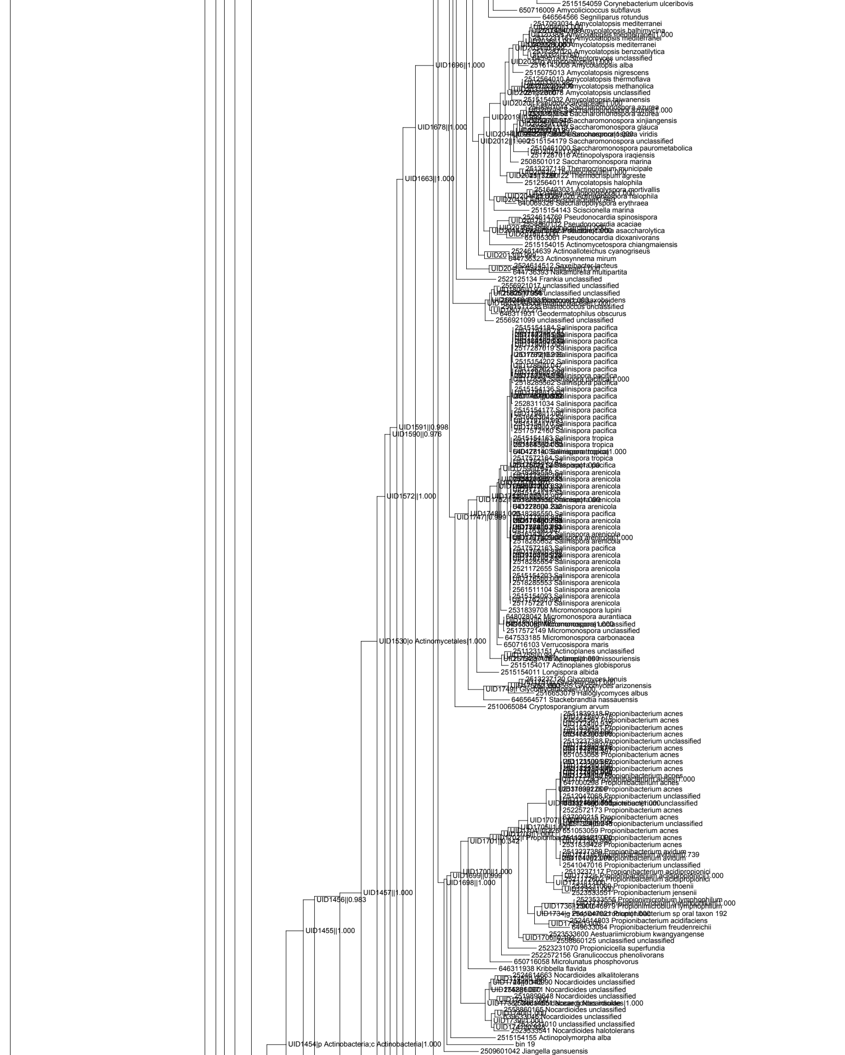

2515154059 Corynebacterium ulceribovis
650716009 Amycolicicoccus subflavus
646564566 Segniliparus rotundus
2517093034 Amycolatopsis mediterranei
2517287020 Amycolatopsis benzoatilytica
2516143008 Amycolatopsis alba
2515075013 Amycolatopsis nigrescens
2512564010 Amycolatopsis thermoflava
2515154032 Amycolatopsis taiwanensis
UID1696||1.000
UID1678||1.000
UID1663||1.000
2515154179 Saccharomonospora unclassified
2510461000 Saccharomonospora paurometabolica
2517287016 Actinopolyspora iraqiensis
2508501012 Saccharomonospora marina
2513237119 Thermocrispum municipale
2512564011 Amycolatopsis halophila
640069329 Saccharopolyspora erythraea
2515154143 Sciscionella marina
2524614769 Pseudonocardia spinosispora
651053061 Pseudonocardia dioxanivorans
2515154015 Actinomycetospora chiangmaiensis
2524614639 Actinoalloteichus cyanogriseus
644736323 Actinosynnema mirum
2524614512 Saxeibacter lacteus
644736393 Nakamurella multipartita
2522125134 Frankia unclassified
2556921017 unclassified unclassified
646311931 Geodermatophilus obscurus
2556921099 unclassified unclassified
2515154184 Salinispora pacifica
2517287019 Salinispora pacifica
2515154202 Salinispora pacifica
2518285562 Salinispora pacifica
2515154136 Salinispora pacifica
2528311034 Salinispora pacifica
2515154177 Salinispora pacifica
2517572160 Salinispora pacifica
UID1591||0.998
UID1590||0.976
2515154163 Salinispora tropica
2517572164 Salinispora tropica
UID1572||1.000
2518285550 Salinispora pacifica
2517572163 Salinispora pacifica
2518285554 Salinispora arenicola
2521172655 Salinispora arenicola
2518285553 Salinispora arenicola
2561511104 Salinispora arenicola
2517572210 Salinispora arenicola
2531839708 Micromonospora lupini
648028042 Micromonospora aurantiaca
2517572149 Micromonospora unclassified
647533185 Micromonospora carbonacea
650716103 Verrucosispora maris
UID1530|o Actinomycetales|1.000
2511231151 Actinoplanes unclassified
2515154017 Actinoplanes globisporus
2515154011 Longispora albida
2513237120 Glycomyces tenuis
2516653079 Haloglycomyces albus
646564571 Stackebrandtia nassauensis
2510065084 Cryptosporangium arvum
2513237388 Propionibacterium unclassified
651053058 Propionibacterium acnes
647000298 Propionibacterium acnes
2512047068 Propionibacterium unclassified
2522572173 Propionibacterium acnes
651053059 Propionibacterium acnes
2531839428 Propionibacterium acnes
2513237389 Propionibacterium avidum
2541047016 Propionibacterium unclassified
2513237117 Propionibacterium acidipropionici
2523533551 Propionibacterium jensenii
2524614803 Propionibacterium acidifaciens
649633084 Propionibacterium freudenreichii
2523533600 Aestuariimicrobium kwangyangense
2558860125 unclassified unclassified
2523231070 Propionicicella superfundia
2522572156 Granulicoccus phenolivorans
650716058 Microlunatus phosphovorus
646311938 Kribbella flavida
UID1457||1.000
UID1456||0.983
UID1455||1.000
UID1701||0.342
UID1700||1.000
UID1699||0.999
UID1698||1.000
2524614663 Nocardioides alkalitolerans
2519899648 Nocardioides unclassified
2558860165 Nocardioides unclassified
639633045 Nocardioides unclassified
2523533541 Nocardioides halotolerans
2515154155 Actinopolymorpha alba
bin 19
UID1454|p Actinobacteria;c Actinobacteria|1.000
2509601042 Jiangella gansuensis

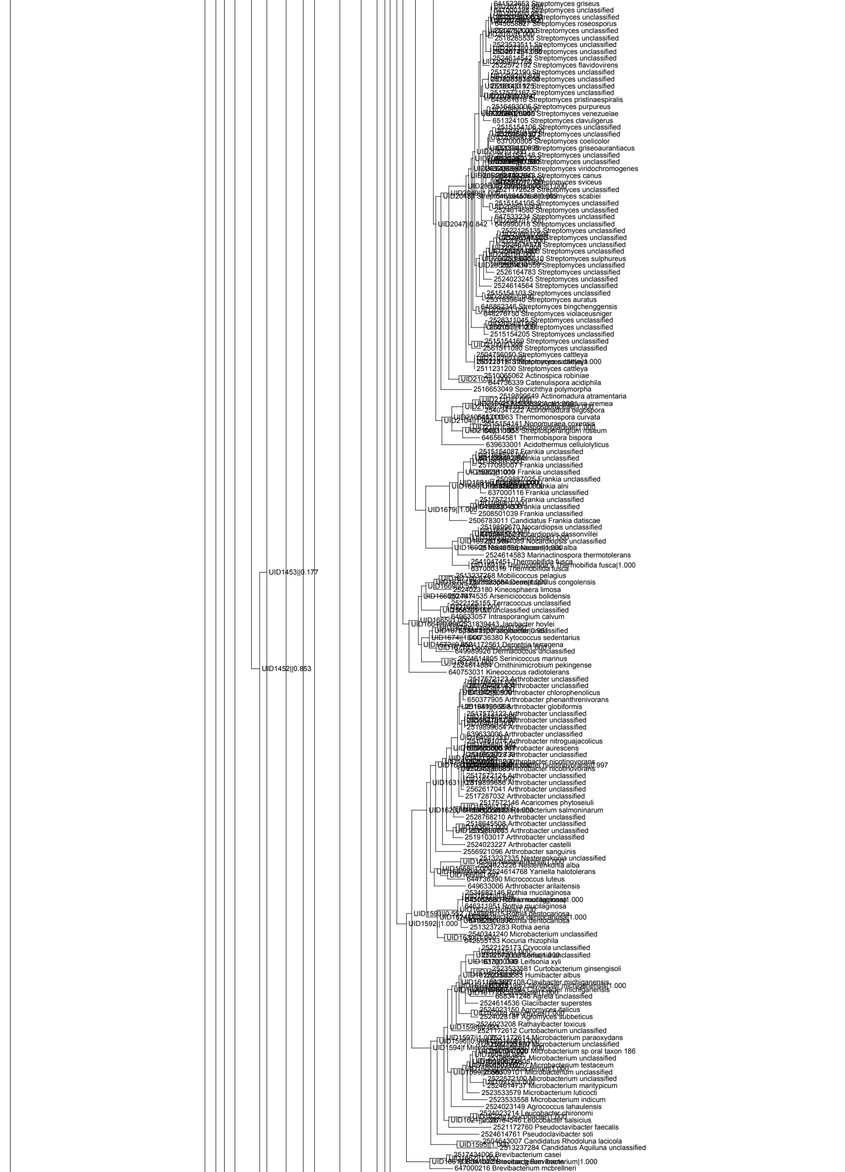
641522653 Streptomyces griseus
645058827 Streptomyces roseosporus
2518285535 Streptomyces unclassified
2523533511 Streptomyces unclassified
2524614542 Streptomyces unclassified
2522572192 Streptomyces flavidovirens
2517572190 Streptomyces unclassified
2517572167 Streptomyces unclassified
648861016 Streptomyces pristinaespiralis
2516493006 Streptomyces purpureus
651324105 Streptomyces clavuligerus
2515154106 Streptomyces unclassified
637000305 Streptomyces coelicolor
2515154148 Streptomyces unclassified
2521172628 Streptomyces unclassified
UID2049||1.000
2515154105 Streptomyces unclassified
2524614580 Streptomyces unclassified
647533234 Streptomyces unclassified
649990016 Streptomyces unclassified
UID2047||0.842
2522125135 Streptomyces unclassified
2526164783 Streptomyces unclassified
2524023245 Streptomyces unclassified
2524614564 Streptomyces unclassified
2515154103 Streptomyces unclassified
2531839540 Streptomyces auratus
646862346 Streptomyces bingchenggensis
648276750 Streptomyces violaceusniger
2528311045 Streptomyces unclassified
2515154205 Streptomyces unclassified
2515154169 Streptomyces unclassified
2561511090 Streptomyces unclassified
2504756050 Streptomyces cattleya
2511231200 Streptomyces cattleya
2510065062 Actinospica robiniae
644736339 Catenulispora acidiphila
2516653049 Sporichthya polymorpha
2519899649 Actinomadura atramentaria
2540341222 Actinomadura oligospora
2515154141 Nonomuraea coxensis
646564581 Thermobispora bispora
639633001 Acidothermus cellulolyticus
2515154087 Frankia unclassified
2517093007 Frankia unclassified
2509887025 Frankia unclassified
637000116 Frankia unclassified
2517572101 Frankia unclassified
2508501039 Frankia unclassified
UID1679||1.000
2506783011 Candidatus Frankia datiscae
2519899670 Nocardiopsis unclassified
2524614583 Marinactinospora thermotolerans
2541047451 Thermobifida fusca
637000319 Thermobifida fusca
UID1453||0.177
2513237258 Mobilicoccus pelagius
2524023180 Kineosphaera limosa
2524614535 Arsenicicoccus bolidensis
2522125155 Terracoccus unclassified
649633057 Intrasporangium calvum
2531839443 Janibacter hoylei
644736380 Kytococcus sedentarius
2521172561 Demetria terragena
649989926 Dermacoccus unclassified
2524614805 Serinicoccus marinus
2524614884 Ornithinimicrobium pekingense
640753031 Kineococcus radiotolerans
UID1452||0.853
2517572123 Arthrobacter unclassified
650377905 Arthrobacter phenanthrenivorans
2517572122 Arthrobacter unclassified
2519899654 Arthrobacter unclassified
639633006 Arthrobacter unclassified
2517572124 Arthrobacter unclassified
2562617041 Arthrobacter unclassified
2517287032 Arthrobacter unclassified
2517572146 Acaricomes phytoseiuli
2528768210 Arthrobacter unclassified
2518645508 Arthrobacter unclassified
2519103017 Arthrobacter unclassified
2524023227 Arthrobacter castelli
2556921096 Arthrobacter sanguinis
2513237335 Nesterenkonia unclassified
2524614768 Yaniella halotolerans
644736390 Micrococcus luteus
649633006 Arthrobacter arilaitensis
2534682146 Rothia mucilaginosa
646311951 Rothia mucilaginosa
2513237283 Rothia aeria
UID1592||1.000
2540341240 Microbacterium unclassified
642555133 Kocuria rhizophila
2522125173 Cryocola unclassified
2523533581 Curtobacterium ginsengisoli
658341246 Agreia unclassified
2524614536 Glaciibacter superstes
2524023150 Agromyces italicus
2524023187 Agromyces subbeticus
2524023208 Rathayibacter toxicus
2521172612 Curtobacterium unclassified
2521172614 Microbacterium paraoxydans
2522572100 Microbacterium unclassified
2524614737 Microbacterium maritypicum
2523533579 Microbacterium luticocti
2523533558 Microbacterium indicum
2524023149 Agrococcus lahaulensis
2524023214 Leucobacter chironomi
2521172760 Pseudoclavibacter faecalis
2524614761 Pseudoclavibacter soli
2504643007 Candidatus Rhodoluna lacicola
UID1595||1.000
2513237284 Candidatus Aquiluna unclassified
2517434006 Brevibacterium casei
647000216 Brevibacterium mcbrellneri

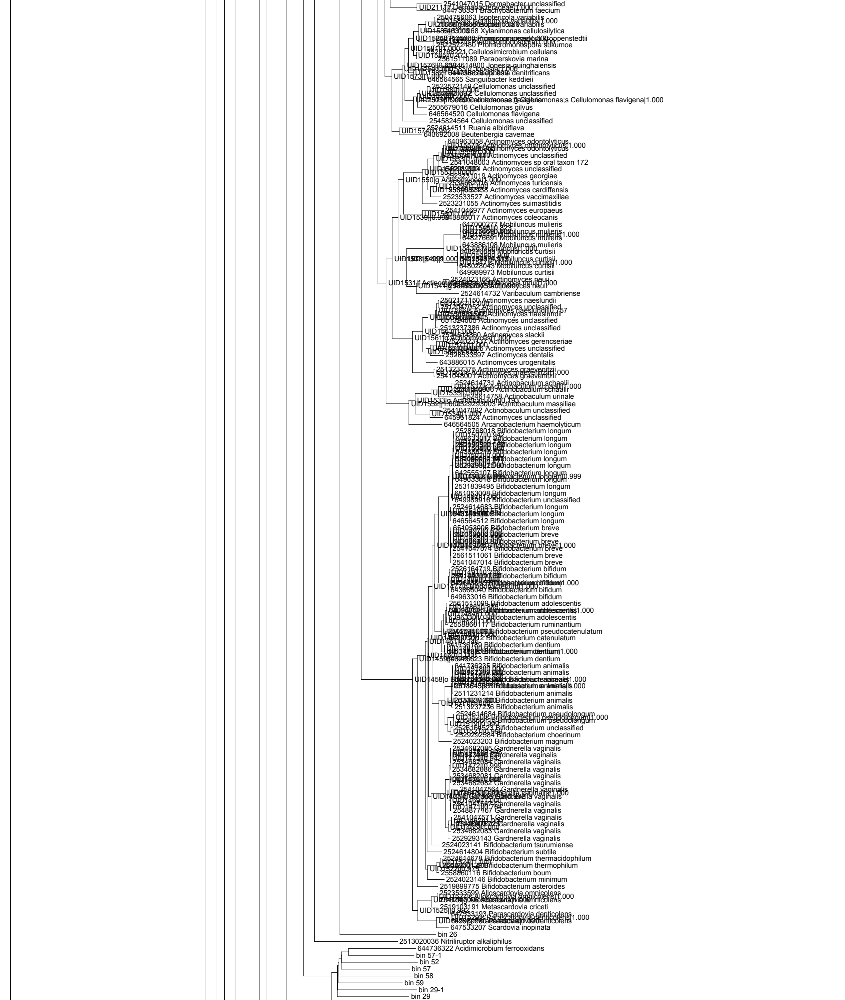

2541047015 Dermabacter unclassified
644736331 Brachybacterium faecium
2504756063 Isoptericola variabilis
646311968 Xylanimonas cellulosilytica
2522572180 Promicromonospora sukumoe
2528768221 Cellulosimicrobium cellulans
2561511089 Paraoerskovia marina
2524614800 Jonesia quinghaiensis
646564565 Sanguibacter keddieii
2522572149 Cellulomonas unclassified
Cellulomonas unclassified
2505679016 Cellulomonas gilvus
646564520 Cellulomonas flavigena
2545824564 Cellulomonas unclassified
2524614511 Ruania albidiflava
643692008 Beutenbergia cavernae
640963058 Actinomyces odontolyticus
Actinomyces unclassified
2541048003 Actinomyces sp oral taxon 172
Actinomyces unclassified
2523231019 Actinomyces georgiae
Actinomyces turicensis
Actinomyces cardiffensis
2523533527 Actinomyces vaccimaxillae
2523231055 Actinomyces suimastitidis
2541046977 Actinomyces europaeus
643886017 Actinomyces coleocanis
647000277 Mobiluncus mulieris
648276691 Mobiluncus mulieris
643886108 Mobiluncus mulieris
Mobiluncus curtisii
648028043 Mobiluncus curtisii
649989973 Mobiluncus curtisii
2524023166 Actinomyces neuii
2524614732 Varibaculum cambriense
2502171150 Actinomyces naeslundii
Actinomyces unclassified
651324005 Actinomyces unclassified
2513237386 Actinomyces unclassified
Actinomyces slackii
Actinomyces gerencseriae
Actinomyces unclassified
2528533597 Actinomyces dentalis
643886015 Actinomyces urogenitalis
2513237375 Actinomyces graevenitzii
2541046001 Actinomyces graevenitzii
2524614731 Actinobaculum schaalii
2524614758 Actinobaculum urinale
Actinobaculum massiliae
2541047092 Actinobaculum unclassified
645951824 Actinomyces unclassified
646564505 Arcanobacterium haemolyticum
2528768018 Bifidobacterium longum
642555107 Bifidobacterium longum
2531839495 Bifidobacterium longum
651053008 Bifidobacterium longum
649989916 Bifidobacterium unclassified
2524614683 Bifidobacterium longum
646564512 Bifidobacterium longum
651053005 Bifidobacterium breve
2561511061 Bifidobacterium breve
2541047014 Bifidobacterium breve
2526164719 Bifidobacterium bifidum
643886040 Bifidobacterium bifidum
649633016 Bifidobacterium bifidum
2561511099 Bifidobacterium adolescentis
Bifidobacterium adolescentis
2558860117 Bifidobacterium ruminantium
Bifidobacterium pseudocatenulatum
Bifidobacterium catenulatum
Bifidobacterium dentium
Bifidobacterium dentium
641736235 Bifidobacterium animalis
2511231214 Bifidobacterium animalis
Bifidobacterium animalis
2513237236 Bifidobacterium animalis
2524614684 Bifidobacterium pseudolongum
Bifidobacterium unclassified
Bifidobacterium choerinum
2524023203 Bifidobacterium magnum
2534682085 Gardnerella vaginalis
2534682086 Gardnerella vaginalis
2534682081 Gardnerella vaginalis
2541047564 Gardnerella vaginalis
2548877167 Gardnerella vaginalis
2541047571 Gardnerella vaginalis
2534682083 Gardnerella vaginalis
2529293143 Gardnerella vaginalis
2524023141 Bifidobacterium tsurumiense
2524614804 Bifidobacterium subtile
2524614678 Bifidobacterium thermacidophilum
Bifidobacterium thermophilum
2558860116 Bifidobacterium boum
2524023146 Bifidobacterium minimum
2519899775 Bifidobacterium asteroides
2523533599 Alloscardovia omnicolens
2519103191 Metascardovia criceti
Parascardovia denticolens
647533207 Scardovia inopinata
bin 26
2513020036 Nitriliruptor alkaliphilus
644736322 Acidimicrobium ferrooxidans
bin 57-1
bin 52
bin 57
bin 58
bin 59
bin 29-1
bin 29

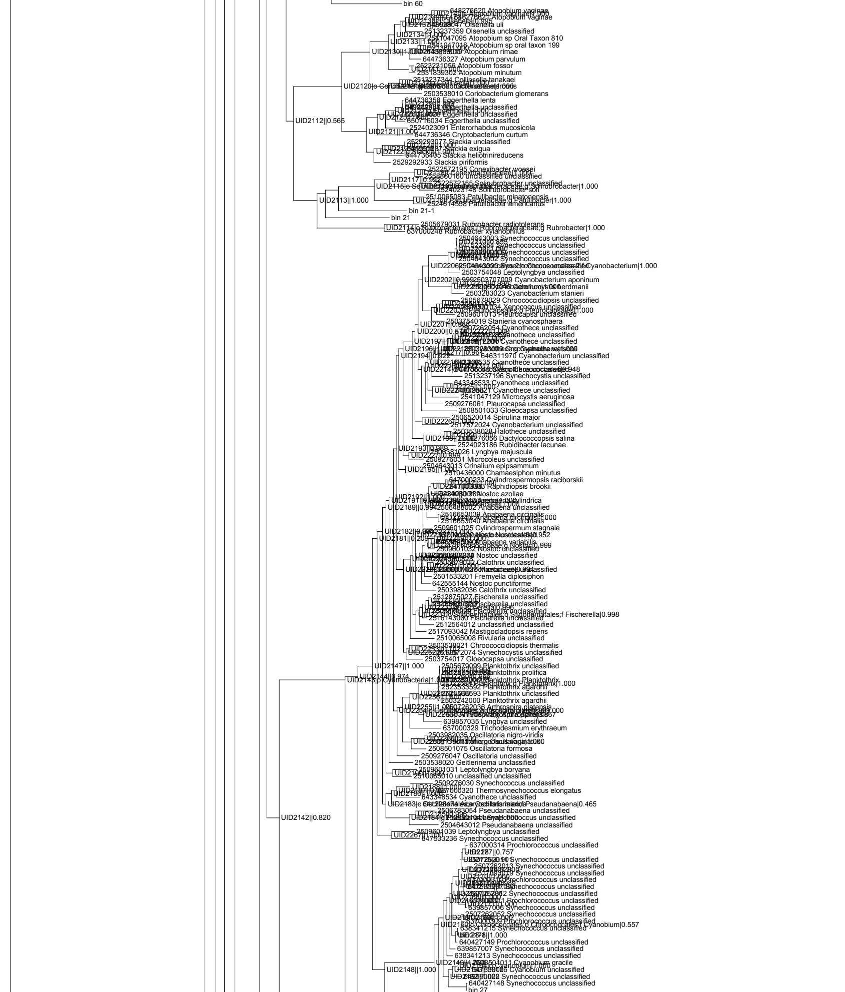

bin 60
648276620 Atopobium vaginae
2513237359 Olsenella unclassified
UID2134||1.000
2541047095 Atopobium sp Oral Taxon 810
2541047018 Atopobium sp oral taxon 199
Atopobium rimae
644736327 Atopobium parvulum
2523231056 Atopobium fossor
2531839302 Atopobium minutum
2513237344 Collinsella tanakaei
2503538010 Coriobacterium glomerans
644736358 Eggerthella lenta
Eggerthella unclassified
650716034 Eggerthella unclassified
2524023091 Enterorhabdus mucosicola
644736346 Cryptobacterium curtum
2529293077 Slackia unclassified
Slackia exigua
644736405 Slackia heliotrinireducens
2529292933 Slackia piriformis
UID2112||0.565
UID2121||1.000
2522572195 Conexibacter woesei
2524023148 Solirubrobacter soli
2510065083 Patulibacter minatonensis
2524614558 Patulibacter americanus
UID2113||1.000
bin 21-1
bin 21
2505679031 Rubrobacter radiotolerans
637000248 Rubrobacter xylanophilus
2504643003 Synechococcus unclassified
2504643002 Synechococcus unclassified
2503754048 Leptolyngbya unclassified
2503707009 Cyanobacterium aponinum
2503283023 Cyanobacterium stanieri
2505679029 Chroococcidiopsis unclassified
2509601013 Pleurocapsa unclassified
2503754019 Stanieria cyanosphaera
2507262054 Cyanothece unclassified
646311970 Cyanobacterium unclassified
2513237196 Synechocystis unclassified
643348533 Cyanothece unclassified
2541047129 Microcystis aeruginosa
2509276061 Pleurocapsa unclassified
2508501033 Gloeocapsa unclassified
2506520014 Spirulina major
2517572024 Cyanobacterium unclassified
2503538028 Halothece unclassified
Dactylococcopsis salina
2524023186 Rubidibacter lacunae
UID2193||0.989
2506381026 Lyngbya majuscula
2509276031 Microcoleus unclassified
2504643013 Crinalium epipsammum
2510436000 Chamaesiphon minutus
647000233 Cylindrospermopsis raciborskii
Raphidiopsis brookii
Nostoc azollae
Anabaena unclassified
2516653039 Anabaena circinalis
2516653040 Anabaena circinalis
2509601025 Cylindrospermum stagnale
2509601032 Nostoc unclassified
Nostoc unclassified
Calothrix unclassified
2501533201 Fremyella diplosiphon
642555144 Nostoc punctiforme
2503982036 Calothrix unclassified
2512875027 Fischerella unclassified
2516143090 Fischerella unclassified
2512564012 unclassified unclassified
2517093042 Mastigocladopsis repens
2510065008 Rivularia unclassified
2503538021 Chroococcidiopsis thermalis
Synechocystis unclassified
2503754017 Gloeocapsa unclassified
UID2147||1.000
UID2144||0.974
UID2143|p Cyanobacteria|1.000
2505679099 Planktothrix unclassified
Planktothrix prolifica
2523533592 Planktothrix agardhii
Planktothrix unclassified
2503242000 Planktothrix agardhii
2507262036 Arthrospira platensis
639857035 Lyngbya unclassified
637000329 Trichodesmium erythraeum
2503982035 Oscillatoria nigro-viridis
2508501075 Oscillatoria formosa
2509276047 Oscillatoria unclassified
2503538020 Geitlerinema unclassified
2509601031 Leptolyngbya boryana
2510065010 unclassified unclassified
2509276030 Synechococcus unclassified
Thermosynechococcus elongatus
643348534 Cyanothece unclassified
2506783054 Pseudanabaena unclassified
2504643012 Pseudanabaena unclassified
UID2142||0.820
2509601039 Leptolyngbya unclassified
647533236 Synechococcus unclassified
637000314 Prochlorococcus unclassified
2507262013 Synechococcus unclassified
Synechococcus unclassified
639857006 Synechococcus unclassified
2507262052 Synechococcus unclassified
640427149 Prochlorococcus unclassified
639857007 Synechococcus unclassified
638341213 Synechococcus unclassified
UID2148||1.000
Cyanobium gracile
Cyanobium unclassified
Synechococcus unclassified
640427148 Synechococcus unclassified
bin 27

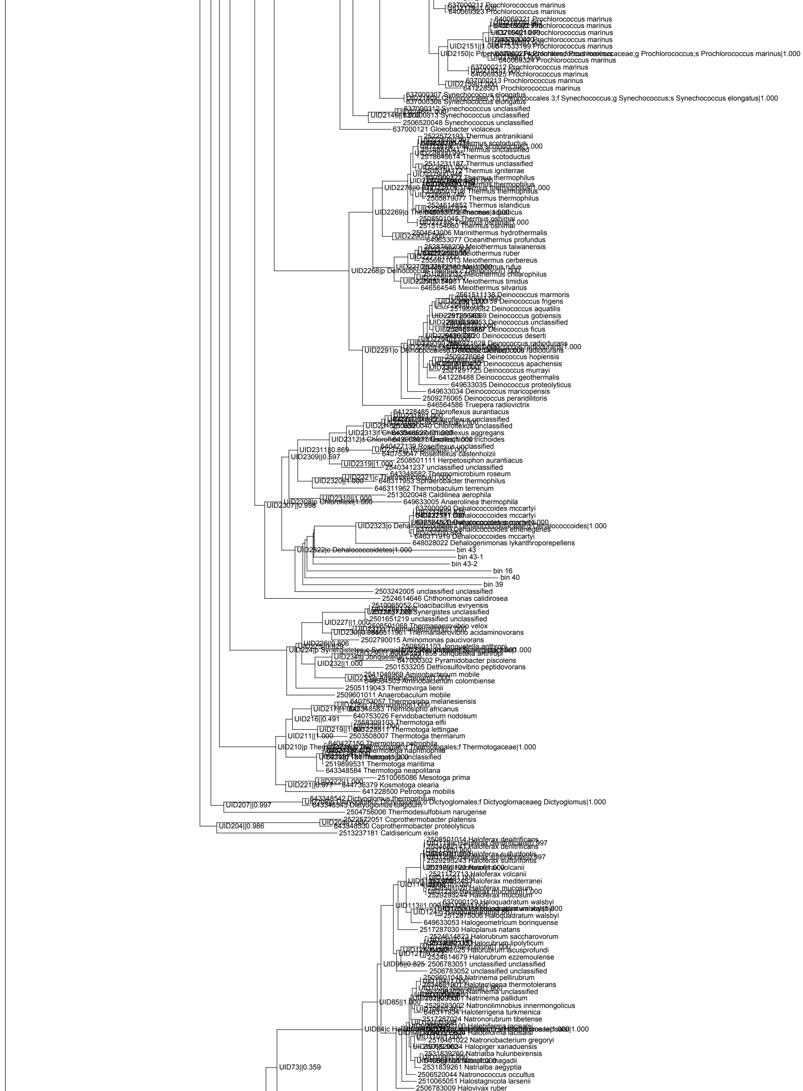

637000211 Prochlorococcus marinus
640069323 Prochlorococcus marinus
640069321 Prochlorococcus marinus
640069324 Prochlorococcus marinus
637000212 Prochlorococcus marinus
640069325 Prochlorococcus marinus
637000213 Prochlorococcus marinus
641228501 Prochlorococcus marinus
637000307 Synechococcus elongatus
637000308 Synechococcus elongatus
637000312 Synechococcus unclassified
2506520048 Synechococcus unclassified
637000121 Gloeobacter violaceus
2522572193 Thermus antranikianii
2518645614 Thermus scotoductus
2511231187 Thermus unclassified
Thermus igniterrae
2505679077 Thermus thermophilus
2524614852 Thermus islandicus
2508501045 Thermus oshimai
2515154080 Thermus oshimai
2504643006 Marinithermus hydrothermalis
649633077 Oceanithermus profundus
2528768209 Meiothermus taiwanensis
Meiothermus ruber
2556921013 Meiothermus cerbereus
Meiothermus rufus
Meiothermus chliarophilus
Meiothermus timidus
646564546 Meiothermus silvanus
2561511138 Deinococcus marmoris
Deinococcus frigens
2519899682 Deinococcus aquatilis
Deinococcus gobiensis
Deinococcus unclassified
Deinococcus ficus
Deinococcus deserti
Deinococcus radiodurans
2509276064 Deinococcus hopiensis
Deinococcus apachensis
2527291725 Deinococcus murrayi
641228488 Deinococcus geothermalis
649633035 Deinococcus proteolyticus
649633034 Deinococcus maricopensis
2509276065 Deinococcus peraridilitoris
646564586 Truepera radiovictrix
641228485 Chloroflexus aurantiacus
Chloroflexus unclassified
Chloroflexus aggregans
Oscillochloris trichoides
640427139 Roseiflexus unclassified
640753047 Roseiflexus castenholzii
2508501111 Herpetosiphon aurantiacus
2540341237 unclassified unclassified
643348582 Thermomicrobium roseum
646311953 Sphaerobacter thermophilus
646311962 Thermobaculum terrenum
2513020048 Caldilinea aerophila
649633005 Anaerolinea thermophila
637000090 Dehalococcoides mccartyi
Dehalococcoides ethenogenes
646311919 Dehalococcoides mccartyi
648028022 Dehalogenimonas lykanthroporepellens
bin 43
bin 43-1
bin 43-2
bin 16
bin 40
bin 39
2503242005 unclassified unclassified
2524614646 Chthonomonas calidirosea
2510065052 Cloacibacillus evryensis
Synergistes unclassified
2501651219 unclassified unclassified
2508501068 Thermanaerovibrio velox
Thermanaerovibrio acidaminovorans
2502790015 Aminomonas paucivorans
2508501103 Jonquetella anthropi
Jonquetella anthropi
647000302 Pyramidobacter piscolens
2501533205 Dethiosulfovibrio peptidovorans
2541046968 Aminobacterium mobile
Aminobacterium colombiense
2505119043 Thermovirga lienii
2509601011 Anaerobaculum mobile
640753057 Thermosipho melanesiensis
Thermosipho africanus
640753026 Fervidobacterium nodosum
2558309103 Thermotoga elfii
Thermotoga lettingae
2503508007 Thermotoga thermarum
640427150 Thermotoga petrophila
Thermotoga naphthophila
2519899531 Thermotoga maritima
643348584 Thermotoga neapolitana
2510065086 Mesotoga prima
644736379 Kosmotoga olearia
641228500 Petrotoga mobilis
643348542 Dictyoglomus thermophilum
643348543 Dictyoglomus turgidum
2504756006 Thermodesulfobium narugense
2522572051 Coprothermobacter platensis
643348530 Coprothermobacter proteolyticus
2513237181 Caldisericum exile
2508501014 Haloferax denitrificans
Haloferax denitrificans
Haloferax sulfurifontis
2523293243 Haloferax sulfurifontis
Haloferax volcanii
2521172713 Haloferax volcanii
Haloferax mediterranei
Haloferax mucosum
2523293244 Haloferax mucosum
637000129 Haloquadratum walsbyi
2512875006 Haloquadratum walsbyi
649633053 Halogeometricum borinquense
2517287030 Haloplanus natans
2524614823 Halorubrum saccharovorum
Halorubrum lipolyticum
Halorubrum lacusprofundi
2524614679 Halorubrum ezzemoulense
2506783051 unclassified unclassified
2506783052 unclassified unclassified
2509601048 Natrinema pellirubrum
Haloterrigena thermotolerans
Natrinema unclassified
Natrinema pallidum
2529293002 Natronolimnobius innermongolicus
646311934 Haloterrigena turkmenica
2517287024 Natronorubrum tibetense
Halobiforma lacisalsi
Halobiforma lacisalsi
2510461022 Natronobacterium gregoryi
Halopiger xanaduensis
2531839260 Natrialba hulunbeirensis
Natrialba magadii
2531839261 Natrialba aegyptia
2506520044 Natronococcus occultus
2510065051 Halostagnicola larsenii
2506783009 Halovivax ruber
UID2151||1.000
UID2150|c Prochlorophyceae;o Prochlorales;f Prochlorococcaceae;g Prochlorococcus;s Prochlorococcus marinus|1.000
Synechococcales 3;f Synechococcus;g Synechococcus;s Synechococcus elongatus|1.000
UID2146||1.000
UID2276||0.
UID2269|o Thermales;f Thermaceae|1.000
UID2290||1.000
UID2268|p Deinococcus-Thermus;c Deinococci|1.000
UID2291|o Deinococcales
UID2313|f Chloroflexaceae|1.000
UID2312|c Chloroflexi;o Chloroflexales|1.000
UID2311||0.869
UID2309||0.597
UID2319||1.000
UID2320||1.000
UID2321|c Thermomicrobia|1.000
UID2310||1.000
UID2308|p Chloroflexi|1.000
UID2307||0.998
UID2323|o Dehalococcoidales;f Dehalococcoidaceae;g Dehalococcoides|1.000
UID2322|c Dehalococcoidetes|1.000
UID227||1.000
UID226||0.806
UID224|p Synergistetes
UID232||1.000
UID217||1.000
UID216||0.491
UID219||1.000
UID211||1.000
UID210|p Thermotogae;o Thermotogales;f Thermotogaceae|1.000
UID221||0.977
UID222||1.000
UID207||0.997
o Dictyoglomales;f Dictyoglomaceae;g Dictyoglomus|1.000
UID204||0.986
UID113||1.000
UID124
UID121
UID96||0.825
UID85||1.000
UID84|c Halobacteria
UID73||0.359

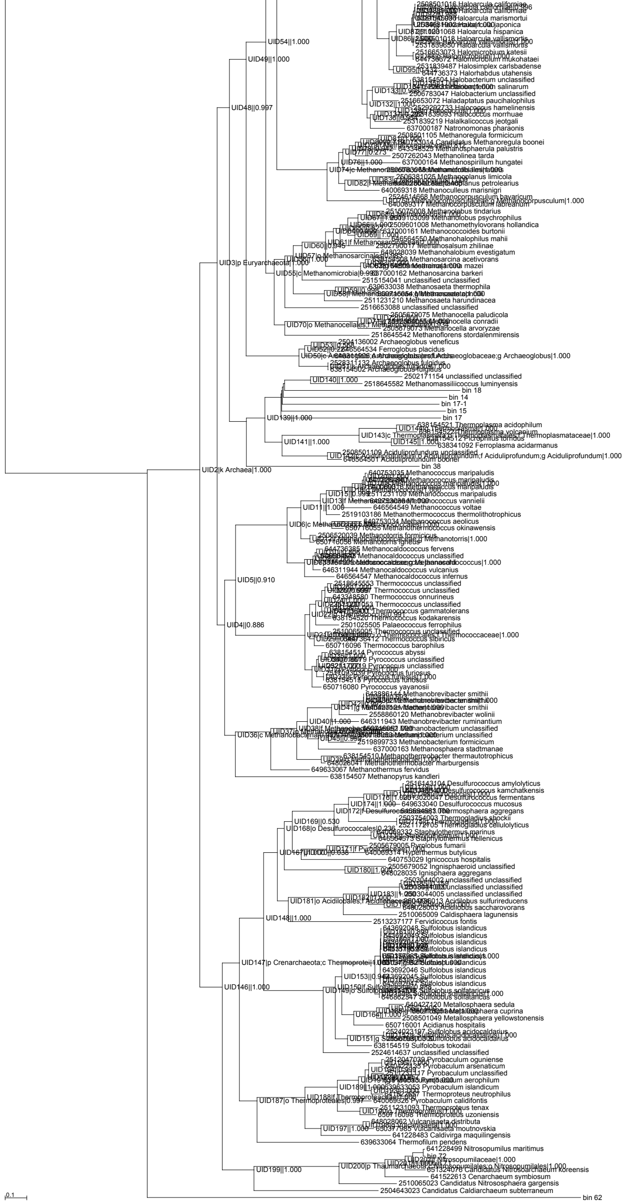

2508501016 Haloarcula californiae
2510201068 Haloarcula hispanica
2531839650 Haloarcula vallismortis
2516653073 Halomicrobium katesii
644736372 Halomicrobium mukohataei
2531839487 Halosimplex carlsbadense
644736373 Halorhabdus utahensis
638154504 Halobacterium unclassified
2506783047 Halobacterium unclassified
2516653072 Haladaptatus paucihalophilus
2531839219 Halalkalicoccus jeotgali
637000187 Natronomonas pharaonis
2508501105 Methanoregula formicicum
2507262043 Methanolinea tarda
637000164 Methanospirillum hungatei
640069318 Methanoculleus marisnigri
2524614668 Methanocorpusculum bavaricum
2515075008 Methanolobus tindarius
2509601008 Methanomethylovorans hollandica
637000161 Methanococcoides burtonii
646564550 Methanohalophilus mahii
2502790017 Methanosalsum zhilinae
648028039 Methanohalobium evestigatum
638154508 Methanosarcina acetivorans
637000162 Methanosarcina barkeri
2515154041 unclassified unclassified
639633038 Methanosaeta thermophila
2511231210 Methanosaeta harundinacea
2516653088 unclassified unclassified
2505679075 Methanocella paludicola
2505679073 Methanocella arvoryzae
2518645542 Methanoflorens stordalenmirensis
2504136002 Archaeoglobus veneficus
646564534 Ferroglobus placidus
2528311132 Archaeoglobus fulgidus
638154502 Archaeoglobus fulgidus
2502171154 unclassified unclassified
2518645582 Methanomassiliicoccus luminyensis
bin 18
bin 14
bin 17-1
bin 15
bin 17
638154521 Thermoplasma acidophilum
638341092 Ferroplasma acidarmanus
2508501109 Aciduliprofundum unclassified
646564501 Aciduliprofundum boonei
bin 38
640753035 Methanococcus maripaludis
2511231109 Methanococcus maripaludis
646564549 Methanococcus voltae
2519103186 Methanothermococcus thermolithotrophicus
640753034 Methanococcus aeolicus
650716055 Methanothermococcus okinawensis
2506520039 Methanotorris formicicus
650716056 Methanotorris igneus
644736385 Methanocaldococcus fervens
646311944 Methanocaldococcus vulcanius
646564547 Methanocaldococcus infernus
2518645553 Thermococcus unclassified
643348580 Thermococcus onnurineus
638154520 Thermococcus kodakarensis
2501025505 Palaeococcus ferrophilus
650716096 Thermococcus barophilus
638154514 Pyrococcus abyssi
650716080 Pyrococcus yayanosii
643886144 Methanobrevibacter smithii
2558860120 Methanobrevibacter wolinii
646311943 Methanobrevibacter ruminantium
2519899733 Methanobacterium formicicum
637000163 Methanosphaera stadtmanae
638154510 Methanothermobacter thermautotrophicus
649633067 Methanothermus fervidus
638154507 Methanopyrus kandleri
2516143104 Desulfurococcus amylolyticus
649633040 Desulfurococcus mucosus
2503754003 Thermogladius shockii
2521172705 Thermogladius cellulolyticus
640069332 Staphylothermus marinus
646564573 Staphylothermus hellenicus
2505679005 Pyrolobus fumarii
640069314 Hyperthermus butylicus
640753029 Ignicoccus hospitalis
2505679052 Ingnisphaeroid unclassified
648028035 Ignisphaera aggregans
2503044002 unclassified unclassified
2503044005 unclassified unclassified
648028003 Acidilobus saccharovorans
2510065009 Caldisphaera lagunensis
2513237177 Fervidicoccus fontis
643692048 Sulfolobus islandicus
643692046 Sulfolobus islandicus
646862347 Sulfolobus solfataricus
640427120 Metallosphaera sedula
2508501049 Metallosphaera yellowstonensis
650716001 Acidianus hospitalis
2524023197 Sulfolobus acidocaldarius
638154519 Sulfolobus tokodaii
2524614637 unclassified unclassified
2512047039 Pyrobaculum oguniense
639633053 Pyrobaculum islandicum
640069326 Pyrobaculum calidifontis
2511231093 Thermoproteus tenax
650716098 Thermoproteus uzoniensis
648028062 Vulcanisaeta distributa
650377985 Vulcanisaeta moutnovskia
641228483 Caldivirga maquilingensis
639633064 Thermofilum pendens
641228499 Nitrosopumilus maritimus
bin 72
651324076 Candidatus Nitrosoarchaeum koreensis
641522613 Cenarchaeum symbiosum
2510065023 Candidatus Nitrososphaera gargensis
2504643023 Candidatus Caldiarchaeum subterraneum
bin 62
UID2|k Archaea|1.000
UID3|p Euryarchaeota|1.000
UID4||0.886
UID5||0.910
UID48||0.997
UID49||1.000
UID54||1.000
UID139||1.000
UID141||1.000
UID146||1.000
UID147|p Crenarchaeota;c Thermoprotei|1.000
UID148||1.000
UID199||1.000
0.1
